# Neoadjuvant adebrelimab in locally advanced resectable esophageal squamous cell carcinoma: a phase 1b trial

**DOI:** 10.1038/s41591-023-02469-3

**Published:** 2023-07-24

**Authors:** Jun Yin, Jingnan Yuan, Yunjin Li, Yong Fang, Ruoxi Wang, Heng Jiao, Han Tang, Shaoyuan Zhang, Siyun Lin, Feng Su, Jianmin Gu, Tian Jiang, Dong Lin, Zhiliang Huang, Chaoxiang Du, Kui Wu, Lijie Tan, Qing Zhou

**Affiliations:** 1 Department of Thoracic Surgery, Cancer Center, Zhongshan Hospital of Fudan University, Shanghai, China; 2 HIM-BGI Joint Lab, Hangzhou Institute of Medicine (HIM), Chinese Academy of Sciences, BGI-Hangzhou, Hangzhou, China; 3 Guangdong Provincial Key Laboratory of Human Disease Genomics, Shenzhen Key Laboratory of Genomics, BGI-Shenzhen, Shenzhen, China; 4 Zhejiang Cancer Hospital, Hangzhou Institute of Medicine (HIM), Chinese Academy of Sciences, Hangzhou, China; 5 Zhongshan Hospital (Xiamen), Fudan University, Xiamen, China

**Keywords:** Cancer immunotherapy, Cancer microenvironment

## Abstract

Overall survival (OS) benefits of neoadjuvant immunotherapy remain elusive in locally advanced esophageal squamous cell carcinomas (ESCC). Here, we reported the results of a phase 1b trial of neoadjuvant PD-L1 blockade with adebrelimab in resectable ESCC. Patients received two neoadjuvant doses of adebrelimab followed by surgery. The primary endpoints were safety and feasibility; secondary endpoints included pathologic complete response (pCR) and OS. Our data showed the primary endpoints of safety and feasibility had been met. Common treatment-related adverse events were anorexia (32%) and fatigue (16%), without grade 3 or more adverse events. Of the 30 patients enrolled in the trial, 25 underwent successful resection without surgery delay and 24% had major pathologic responses including a pCR rate of 8%. The 2-year OS was 92%. Responsive patients had an immune-enriched tumor microenvironment phenotype, whereas nonresponsive patients had greater infiltration of cancer-associated fibroblasts at baseline. Clonotypic dynamics of pre-existing intratumoral T cells was a hallmark of responsive patients. These findings provide a rational for neoadjuvant anti-PD-L1 monotherapy as a therapeutic strategy for patients with resectable ESCC. ClinicalTrials.gov identifier: NCT04215471.

## Abstract (web summary)

In the NATION-1907 trial, treatment of patients with resectable esophageal squamous cell carcinoma with the anti-PD-L1 agent adebrelimab was safe and showed preliminary overall survival efficacy, with responders exhibiting an immune-enriched tumor microenvironment phenotype at baseline.

## Main

Neoadjuvant chemoradiotherapy (nCRT) followed by esophagectomy offered a 23% absolute benefit of long-term survival over 10 years (46% versus 23% with surgery alone, *P* = 0.007) for locally advanced resectable ESCC patients in the CROSS trial^1^. Recently, neoadjuvant chemotherapy (nCT) followed by surgery showed 3-year OS comparable with nCRT among patients with locally advanced ESCC^2^, and both have evolved into standard-of-care treatments^1,3^. However, distant relapse rates remain high, especially in those with residual disease at the time of resection^1^. In addition, adjuvant nivolumab significantly improved disease-free survival (22.4 versus 11.0 months, hazard ratio (HR) 0.69) in esophageal cancer patients who received nCRT but had residual disease at the time of resection in the CheckMate-577 trial, with the greatest benefit seen in early-stage ESCC (29.7 versus 11.0 months, HR 0.61) (ref. ^4^). These results notwithstanding, there remains a need to explore novel multimodality therapeutic strategies to prevent either locoregional progression or distant metastasis.

Neoadjuvant immunotherapy is promising because of its therapeutic efficacy across a variety of solid tumors^5–9^ based on the rationale that broad tumor antigen exposure activates the expansion of more diverse tumor-resident T cell clones before surgery and intensifies systemic surveillance of micro-metastases^10^. Currently, immunotherapy has led to great improvements in first- and second-line settings in advanced-stage ESCC^11,12^, but is yet to be approved in the preoperative setting. The benefit of neoadjuvant immunotherapy combined with chemotherapy or chemoradiotherapy is being explored in several ongoing phase II and III trials that are expected to improve pCR rates^13^; however, a meta-analysis showed that the estimated rates of pCR for immune chemoradiotherapy and immune chemotherapy (32.7% versus 26.3%, *P* = 0.37) were comparable with those of nCRT (35.7%) in our ESCC-based Chinese MIE Interest Study Group (CMISG1701) trial^14^. In addition, data from esophageal adenocarcinoma demonstrated that addition of atezolizumab to nCRT did not improve median OS (29.7 versus 34.3 months, *P* = 0.43) (ref. ^15^). The aforementioned results indicate that the clinical benefits of immunotherapy combined with chemoradiotherapy or chemotherapy remain controversial. More importantly, precision medicine should be guided by an understanding of the mechanisms underpinning a sensitivity and/or resistance evidence-based approach rather than an empirical random combination with available therapies. Therefore, we conducted a single-arm, prospective phase 1b trial (NATION-1907) to investigate the safety profile and preliminary therapeutic efficacy of neoadjuvant programmed death ligand 1 (PD-L1) blockade (adebrelimab) in resectable ESCC, for the first time; to evaluate the exact impact of immunotherapy alone on tumor regression; and to explore the sensitivity/resistance mechanisms, identify the effect-predictive biomarkers and examine the evolving immune response within the tumor microenvironment (TME) during anti-PD-L1 therapy, thereby providing solid evidence for personal tailored treatment targeting the population most suitable for immunotherapy.

Adebrelimab is a high-affinity, humanized monoclonal antibody against PD-L1, which has been demonstrated to be effective and safe in advanced ESCC^16^, extensive-stage small-cell lung cancer^17^ and resectable nonsmall cell lung cancer^18^. In this study, we aimed to: (1) investigate the safety and feasibility of neoadjuvant PD-L1 blockade, and compare recurrence-free survival (RFS) and OS with standard-of-care nCT/nCRT from our CMISG1701 study;^2^ (2) collect clinical and biological evidence to interpret the impact of anti-PD-L1 therapy on tumor regression and TME; (3) identify key molecular features and immune landscape patterns to characterize patients sensitive/resistant to immunotherapy; and (4) define the dynamic, yet nuanced changes occurring in TME during neoadjuvant adebrelimab blockade (briefly named nAde).

## Results

### Patient characteristics

**Fig. 1:**
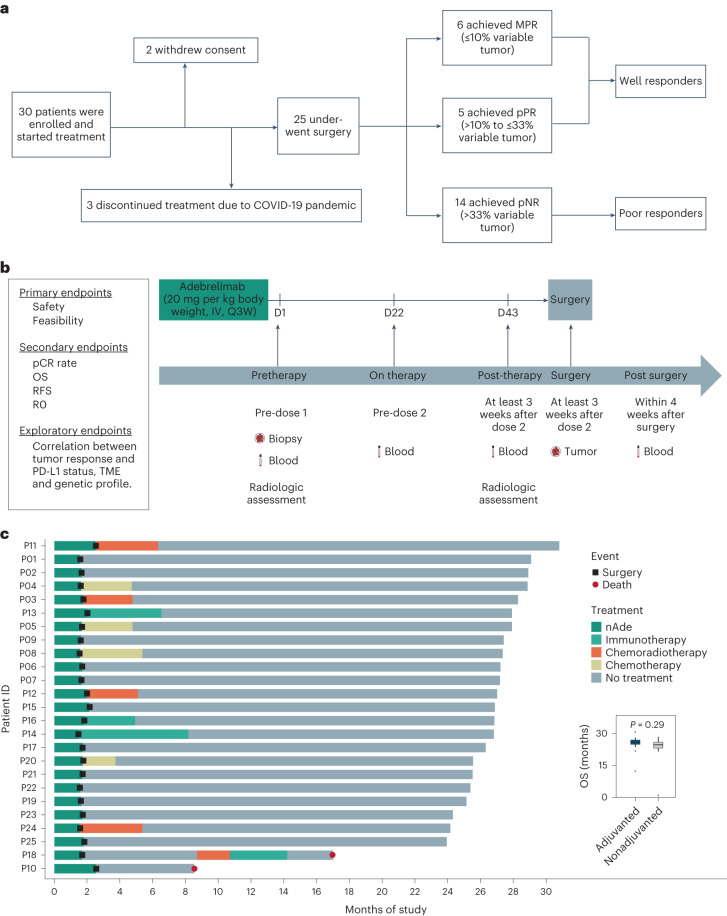
NATION-1907 study design. **a**, NATION-1907 study design. pCR, no viable tumor; MPR, 0% to 10% viable tumor; partial pathologic response (pPR), 10% to 33% viable tumor; pathologic nonresponse (pNR), 33% to 100% viable tumor. **b**, Trial schema. Eligible patients were treated with two doses of neoadjuvant adebrelimab (20 mg per kg body weight, intravenously (IV), every 21 days (Q3W)), followed by surgical resection. Imaging studies were performed using radiological tools before and after immunotherapy. Tumor samples were collected at baseline and at the time of surgery. Longitudinal blood samples were collected at baseline, before dose 2, before surgery and within 4 weeks after surgery if available. D, day of therapy; R0, complete surgical resection. **c**, Treatment regimen in the neoadjuvant and adjuvant settings, and follow-up status per patient (*n* = 25). The boxplot shows OS in patients with (*n* = 12) or without (*n* = 13) adjuvant treatment. Symbols (dot or square) within each bar represent progression events, such as death or surgery. *P* value was calculated by two-sided Wilcoxon rank-sum test. For boxplots the center line and box boundaries represent the median, 25th and 75th percentiles respectively, upper and lower whiskers represent 1.5× interquartile range within the boxes and points indicate outliers. Source data

**Extended Data Fig. 1:**
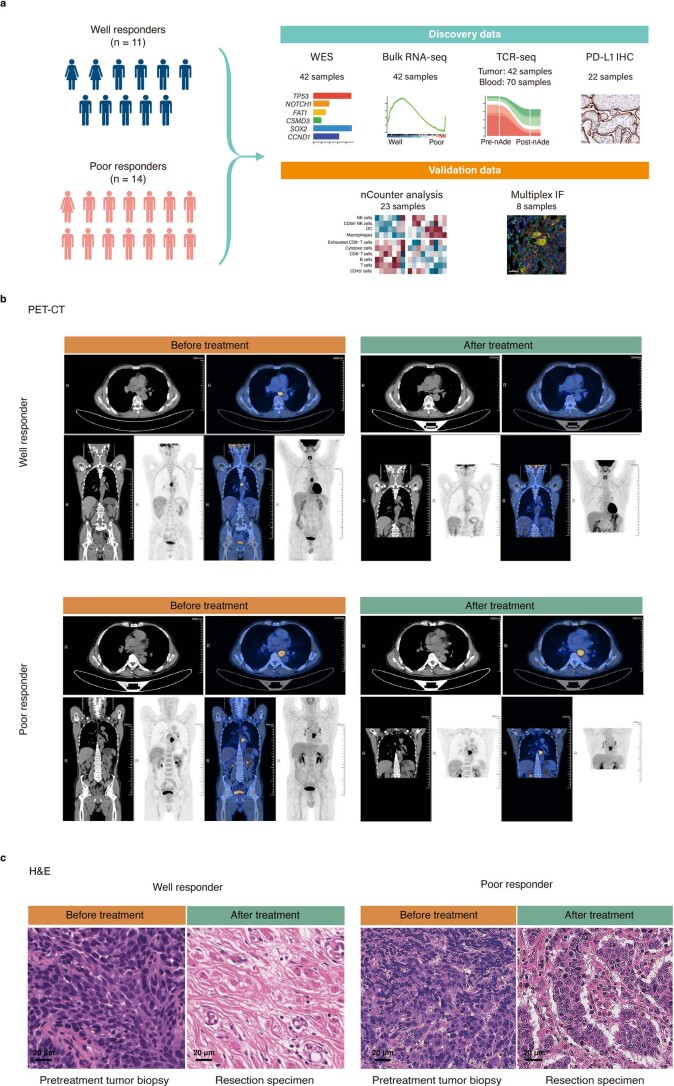
Patterns of radiologic and pathological response. **a**, Multi-omics exploratory study design. **b**, Representative radiologic response before and after neoadjuvant adebrelimab blockade using PET-CT. Top: a well responder (*n* = 1); Bottom: a poor responder (*n* = 1); Left: before treatment; Right: after treatment. **c**, Representative pathological response before and after treatment (Hematoxylin and eosin staining, H&E). Left, a well responder (*n* = 1); right: a poor responder (*n* = 1). Experiment was performed once. PET-CT, Positron Emission Tomography and Computed Tomography Scans. WES, whole-exome sequencing; bulk RNA-seq, whole transcriptomic sequencing; TCR-seq, T cell repertoire sequencing; PD-L1 IHC staining (Dako 22C3 antibody); Multiplex IF, multiple immunofluorescences; *n* reflects the independent number of patients.

Thirty patients were enrolled from 26 December 2019 to 29 August 2020. Of these patients, 25 were eligible for inclusion in the study (Fig. 1a, Table 1 and Supplementary Table 1), all of whom received two doses of neoadjuvant adebrelimab (20 mg kg^−1^, intravenously, every 21 days) (Hengrui Pharmaceuticals) followed by surgery (Fig. 1b). Patients undergoing subsequent adjuvant therapy including chemotherapy, chemoradiotherapy or anti-PD-1 immunotherapy were accepted (Fig. 1c). The study was approved by the institutional ethics committee (B2019-205R) and conducted in accordance with ethical guidelines (Declaration of Helsinki). Among the patients, 80% had stage III disease (*American Joint Committee on Cancer Staging Manual* (*AJCC*), eighth edition), 88% were male and 40% were current or former smokers. Most tumors were in the middle (44%) or distal third (44%) of the esophagus, with a median tumor length of 39.2 mm (range 15.4–70.0 mm) and diameter of 14.9 mm (range 9.9–24.1 mm). Five patients withdrew consent and discontinued the treatment, including two who underwent treatment regimen switching as per patient request and three who refrained from scheduled treatment because of the COVID-19 pandemic. Furthermore, we defined well responders (<33% residual tumor)^19^ and poor responders (>33% residual tumor) to explore candidate patient-stratified biomarkers according to results from a nationwide ESCC study in Japan^20^. We collected tumor tissues and serial peripheral blood before, during and after nAde, and performed whole-exome sequencing (WES), bulk RNA-sequencing (RNA-seq), T cell receptor (TCR) sequencing (TCR-seq) and association analyses with pathological response (Fig. 1b, Extended Data Fig. 1a and Supplementary Table 2).

**Table 1:** Characteristics of the patients at baseline

Characteristics		All patients	Well responders^a^	Poor responders
		(*n* = 25)	(*n* = 11)	(*n* = 14)
**Age at enrollment (years)**				
	Mean ± s.d.	65.2 ± 5.3	65.0 ± 6.1	65.3 ± 4.6
	Median (range)	65 (55, 75)	65 (55, 75)	65 (57, 72)
**Sex (*n*,%)**				
	Female	3 (12)	2 (18)	1 (7)
	Male	22 (88)	9 (82)	13 (93)
**Tumor location (*n*, %)**				
	Proximal third	3 (12)	2 (18)	1 (7)
	Middle third	11 (44)	5 (46)	6 (43)
	Distal third	11(44)	4 (36)	7 (50)
Clinical disease stage^b^ **(*n*, %)**				
	II	3 (12)	2 (18)	1 (7)
	III	20 (80)	8 (73)	12 (86)
	IV	2 (8)	1 (9)	1 (7)
**Tumor length (*x*** ± ***s*, mm)**		40.5 ± 12.7	40.3 ± 12.8	40.6 ± 13.1
**Tumor diameter (*x*** ± ***s*, mm)**		15.3 ± 4.1	15.3 ± 4.3	15.3 ± 4.2
**Smoking status (*n*, %)**				
	Former/current	10 (40)	6 (55)	4 (29)
	Never	15 (60)	5 (45)	10 (71)
**PD-L1 status**^**c**^ **(*n*, %)**				
	CPS > 10	4 (16)	3 (27)	1 (7)
	CPS < 10	18 (72)	7 (64)	11 (79)
	unknown	3 (12)	1 (9)	2 (14)

^a^ Well responders were defined as having 33% or fewer residual viable tumor cells in the resected tumor. Poor responders were defined as having 33% or more residual viable tumor cells in the resected tumor.

^b^ The clinical disease stage was evaluated according to criteria in the *AJCC*, eighth edition.

^c^ PD-L1 status was evaluated by CPS using Dako 22C3 antibody.

### Safety and feasibility

nAde was not associated with any previously unreported toxic effects or severe adverse events (grade 3 or more). Treatment-related adverse events of any grade occurred in 14 of 25 patients (56%); most treatment-related adverse events were mild (grade 1). Common adverse events related to nAde were anorexia (eight patients, 32%), fatigue (four patients, 16%), thrombocytopenia (three patients, 12%), nausea (three patients, 12%), vomiting (two patients, 8%) and anemia (one patient, 4%) (Extended Data Table 1 and Supplementary Table 3). There were no treatment-related surgical delays or deaths within 30 or 90 days after surgery, as defined in the Supplementary study protocol. The median interval between administration of the second dose of adebrelimab and surgery was 26 days (range 22–55 days). Twenty-three patients achieved successful microscopically margin-negative surgical resection (R0 resection), and two patients (P10, P18) failed to achieve R0 resection.

### Preliminary efficacy

**Fig. 2:**
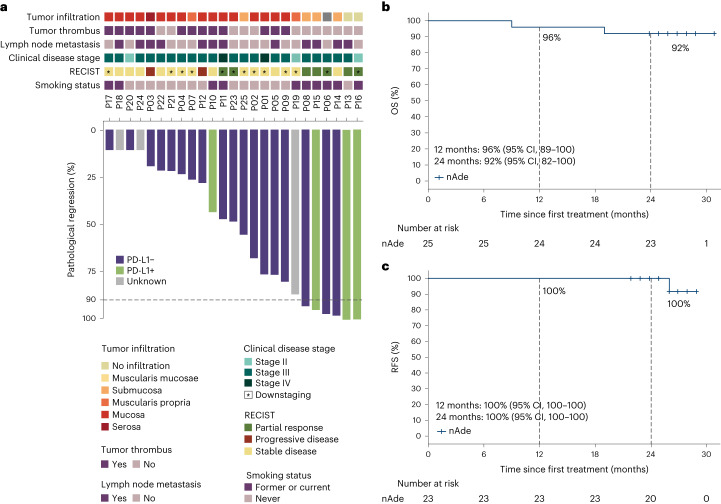
Preliminary efficacy. **a**, Pathological response after nAde. The gray horizontal line indicates the threshold for MPR patients. Clinical and pathological features are annotated for each patient, as is PD-L1 expression (determined by Dako 22C3). **b**,**c**, Kaplan–Meier curves of OS (**b**) and RFS (**c**) for patients who received nAde followed by surgery in the NATION-1907 trial. Median OS and RFS were not reached. Source data

**Extended Data Fig. 2:**
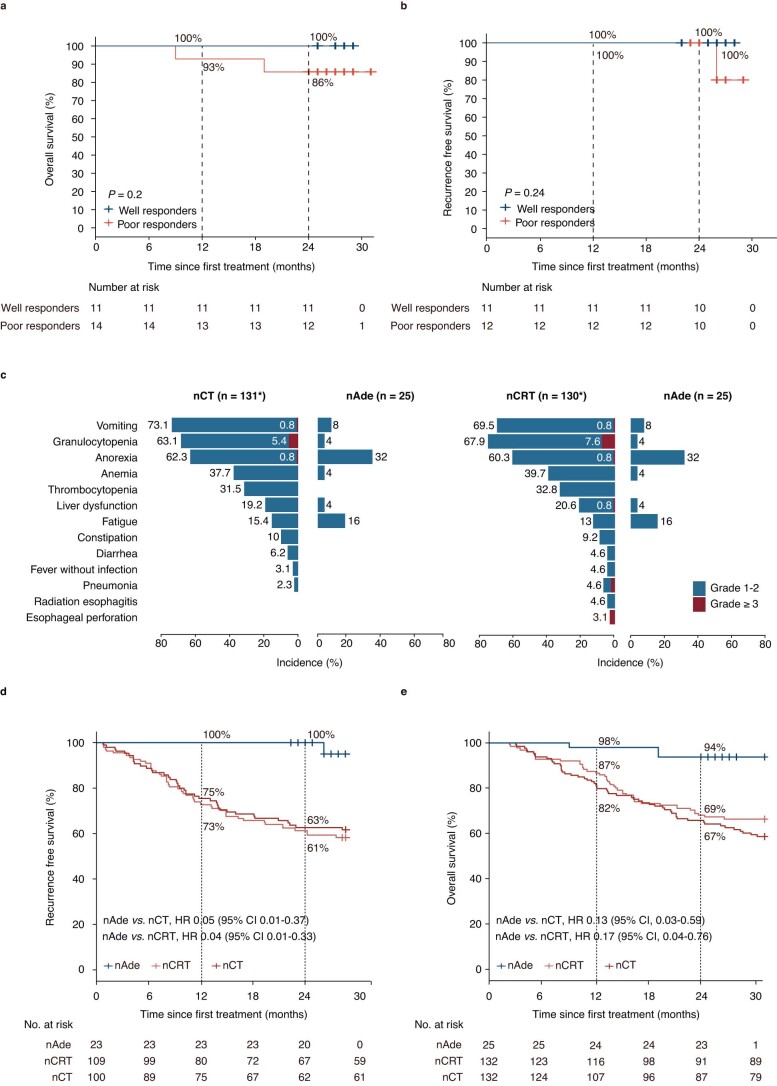
Post hoc comparative analysis with historical CMISG1701 trial. **a**, **b**, Kaplan-Meier curves of overall survival and recurrence free survival between well responders and poor responders in NATION-1907 trial. The numbers of patients at risk at 6-month intervals were included below the x-axis. *P* values were calculated using a two-sided log rank test. **c**, Comparison of treatment-related adverse events in neoadjuvant adebreliamb (nAde) versus neoadjuvant chemotherapy (nCT) and in nAde versus neoadjuvant chemoradiotherapy (nCRT). Grade 3 or more events were labeled in red color, Grade 1 or 2 events were labeled in blue. Adverse events, *One of 132 patients in nCT group and 2 of 132 patients in nCRT group declined to receive treatment. **d,e**, Kaplan-Meier curves of recurrence free survival and overall survival in patients treated with nAde versus historical patients with nCT or nCRT after Inverse probability treatment weighting (IPTW) adjustment, respectively. The 2-year recurrence free survival and overall survival rate were estimated by Kaplan-Meier method. *n* reflects the number of patients. HR, hazard ratio; 95% CI, 95% confidence interval. Source data

Representative radiological and pathological responses after nAde are shown in Fig. 2a and Extended Data Fig. 1b,c. Pathological evaluation showed that 6 of 25 patients (24%) had a major pathologic response (MPR; tumor regression >90%). The median pathological regression was −48% (range, −100 to −10) (Supplementary Fig. 1). In most cases the change in radiographic tumor volume did not reflect the full extent of tumor necrosis. Pathological downstaging from pretreatment clinical stage occurred in 13 patients (52%) (Supplementary Table 4). Of the 25 patients for whom radiographic data was available for evaluation, 7 (28%) had a partial response, 16 (64%) had stable disease and 2 (8%) had disease progression. At the time of data cutoff on 15 August 2022, with a median follow-up of 27 months (range 24–31 months), the 2-year OS and 2-year RFS rates were 92% (95% confidence interval (CI), 82 to 100) and 100% (95% CI, 100 to 100), respectively (Fig. 2b,c). There was no difference in OS for patients with or without adjuvant therapy (including chemotherapy, chemoradiotherapy or anti-PD-1 therapy) (Fig. 1d). In addition, 2-year OS was slightly longer in well responders (100% versus 86%, *P* = 0.2) compared with poor responders (Extended Data Fig. 2a,b).

### Post hoc comparative analysis with historical data

We performed post hoc analyses and compared the OS and RFS data from our study with historical data for patients who received standard-of-care (nCRT or nCT) in our previously published CMISG1701 study. The favorable safety profile in the NATION-1907 trial was in stark contrast to standard therapeutic strategies observed in our CMISG1701 trial, in which nCRT and nCT were associated with 15.3% and 6.9% grade 3 or more treatment-related adverse events, respectively (Extended Data Fig. 2c, Extended Data Table 2 and Supplementary Table 5). After inverse probability of treatment weighting (IPTW) adjustment, the 2-year RFS was 100% and was statistically significantly improved in the NATION-1907 trial, compared with 61% with nCRT (HR 0.04, 95% CI 0.01 to 0.33; *P* = 0.002) and 63% with nCT (HR 0.05, 95% CI 0.01 to 0.37; *P* = 0.003) (Extended Data Fig. 2d and Supplementary Table 6). Similarly, the 2-year OS was 94% and statistically significantly improved with nAde, compared with 69% with nCRT (HR 0.17, 95% CI 0.04 to 0.76; *P* = 0.021) and 67% with nCT (HR 0.13, 95% CI 0.03 to 0.59; *P* = 0.008) (Extended Data Fig. 2e and Supplementary Table 7).

### Genomic features

**Extended Data Fig. 3:**
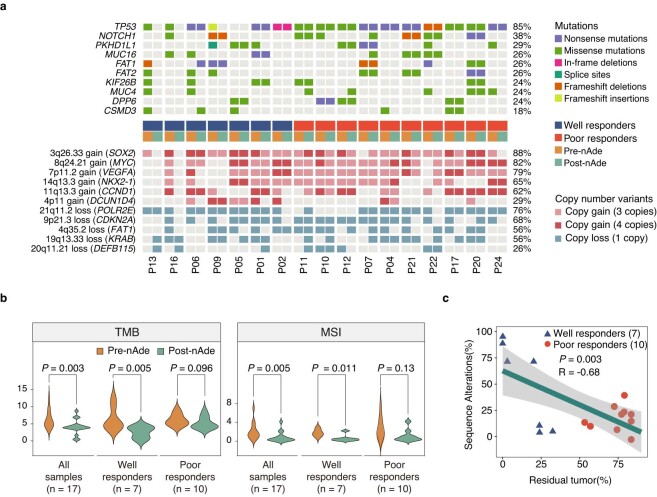
Correlation between genomic biomarkers and pathological response. **a**, Somatic variants in tumor tissues. **b**, Violin plot of TMB and MSI in well responders and poor responders. *P* values were assessed by two-sided Wilcoxon rank-sum test. **c**, Correlation between the number of sequence alterations and percentage of residual tumor cells after anti-PD-L1 treatment (Pearson rho, R = −0.68; *P* = 0.003). The dashed green line indicated the linear regression line. Shaded region represent 95% CI. *P* values from two-sided *t*-test were shown for statistical differences. *n* reflects the independent number of patients. TMB, tumor mutation burden; MSI, microsatellite instability. Source data

We examined the correlation between genomic biomarkers and pathological tumor regression and did not find enriched mutations, mutational signatures or copy number variations in well responders; nor were there differences in tumor mutation burden (TMB) and microsatellite instability (MSI) score (Extended Data Fig. 3a,b and Supplementary Fig. 2). However, a more significant decrease in TMB and MSI score was observed in well responders after anti-PD-L1 treatment. The number of sequence alterations was inversely associated with the proportion of residual tumor (Extended Data Fig. 3c). Predicted tumor neoantigens were positively correlated with TMB (Supplementary Fig. 3). Interestingly, one patient (P20) with 90% residual tumor had human leukocyte antigen (HLA)-A copy number loss along with low expression of HLA-I and HLA-II molecules (Supplementary Fig. 4), which we speculated as being related to the limited benefits via hindering antigen presentation and immune evasion^21^.

### A signature ‘IFN/EMT score’ as potential response biomarker

**Fig. 3:**
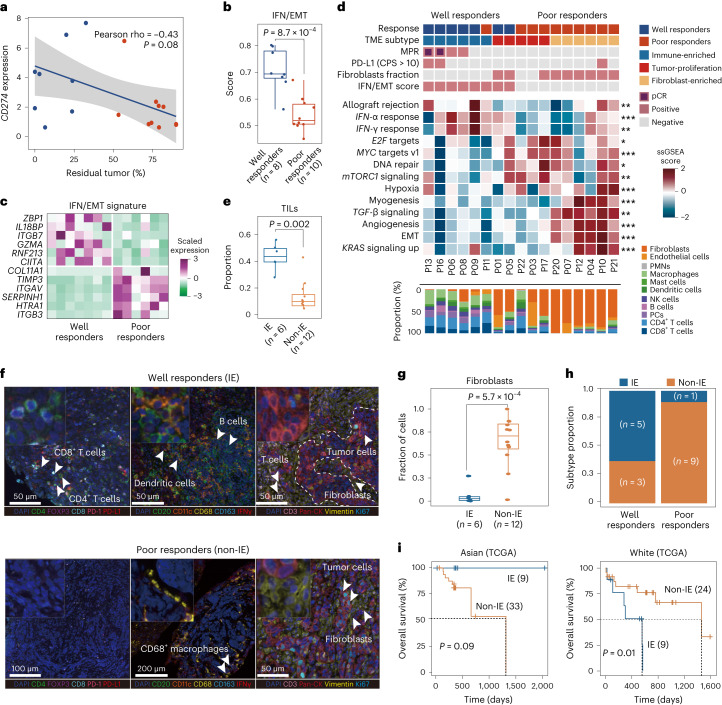
Immune-enriched TME phenotype in responsive patients. **a**, Pearson correlation (two-sided) between mRNA expression of *CD274* and percentage of pathological residual tumor (*n* = 18). Shaded regions represent 95% CI. The *P* value from two-sided *t*-tests is shown for statistical differences. **b**, ‘IFN/EMT score’ using machine learning methods in well and poor responders. **c**, Heatmap for the final 12-gene ‘IFN/EMT score’ defined in the NATION-1907 trial. Well responders, *n* = 7; poor responders, *n* = 6. **d**, Characteristics of IE, tumor-proliferation and fibroblast-enriched TME subtypes determined in the NATION-1907 trial. Clinical and exploratory biomarker features are annotated for each patient. Multiple comparisons were adjusted using the Benjamin–Hochberg method and gene sets of a cancer hallmark with *q* < 0.05 were found to be significantly enriched, as described elsewhere. *q < 0.05, **q < 0.01, ***q < 0.001. *P* values were computed through the two-sided permutation test (*n* = 1,000 randomizations). NK, natural killer cells; PC, plasma cell; PMN, polymorphonuclear cell; ssGSEA, single-sample GSEA. **e**, Proportion of TILs between IE and non-IE subtypes. **f**, Representative multiplex immunofluorescence images from well responders (IE, top, *n* = 2) and poor responders (non-IE, bottom, *n* = 2). The experiment was performed once. **g**, Proportion of fibroblasts in IE and non-IE subtype tumors. **h**, Proportion of IE subtype in well and poor responders. **i**, TME phenotype-guided stratification for OS in the TCGA ESCC Asia and White cohorts. *P* value was performed with two-sided log-rank test. *P* values in **b**, **e** and **g** were derived from a two-sided Wilcoxon rank-sum test. For the boxplots in **b, e** and **g**, the center line and box boundary represent the median, 25th and 75th percentiles respectively, upper and lower whiskers represent 1.5× interquartile range within the boxes and points indicate outliers. *n* indicates the number of patients. Source data

**Extended Data Fig. 4:**
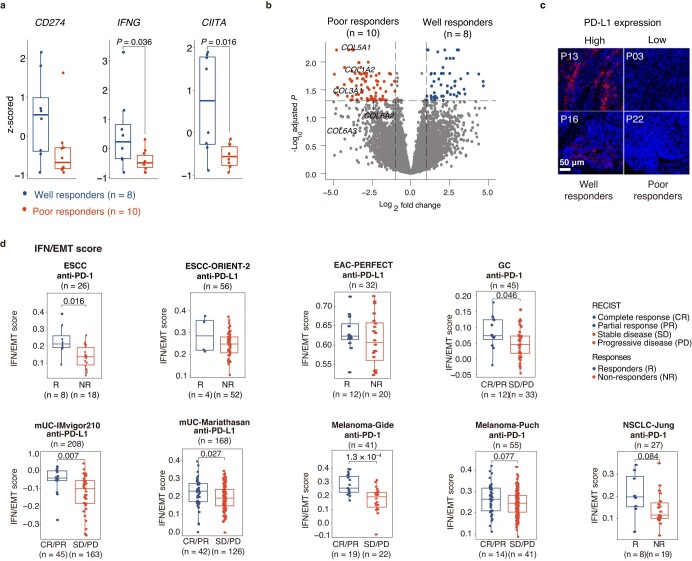
Validation of IFN/EMT score in pan-cancer immunotherapy cohorts. **a**, mRNA expression of *CD274, IFNG, CIITA* between well and poor responders. **b**, Differential expressed genes between well and poor responders. *P* values were derived from two-sided Wald test. The Benjamin-Hochberg method was used to calculate adjusted *P* values for multiple comparisons. **c**, Immunofluorescence images of PD-L1 expression in well (*n* = 2) and poor responders (*n* = 2). Experiment was performed once. **d**, IFN/EMT score validated in previously published pan-cancer immunotherapy cohorts between responders and non-responders (R versus NR, or CR/PR versus SD/PD). *P* values of **a** and **d** were derived from two-sided Wilcoxon rank-sum test. For box plots of **a** and **d**, center line, box boundaries represent median, 25th and 75th percentiles respectively, and upper and lower whiskers represent 1.5 × interquartile range within the boxes and points indicate outlines. *n* reflects the independent number of patients. RECIST, Response Evaluation Criteria in Solid Tumors. ESCC, esophageal squamous carcinoma; EAC, esophageal adenocarcinoma; GC, gastric cancer; mUC, metastatic urothelial carcinoma; NSCLC, non-small cell lung cancer. Source data

To explore the mechanism in response to anti-PD-L1 therapy, we performed bulk RNA-seq in pretreatment tumors. *CD274*, *IFNG* and *CIITA* had relatively higher expression in well responders, whereas collagen-related genes were significantly upregulated in poor responders (Extended Data Fig. 4a,b). *CD274* messenger RNA expression was correlated with pathological tumor regression (Fig. 3a). At the protein level, high PD-L1 expression was also observed in well responders (Extended Data Fig. 4c). We defined a 12-gene signature, named the ‘Interferon/Epithelial–mesenchymal transition (IFN/EMT) score’, which was associated with pathological regression (*P* < 0.001) using a machine learning method (Fig. 3b,c). Similarly, for validation, the ‘IFN/EMT score’ was further shown to be associated with clinical benefits in a variety of advanced disease types (for example, metastatic urothelial cancer, melanoma, gastric cancer and nonsmall cell lung cancer) in patients who received anti-PD-(L)1 treatment (Extended Data Fig. 4d). Overall, the IFN/EMT signature was able to predict the response to immunotherapy.

### Immune-enriched TME phenotype as potential response biomarker

**Extended Data Fig. 5:**
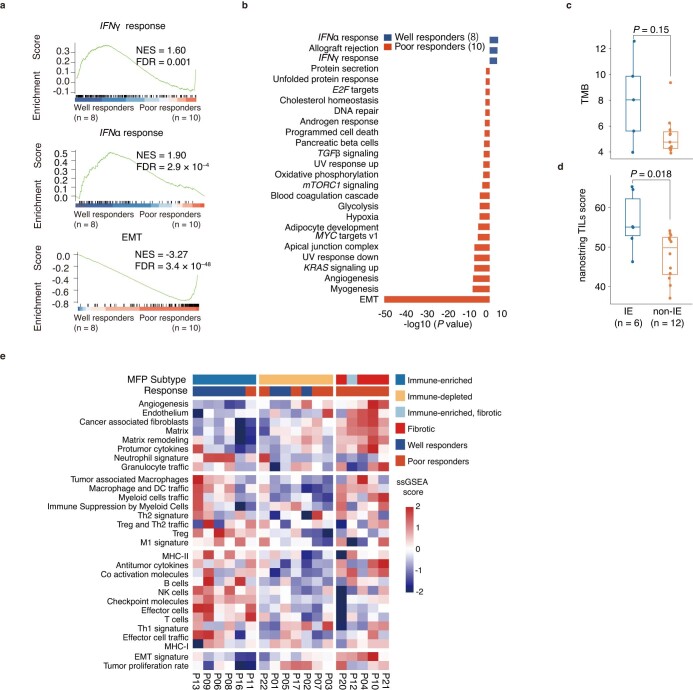
Differential enriched pathways in responsive patients. **a**, **b**, Enriched pathways of cancer hallmarks between well and poor responders. *P* values were computed through the two-sided permutation test (*n* = 1,000 randomizations). The Benjamin-Hochberg method was used to calculate FDR-adjusted *P* value for multiple comparisons. EMT, epithelial-mesenchymal transition. **c**, TMB value difference between IE and non-IE subtype tumors. **d**, TILs infiltration between IE and non-IE subtype tumors using nanostring data. **e**, Heatmap of MFP classification using previously published method (*n* = 18). MFP, Molecular Functional Portrait. *P* values of **c** and **d** were derived from two-sided Wilcoxon rank-sum test. For box plots of **c** and **d**, center line, box boundaries represent median, 25th and 75th percentiles respectively, and upper and lower whiskers represent 1.5 × interquartile range within the boxes and points indicate outliers. IE, Immune-enriched subtype; non-IE, Tumor-proliferation subtype and Fibroblast-enriched subtype. *n* reflects the independent number of patients. Source data

Next, based on immune cell abundance and the enrichment pathway of cancer hallmarks from the Molecular Signatures Database between well and poor responders (Extended Data Fig. 5a,b), we defined three different TME subgroups: ‘immune-enriched’ (IE), ‘tumor-proliferation’ and ‘fibroblast-enriched’ (Fig. 3d). The IE subgroup, characterized as the inflamed TME phenotype, consisted of patients with higher numbers of tumor-infiltrating lymphocytes (TILs) (Fig. 3e), high *IFN-*γ and *IFN-*α activity (Fig. 3d) and higher TMB (Extended Data Fig. 5c). TIL infiltration was further confirmed by multiplex immunofluorescence staining and an immune gene panel (Fig. 3f, Extended Data Fig. 5d and Supplementary Fig. 5). Non-immune-enriched (non-IE) subgroups, including the tumor-proliferation and fibroblast-enriched subtypes, were characterized as the immunosuppressive TME phenotype. The tumor-proliferation subtype showed rare TIL infiltration and significant activation of *E2F* targets, *MYC* targets, and DNA repair and m*TORC1* pathways, suggesting tumor cell proliferation. The fibroblast-enriched subtype exhibited a high proportion of fibroblasts, and high activity for epithelial–mesenchymal transition (EMT), *KRAS* and transforming growth factor beta (*TGF*-β) signaling, and angiogenesis pathways, consistent with a previous study^22^. Fibroblast infiltration in pretreatment tumors was further confirmed by multiplex immunofluorescence and bulk RNA-seq (Fig. 3f,g). Our TME phenotype classification was highly consistent with the pan-cancer microenvironment subtype in predicting response to immunotherapy (Extended Data Fig. 5e)^23^.

**Extended Data Fig. 6:**
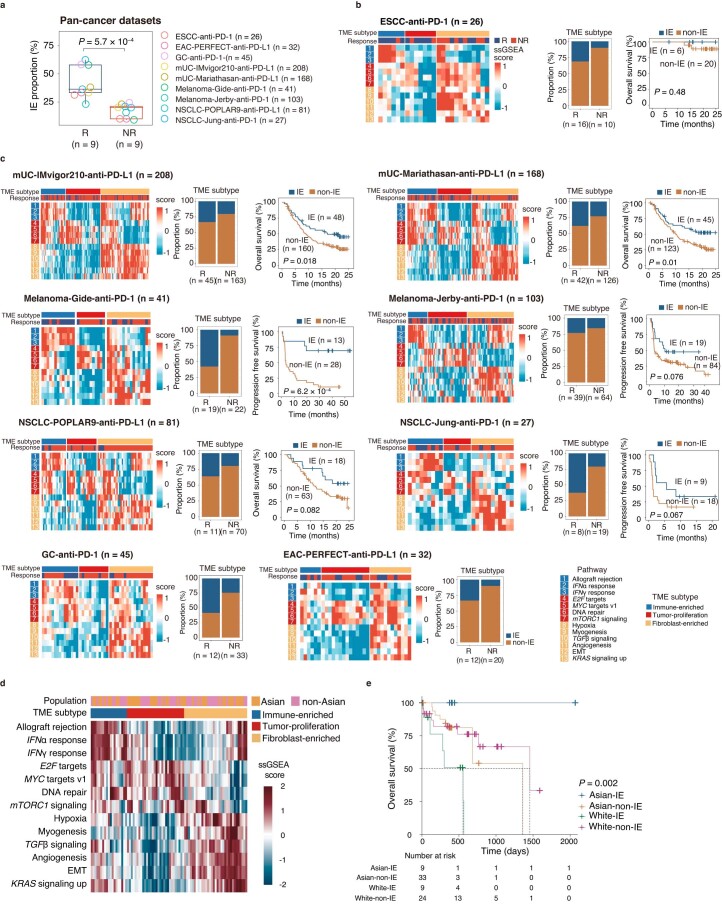
Validation of TME phenotype in pan-cancer immunotherapy cohorts. **a**, Proportions of IE subtype between responders and non-responders in pan-cancer immunotherapy datasets. *P* values was assessed by two-sided Wilcoxon rank-sum test. For boxplot, center line, box boundaries represent median, 25th and 75th percentiles respectively, and upper and lower whiskers represent 1.5 × interquartile range within the boxes and points indicate outliers. **b, c**, Correlation of TME subtype and clinical benefits in pan-cancer immunotherapy datasets. Left panel, heatmap of TME subtype using 13 pathways of cancer hallmarks identified in the NATION-1907 trial; Middle panel, proportion of IE subtype in multiple cancer types; Right panel, Kaplan-Meier overall survival and progression free survival in patients with IE and non-IE subtype. **d**, Heatmap of TME phenotype in TCGA-ESCC cohort (Asia, *n* = 42, non-Asia, *n* = 40) using 13 pathways identified in the NATION-1907 trial. **e**, Kaplan-Meier overall survival of patients with IE and non-IE subtype in TCGA-ESCC Asian cohort (*n* = 42) and TCGA-ESCC White cohort (*n* = 33). *P* values of **b, c** and **e** was calculated using two-sided log rank test of the Kaplan-Meier estimation of overall survival and progression free survival rate. *n* reflects the independent number of patients. R, responders; NR, non-responders; ESCC, esophgeal squamous carcinoma; EAC, esophageal adenocarcinoma; GC, gastric cancer; mUC, metastatic urothelial carcinoma; NSCLC, non-small cell lung cancer. IE, immune-enriched subtype; non-IE, Tumor-proliferation subtype and Fibroblast-enriched subtype. Source data

Tumors of the IE subtype exhibited more pathological tumor regression than non-IE subtypes, as exemplified by the finding that IE subtype was detected in 62.5% (5/8) of well responders compared with only 10% (1/10) of poor responders (Fig. 3h). Tumors of the non-IE subtype were enriched in poor responders. We further validated that patients with the IE subtype had significantly prolonged OS and progression-free survival in nine previously published pan-cancer immunotherapy datasets (*P* < 0.001) (Extended Data Fig. 6a–c). Also, patients with the IE subtype showed longer OS in The Cancer Genome Atlas (TCGA) ESCC Asia cohort, in contrast to the White cohort (Fig. 3i and Extended Data Fig. 6d,e), which was in line with a divergence in survival benefits in the KEYNOTE-590 and RATIONALE-302 trials. Overall, we demonstrated that the TME phenotype of ESCC could stratify patients by response to immunotherapy.

### Recruitment of immune-suppressive cells in poor responders

**Fig. 4:**
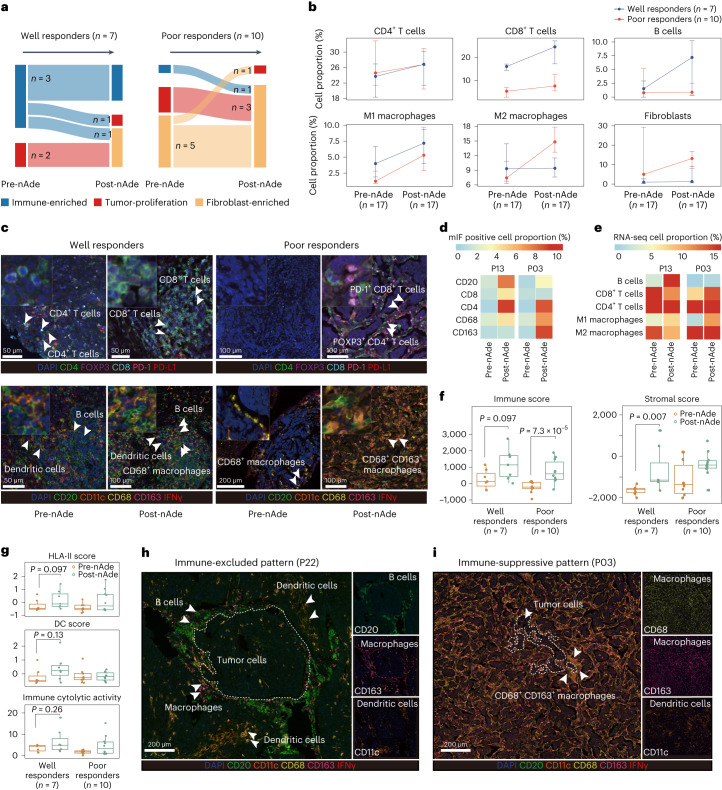
Dynamic evolution of the TME status in response to nAde. **a**, Dynamic of TME subtypes during nAde. **b**, Proportion of TILs and fibroblasts before and after treatment. Points represent median values, whereas whiskers show the upper and lower quantiles. **c**, Representative multiplex immunofluorescence images of patients before and after treatment. Well responder (*n* = 1, P13); poor responder (*n* = 1, P03). The experiment was performed once. **d**,**e**, Heatmaps showing (**d**) the proportion of immune cells identified by multiplex immunofluorescence (mIF) images and (**e**) the relative cell proportion estimated by deconvolution analysis using bulk RNA-seq data. The color represents the scaled cellular proportion. **f**, Changes in the immune score and stromal score signature during nAde. **g**, Changes in HLA-II score, DC score and immune cytolytic activity between well and poor responders. **h**,**i**, Representative multiplex immunofluorescence images of (**h**) the immune-excluded pattern (*n* = 1, P22) and (**i**) the immune-suppressive pattern (*n* = 1, P03) in poor responders. The experiment was performed once. *n* is the independent number of patients. *P* values in **f** and **g** were calculated using a two-sided Wilcoxon rank-sum test. For the boxplots in **f** and **g**, the center line and box boundaries represent the median, 25th and 75th percentiles respectively, upper and lower whiskers represent 1.5× interquartile range within the boxes and points indicate outliers. Source data

To investigate TME dynamics during neoadjuvant PD-L1 blockade, we evaluated the changes in TME phenotype, chemokines and tumor-infiltrating immune cells. In total, 50% (3/6) of the IE subtype showed an overall anti-tumor response trend toward the non-IE subtype after nAde (Fig. 4a). We found that immunologically ‘cold’ (non-IE) tumors failed to turn into ‘hot’ tumors (IE). Tumors with a non-IE subtype showed an overall response trend toward the fibroblast-enriched subtype. Also, we found that CD4^+^ T cells, CD8^+^ T cells, B cells and M1-like macrophages increased in well responders. By contrast, immune-suppressive cells, such as tumor-promoting M2-like macrophages and fibroblasts, increased in poor responders after nAde (Fig. 4b). Of note, multiplex immunofluorescence further confirmed that large numbers of PD1^+^ CD8^+^ T cells, FOXP3^+^ CD4^+^ T_reg_ cells and CD68^+^ CD163^+^ macrophages (M2 macrophages) were recruited into tumors in poor responders after nAde (Fig. 4c,d), consistent with the trend for a shift in immune cells described by bulk RNA-seq (Fig. 4e). The immune score and stromal score signature were significantly increased after nAde (Fig. 4f). In addition, we found that expression of HLA-II score, dendritic cell (DC) score and immune cytotoxic activity did not alter in poor responders after nAde (Fig. 4g), suggesting that a loss of function in presentation of the tumor antigen might occur in poor responders.

### Spatial resolved immune ‘cold’ patterns

The spatial distribution of TILs recruited into tumors after nAde was carefully examined using multiplex immunofluorescence in two poor responders (P22 and P03), both with non-IE phenotypes. We observed two types of distinct immune ‘cold’ patterns. The first was an ‘immune-excluded’ pattern in patient P22. Tumor cells gathered in a large area and there was large distance between immune cells and tumor cells. In addition, CD20^+^ B cells, CD11c^+^ dendritic cells and CD163^+^ macrophages were distributed along the tumor margin but excluded from the tumor core (Fig. 4h). The second was an ‘immune-suppressive’ pattern in patient P03. Immunosuppressive cells and tumor cells both gathered at a relatively short distance, and CD68^+^ CD163^+^ macrophages (tumor-promoting M2 macrophages) were diffusely distributed within tumor cores and located close to tumor cells after PD-L1 blockade (Fig. 4i). In patient P03, the distribution pattern of spatial T cells switched after nAde, recruited tumor-reactive T cells such as PD-1^+^ CD8^+^ T cells and suppressive FOXP3^+^ CD4^+^ T_reg_ cells were distributed in the tumor margin and rarely infiltrated into tumors (Fig. 4c). The results suggested that M2 macrophages and T_reg_ cells may be recruited along with CD8^+^ T cells and further restrain CD8^+^ T cell migration to tumor sites through a long-lasting interaction^24^.

### Pre-existing intratumoral T cells and clinical efficacy

**Fig. 5:**
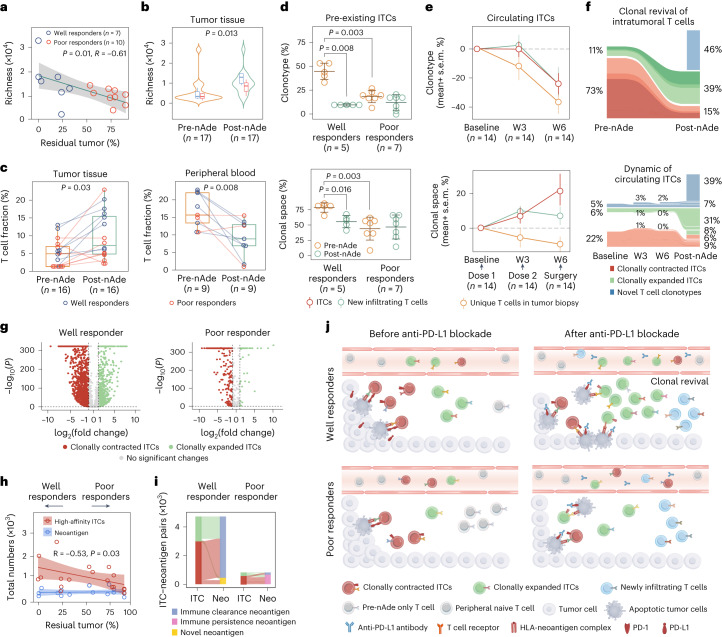
Mechanism of pre-existing T cells in response to nAde. **a**, Pearson correlation (two-sided) between productive T cell richness and pathological responses in the post-treatment tumor. Shaded region represents 95% CI. **b**, Productive richness and clonality of the TCR repertoire before and after treatment (well responders, *n* = 7; poor responders, *n* = 10). **c**, T cell fraction in tumor tissues and peripheral blood before and after treatment using WES data. **d**, Proportion of clonotype and clonal space for pre-existing ITCs. T cell clonal space is defined as the sum frequency of clones relative to the total T cell repertoire. Error bars indicate mean ± s.d. **e**, Temporal dynamics of circulating ITCs before, during and after therapy. Data show the mean ± s.e.m. W3, week 3; W6, week 6. **f**, Representative Sankey plot (*n* = 1, P13) showing clonal space of ITCs and new infiltrating T cells in tumor (upper) and peripheral blood (lower). **g**, Clonotypic dynamic (clonal expansion and contraction) of pre-existing ITCs during PD-L1 blockade, Representative examples of a well responder (left, P13) and a poor responder (right, P17). *P* values were calculated using a two-sided Fisher’s exact test. **h**, Pearson correlation (two-sided) between the number of high-affinity ITCs or neoantigens and the pathological response (*n* = 17). Shaded regions represent 95% CI. **i**, Representative Sankey diagrams of the relationship between ITCs and neoantigens in a well responder (left bar) and a poor responder (right bar). Neo, neoantigen. **j**, Sketch map of the process for the clonal replacement of ITCs and new infiltrated T cells. Mechanism for tumor-reactive T cells: (1) pre-existing ITCs were associated with immunotherapy efficacy; and (2) local expansion of pre-existing ITCs and new infiltrating T clonotypes involved in the response to immunotherapy, a phenomenon termed clonal revival. *P* values in **a** and **h** were calculated using a two-sided *t*-test, whereas in **b**, **c** and **d** *P* values were calculated using a two-sided Mann–Whitney test. For the boxplots in **b** and **c**, the center line and box boundaries represent the median, 25th and 75th percentiles respectively, upper and lower whiskers represent 1.5× interquartile range within the boxes and points indicate outliers. *n* is the independent number of patients. Source data

**Extended Data Fig. 7:**
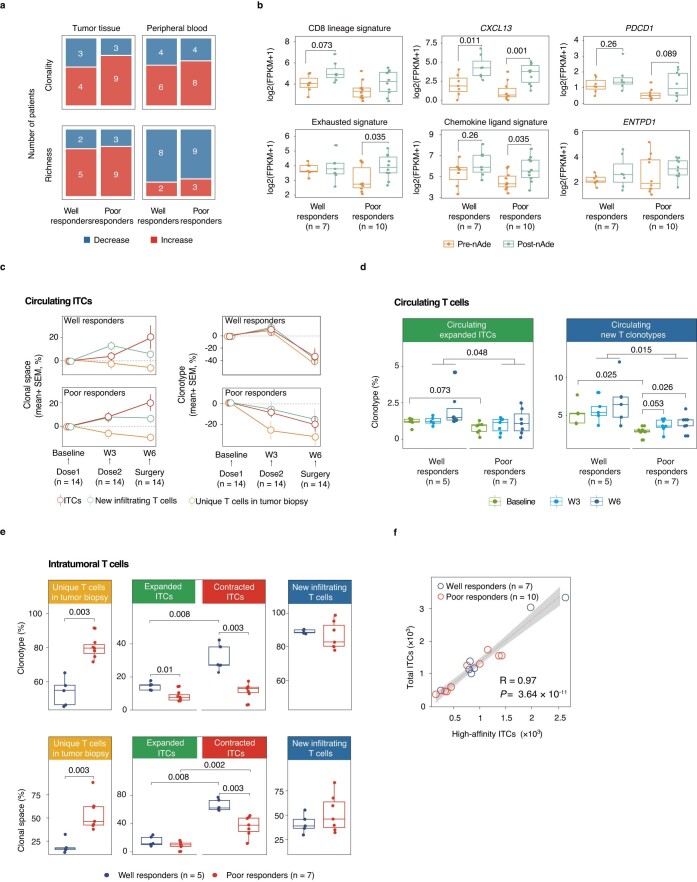
TCR repertoire in peripheral blood and tumor tissues. **a**, TCR repertoire clonality (top) and richness (bottom) after treatment in tumor tissues and peripheral blood. **b**, Changes of signature score related to tumor-reactive T cells between well responders and poor responders. **c**, Temporal dynamics of ITCs in peripheral blood before, on and after therapy. Data show the clonal space and clonotypes with mean ± s.e.m. for well (top) and poor responders (bottom). **d**, Clonotypes dynamics of expanded ITCs and circulating new T clones in peripheral blood during anti-PD-L1 treatment. Circulating expanded ITCs were defined as shared same clonotypes between peripheral T cell repertoire and clonally expanded ITCs, suggesting expanded ITCs who also existed in peripheral blood. Circulating new T clonotypes were defined as shared same clonotypes between peripheral T cell repertoire and new T clonotypes in the posttreatment tumors, suggesting new T clonotypes who also existed in peripheral blood. **e**, Clonotypes and clonal space of clonally expanded ITCs, contracted ITCs and new T clones in tumors. **f**, Correlation between total ITCs and high-affinity ITCs, Pearson rho, 0.97; *P* < 0.001. Shaded region represent 95% CI. *P* value from two-sided *t*-test was shown for statistical differences. *P* values of **b, d** and **e** were derived from two-sided Wilcoxon rank-sum test. *n* reflects the independent number of patients. For boxplot of **b, d** and **e**, center line, box boundaries represent median, 25th and 75th percentiles respectively, and upper and lower whiskers represented 1.5 × interquartile range within the boxes and points indicate outliers. Source data

To assess the temporal dynamics of intratumoral and peripheral T cells in response to neoadjuvant PD-L1 blockade, TCR-β sequencing was performed in serial peripheral blood and tumor tissue. Intratumoral TCR diversity was significantly increased in well responders and was positively correlated with the pathological response at the time of surgery compared with poor responders (Fig. 5a,b). Concordantly, T cell abundance and clonality increased (Figs. 4b and 5c and Extended Data Fig. 7a), suggesting diverse infiltration of new T cell clonotypes into tumor sites after nAde. Peripheral T cell fraction and richness decreased (Fig. 5c and Extended Data Fig. 7a), which was speculated as being associated with treatment-related adverse events of immunotherapy^25^. Of note, pre-existing intratumoral T cells (ITCs, defined as sharing same clonotypes between pretreatment and post-treatment tumors) had a significantly larger fraction of clonotypes (44% versus 20%, *P* = 0.003) and clonal space (81% versus 45%, *P* = 0.003) in well responders (Fig. 5d), accompanied by higher expression of a signature related to tumor-reactive T cells (Extended Data Fig. 7b)^26^, indicating that pre-existing ITCs might be tumor-reactive T cells and neoadjuvant anti-PD-L1 therapy induced drastic clonal replacement in well responders.

These ITCs were also detected in peripheral blood (ITCs in peripheral blood were termed circulating ITCs) and showed clonal expansion in peripheral blood three weeks after the first dose of adebrelimab, reaching a peak before surgery (week 6), accompanied by continuously decreased clonotypes of circulating ITCs (Fig. 5e and Extended Data Fig. 7c). In addition, approximately 73% of ITCs in pretreatment biopsies experienced strong clonal contraction (Fig. 5f), and were speculated to be terminal exhausted T cells with reduced replicative capacity^27^. Furthermore, we classified individual ITCs as being of an expanded or contracted clonotype based on the frequency change before and after nAde. Notably, 85% of T cell clonotypes in the post-treatment tumors were derived from clonally expanded ITCs and novel T clonotypes, half of which were also detected in peripheral blood (Fig. 5f and Supplementary Fig. 6) and showed increasing diversity in the circulation (Extended Data Fig. 7d). Meanwhile, we observed evidence of cytotoxicity for activated T cells in responsive tumors after nAde with a relative increase in immune cytotoxic activity (*GAMB*, *PRF1*) and chemokine ligands signature (*CXLC9*, *CXCL10*) (Fig. 4g and Extended Data Fig. 7b), which may be crucial for the recruitment of circulating T cells^28^. These results suggested that two main sources of tumor-reactive T cells (pre-existing clonally expanded ITCs and newly infiltrating T cell clonotypes) may be reinvigorated upon blockade of the PD-1–PD-L1 axis in tumor sites and be recruited from peripheral sources.

### Clonotypic dynamics of ITCs were hallmark of responders

Of note, paired ITC analyses of baseline and resected tumors from individual patients revealed that clonotypic dynamics (clonally expanded and contracted ITCs) were a hallmark of responsive patients. These dynamic shifts in intratumoral TCRs were reflective of clinical outcomes, in that more significantly clonal expanded and contracted ITCs were observed in well responders compared with poor responders (Fig. 5g, Extended Data Fig. 7e and Supplementary Fig. 7). In addition, the predicted high-affinity ITCs specific for tumor neoantigens in patients were positively associated with clinical benefit (*P* = 0.03; Fig. 5h and Extended Data Fig. 7f). In well responders, the majority of these ITCs recognized tumor neoantigens eliminated by the immune system (named the immune clearance neoantigen) (Fig. 5i and Supplementary Fig. 8). By contrast, poorly responsive tumors had fewer ITCs that recognized the immune clearance neoantigen, suggesting insufficient TCR–HLA–neoantigen recognition to induce complete immune clearance. Taking these discoveries together, we propose a fundamental mechanism for the T cell response to anti-PD-L1 therapy: (1) sufficient clone size of pre-existing tumor-reactive T cells at baseline may directly determine immunotherapy efficacy; (2) tumor-reactive T cells induced by nAde consist of (a) local expansion of pre-existing ITCs and (b) new clonotypes in post-treatment tumors replenished by peripheral T cells or T cells with other origins such as draining lymphoid nodes, a phenomenon termed clonal revival; and (3) the anti-PD-L1 blockade simultaneously induced strong clonal contraction of pre-existing ITCs (Fig. 5j).

## Discussion

Neoadjuvant PD-L1 blockade was well tolerated with a lower incidence of treatment-related adverse events compared with standard-of-care chemotherapy/chemoradiotherapy in resectable ESCC, consistent with observations in lung cancer^7,29^ and advanced esophageal cancer^12,30–34^. In this trial, immunotherapy alone caused no serious adverse events (grade ≥3), in contrast to chemoimmunotherapy which had an incidence of treatment-related adverse events of 47% (CheckMate-648) (ref. ^30^), 59.9% (ORIENT-15) (ref. ^31^), 63.4% (ESCORT-1^st^) (ref. ^33^) and 72% (KEYNOTE-590) (ref. ^34^). Even compared with current standard nCT or nCRT therapies from our own CMISG1701 trial, the treatment-related adverse events following immunotherapy alone were mild. These data support a favorable safety profile for immunotherapy alone for ESCC in the neoadjuvant setting.

**Extended Data Fig. 8:**
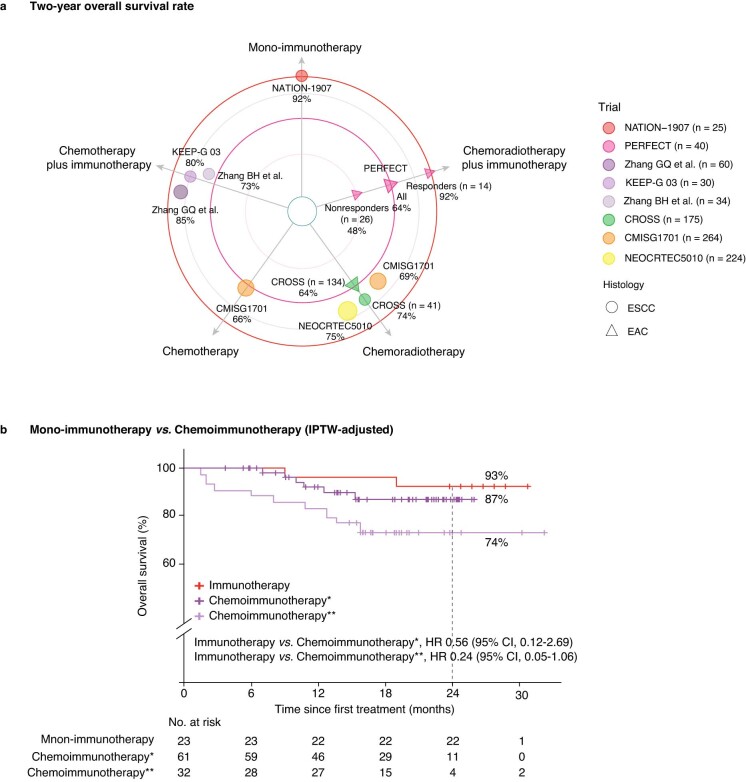
Clinical efficacy comparison of neoadjuvant therapeutic strategy in esophageal cancers. **a**, 2-year of overall survival of esophageal cancer patients in different clinical trials in the neoadjuvant settings; ESCC, esophageal squamous carcinoma; EAC, esophageal adenocarcinoma. **b**, Kaplan-Meier curves of overall survival in patient with neoadjuvant mono-immunotherapy versus chemoimmunotherapy after IPTW adjustment. 24-month overall survival was 93% (95% CI, 83 to 100) in mono-immunotherapy, compared with 87% (95% CI, 78 to 97) in chemoimmunotherapy* and 74% (95% CI, 60 to 91) in chemoimmunotherapy **, respectively; *, chemoimmunotherapy results from Zhang GQ et al. (Refs. ^43^), **, chemoimmunotherapy results from Zhang BH et al (Ref. ^42^). *n* reflects the patient’s number. HR, hazard ratio. Source data

Considering the tumor response, despite small studies of nCT^35^ or chemoradiotherapy^36^ that reported a higher pCR rate, we argue that this advantage of pCR over immunotherapy may not necessarily translate to a survival benefit, because it can be largely compensated for by radical resection^37^. The CMISG1701 trial demonstrated that nCRT and nCT resulted in a comparable 1-year OS (82.6% versus 87.1%, *P* = 0.1) and 3-year OS (64.1% versus 54.9%, *P* = 0.28) despite a significantly different pCR rate (3.8% versus 35.7%, *P* = 0.001) and negative lymph node rate (46.2% versus 66.1%, *P* = 0.001) (ref. ^2^). Notably, a clear survival advantage of nCRT over nCT has not been established based on current evidence despite differential tumor responses^38^. In a post hoc analysis, we compared the efficacy of nAde followed by minimally invasive esophagectomy with historical standard-of-care nCT/nCRT data from the previously published CMISG1701 study. NATION-1907 indicated an OS benefit with an anti-PD-L1 antibody versus standard-of-care treatment in neoadjuvant settings. As a caveat, this conclusion was based on a historical comparison and should be interpreted with caution. Whereas nCT can ‘debulk’ tumors preoperatively, neoadjuvant immunotherapy aims to enhance systemic immunity against tumor antigens, eliminating micrometastatic tumor deposits that would otherwise be a source of postsurgical relapse^39^. This is in line with the findings in the adenocarcinoma-based PERFECT study in which responders with short-term neoadjuvant atezolizumab plus nCRT before surgery could derive long-term benefits from treatment in terms of OS and PFS compared with nCRT therapy for resectable advanced esophageal adenocarcinoma, although without statistical power^15^. Moreover, preliminary evidence revealed that 2-year OS in NATION-1907 was superior to neoadjuvant anti-PD-1 blockade plus chemotherapy in locally advanced ESCC in some small phase II trials^40–42^ (93% with nAde, 74%–87% with chemoimmunotherapy, *P* = 0.066) (Extended Data Fig. 8a,b), although the latter promoted more pathological regression preoperatively (pCR rate of 30%). Of note, paclitaxel may compromise the clinical outcomes of accompanying atezolizumab^43^ by impairing the expansion of responsive immune cells in the effective response to atezolizumab (anti-PD-L1). This raises a concern that the synergistic effects of anti-PD-(L)1 blockade and chemotherapy may be controversial in the neoadjuvant setting for locally advanced ESCCs and should be further explored in large cohorts for head-to-head comparison. Our NATION-1907 trial suggests that immunotherapy alone might be a promising therapeutic strategy in locally advanced ESCC.

A key result of the exploratory biomarker was the first-defined classification of a TME phenotype to stratify all MPR patients in response to neoadjuvant PD-L1 blockade, as well as patients with longer OS in the TCGA ESCC and nine pan-cancer immunotherapy cohorts. We classified all patients into IE, tumor-proliferation and fibroblast-enriched subtypes according to unique compositions of immune cells and stromal cells, along with the pathway activity of cancer hallmarks. Importantly, the IE subtype was correlated with the pathological response to neoadjuvant anti-PD-L1 blockade. The benefit of nAde was limited to IE tumors at baseline, most notably tumors with high PD-L1 expression (combined positive score (CPS) > 10). This finding is in line with Chen et al.^44^ who found that patients with type II PD-L1^+^/TILs^+^ TME had a better tumor response to anti-PD-(L)1 therapy and another study in which patients with a higher *IFN-*γ signature score at baseline had relatively longer OS^15^. Among non-IE tumors, recruited immune cells after nAde showed two immune ‘cold’ patterns, immune-excluded (PD-1^+^ CD8^+^ T cells) and immune-suppressive (M2-like macrophages), suggesting that M2 macrophages may restrict T cell migration into a tumor through long-lasting contact^24,45^. Specially, fibroblast-enriched tumors, which had a higher infiltration of fibroblasts, and activity of the *TGF-*β and EMT pathway failed to become the IE subtype during anti-PD-L1 blockade, indicating that combine simultaneous stromal signaling suppression (for example, anti-TGF-β antibody or anti-fibroblast) with immune checkpoint blockade may be a beneficial therapeutic strategy for ESCC patients with a fibrotic TME phenotype.

Blockade of the PD-1–PD-L1 axis was thought to primarily boost pre-existing tumor-specific T cell responses. We demonstrated that the clonotype diversity of pre-existing ITCs most likely to recognize tumor neoantigens was associated with favorable clinical benefits, and the clonotypic dynamics of pre-existing ITCs were hallmarks of responsive patients. Neoadjuvant adebrelimab induced clonal replacement of pre-existing T clones and new emergent T clonotypes, which was consistent with published results in the context of anti-PD-1 therapy^46^. By contrast to the limited reinvigoration capacity of pre-existing tumor-reactive T cells^26^, our results revealed marked expansion of pre-existing ITCs, along with robust infiltration of new T cell clonotypes. The two main sources of tumor-reactive T cells were speculated to make differential contributions in different cancers^27,47^. After neoadjuvant adebrelimab only a small proportion of novel T clonotypes existed in peripheral blood, suggesting newly recruited T cells came from either the peripheral blood or other sources such as lymphoid organs^48^. Although our bulk TCR-seq supports the first possibility for the source of tumor-reactive T cells induced by PD-L1 blockade, further work is required to identify the functional status–clonotype relationship of T cells using single-cell RNA-seq and TCR-seq. Our results shed light on the clonal T cell response to immune checkpoint blockade in ESCC, which has important implications for the design of immunotherapy to increase the clone size of existing tumor-reactive T cell clonotypes and/or recruitment of additional tumor-specific CD8^+^ T cells with replicative compacity^27^.

Our study is limited by its small sample size and single-center nonrandomized setup. Comparing patients in trials with real-world patients has several challenges owing to various factors, including differences in the study duration and the availability of other therapies during different periods. With a median follow-up of 27 months, further continuous follow-up should be carried out to fully evaluate clinical outcomes. However, this preliminary result is encouraging, and comparison with the previously published CMISG1701 study supports the importance of neoadjuvant PD-L1 blockade as a new therapeutic strategy, especially for patients intolerant of chemotherapy or chemoradiotherapy. An additional larger cohort and well-designed head-to-head comparison with nCRT or nCT that are beyond the scope of this study are planned.

These data are complementary to standard-of-care treatments in neoadjuvant settings. The translational study identified patients with favorable pathological responses to immunotherapy, supporting a promising alternative regimen to avoid overtreatment by chemotherapy and chemoradiotherapy with or without immunotherapy.

## Methods

### Patient and sample collection

Eligible patients were at least 18 years of age; had histologically confirmed stage cT2-4aN0-2M0 (*AJCC*, eighth edition) resectable ESCC, an Eastern Cooperative Oncology Group performance status of 0 or 1, at least one measurable/evaluable lesion according to Response Evaluation Criteria in Solid Tumors (RECIST), v.1.1, and adequate hematology, coagulation, liver, lung and renal function. Patients with nonsquamous cell, inoperable or metastatic ESCCs, who had been previously treated with ani-PD-(L)1 therapies, had another previous or current malignant disease, were potentially immunotherapy intolerant, harboring active brain or leptomeningeal metastasis or autoimmune disease were excluded. Postoperative management and follow-up are described in the Supplementary study protocol. Tumor samples at baseline and at the time of surgery were macroscopically reviewed by two experienced pathologists, collected within 30 min after esophagoscopy and surgery, and snap frozen in liquid nitrogen for subsequent multiomics analysis. Peripheral blood at baseline, on therapy and post-therapy were collected for TCR-seq.

### Study design and interventions

NATION-1907, a single-center, nonrandomized, phase 1b study, enrolled 30 patients with resectable ESCC from Zhongshan Hospital, Fudan University, between 26 December 2019 and 29 August 2020. Patients received adebrelimab (administered intravenously at a dose of 20 mg per kg body weight every three weeks) on day 1 of a planned 21-day cycle, and two doses before surgery. The study was approved by the Research Ethics Committee of Zhongshan Hospital and carried out in accordance with The International Conference on Harmonization Good Clinical Practice Guidelines, the principles of the Declaration of Helsinki, and national or local laws or regulations. Written informed consent was signed by all patients during enrollment.

Neoadjuvant adebrelimab was discontinued if one of the following occurred: informed consent was withdrawn because of a patient’s personal decision, or there was unacceptable toxicity, disease progression according to RECIST 1.1 criteria, or a patient had a life-threatening disease or condition preventing further treatment. Safety and efficacy data were reviewed by an independent data-monitoring committee. There was no bias toward age, gender or race in this clinical trial. The trial was open to men and women who met the inclusion and exclusion criteria outlined. The drug (adebrelimab) and medical examination were provided free to patients, but there was no additional participant compensation.

### Endpoints and response assessment and toxicity

The primary endpoints were safety and feasibility. Secondary endpoints were pCR, OS, RFS and R0 rates. Tumor assessment was carried out every 6 weeks, and the tumor response was evaluated according to RECIST 1.1 guidelines after blinded central review. Adverse events were assessed in all patients who had received at least two doses of the treatment; these events were graded according to the National Cancer Institute Common Terminology Criteria for Adverse Events, v.5.0.

### Immunohistochemical staining

PD-L1 expression analysis of formalin-fixed paraffin-embedded (FFPE) tumors at baseline was performed using PD-L1 immunohistochemistry 22C3 pharmDx assay (Agilent Technologies) on a Dako Autostainer Link 48 automated platform following an automated staining manual. CPS was defined as the number of PD-L1 positive cells (including tumor cells, macrophages and lymphocytes) divided by the total number of tumor cells, multiplied by 100.

### Multiplex immunofluorescent staining and analysis

FFPE tumor slides were analyzed against on three antibody panels. Panel 1: CD4 (clone EPR6855, dilution 1:100; Abcam, catalog no. Ab133616), CD8 (polyclones, dilution 1:200; Novus, catalog no. NBP2-34039), FOXP3 (clone 236A/E7, dilution 1:200; Abcam, catalog no. Ab20034), PD-L1 (clone EPR19759, dilution 1:200; Abcam, catalog no. Ab213524) and PD-1 (clone NAT105, dilution 1:200; Abcam, catalog no. Ab52587); panel 2: CD20 (clone L26, dilution 1:200; Abcam, catalog no. Ab9475), CD11c (clone EP347Y, dilution 1:500; Abcam, catalog no. Ab52632), CD68 (clone 968, dilution 1:400; Cell Signaling Technology, catalog no. 76437S), CD163 (clone EPR19518, dilution 1:100; Abcam, catalog no. Ab182422), IFN-γ (polyclones, dilution 1:200; Abcam, catalog no. Ab25101); panel 3: CD3 (clone SP7, dilution 1:200; Abcam, catalog no. Ab16669), Pan-CK (clone C-11, dilution 1:800; Abcam, catalog no. Ab7753), Vimentin (clone EPR3776, dilution 1:600; Abcam, catalog no. Ab92547), Ki-67 (clone SP6, dilution 1:100; Abcam, catalog no. Ab16667). Slides were counterstained with DAPI (dilution 1:1,000; Sigma) for nuclei visualization and subsequently coverslipped using Hardest mounting media (H-1400; VectaShield). Primary antibodies were incubated for 30 min in panels 1 and 2, and for 60 min in panel 3. All stained slides were imaged using the Polaris imaging system (Akoya Biosciences/PerkinElmer, Shanghai Kelin Institute) under the appropriate fluorescent filters for multispectral microscope. A whole slide was scanned and produced multispectral fluorescent images at ×200 magnification which were visualized in Phenochart v.1.1.0 viewer (Akoya Biosciences/PerkinElmer, Shanghai Kelin Institute).

### WES

Genomic DNA and total RNA from tumor tissues were simultaneously extracted using the QIAamp AllPrep DNA/RNA mini-Kit (Qiagen, catalog no. 80204) according to the manufacturer’s instructions, and included the steps of DNA/RNA separation, purification and collection in columns. Genomic DNA from peripheral blood samples was extracted using a QIAamp DNA mini-Kit (Qiagen, catalog no. 51304) according to the manufacturer’s protocol. The DNA concentration was quantified using a Qubit dsDNA BR Assay Kit (Thermo Fisher Scientific, catalog no. Q32850), and DNA integrity was evaluated by agarose gel electrophoresis. Whole-exome libraries were prepared using a MGIEasy Exome Universal Library Prep Set (MGI, catalog no. 1000009657) according to manufacturer’s instructions. Briefly, DNA was fragmented, adapter ligated, underwent probe hybridization and was subjected to PCR amplification. The well-prepared libraries were quality controlled using a Qubit dsDNA HS Assay Kit (Thermo Fisher Scientific, catalog no. Q32851) and Agilent DNA 1000 Kit (Agilent, catalog no. 5067-1504), and sequenced on a DNBSEQ T1 platform (MGI) with 100 bp paired-end reads. The mean depth of coverage was ×435 for tumor samples and ×212 for peripheral blood.

### Somatic variants calling

WES data were aligned to the hg38 reference genome using BWA (v.0.7.12). Duplicate reads were then removed by Picard (v.1.84). Local realignment and base quality score recalibration were carried out using GATK (v.4.1). Single nucleotide variants, small insertions and deletions were detected using SomaticSniper (v.1.0.5.1), MutTect2 (v.2.7.0), MuSE (v.1.0), Strelka (v.2.9.9) and Svaba (v.0.2.1), then annotated by ANNOVAR (v.180504). Single nucleotide variants were filtered to identify nonsynonymous exonic variants. Mutational signatures were determined using deconstructSigs (v.1.8.0) with default parameters applying COSMIC v.2 signatures as the reference with a maximum of two signatures. Copy number variants, tumor purity and ploidy were called using FACETS (v.0.16.0). Significantly amplified or deleted regions of copy number variants were identified by GISTIC (v.2.0.23).

### TMB and MSI evaluation

TMB was calculated using nonsynonymous mutations with a 33.86 Mb WES panel. MSI was examined by MSIsensor v.0.6 with default parameters.

### HLA genotyping and neoantigen prediction

Major histocompatibility complex (MHC) allele typing was performed using Polysolver (v.1.0). netMHC (v.4.0), netMHCpan (v.4.1), MHCflurry (v.2.0.4), MixMHCpred (v.2.1) and HLAthena (v.1.0) were then integrated to evaluate affinity between class I MHCs and somatic peptides. Candidate neoantigens with an MHC affinity <500 nM were further selected to estimate the neoantigen-specific TCRs using a transfer learning-based model (pMTnet v.1.0.0). the pMTnet output was a percentile rank representing the potential binding strength between the TCR and somatic peptides with a smaller rank indicating a stronger binding possibility. Further analysis was performed using the top 2% binding ITC–neoantigen pairs.

### Bulk RNA-seq

The concentration and integrity of total RNA were evaluated using a Qubit RNA HS Assay Kit (Thermo Fisher Scientific, catalog no. Q32852) and Agilent RNA 6000 pico kit (Agilent, catalog no. 5067-1513). RNA sample libraries were prepared using an MGI ribosomal RNA removal kit (MGI, catalog no. 1000005953) and MGIEasy RNA Library Prep Set (MGI, catalog no. 1000006383) in accordance with the manufacturer’s manual. The concentrations of the libraries were quantified using a Qubit dsDNA HS Assay Kit (Thermo Fisher Scientific, catalog no. Q32851) and the quality of the libraries was evaluated using the Agilent DNA 1000 Kit (Agilent, catalog no. 5067-1504). Libraries were sequenced on a MGISEQ-2000 sequencer (MGI) with 100 bp paired-end reads. First, adapters and low-quality sequences were filtered, then qualified reads were aligned to the GRCh38 reference genome using STAR v.2.5.1b. Picard Tools (v.1.84) was used to remove duplicated reads.

### Targeted immune genes RNA-seq

Total RNA was extracted from 5 μm FFPE slides from pretreatment tumors using RNeasy FFPE Kit (Qiagen, catalog no. 73504) and quantified by Nanodrop and Qsep-100 (Thermo Fisher Scientific). The qualified RNA samples were hybridized with all the probes in the panel (NanoString Technologies). Finally, the hybridized products were purified through nCounter Prep Station to remove excess capture and reporter probes and to immobilize transcript-specific ternary complexes on a streptavidin-coated cartridge. Purified samples were finally scanned by nCounter Digital Analyzer (NanoString Technologies), analyzed used nSolver analysis software (v.4.0.70) and the nSolver Advanced data analysis package (v.2.0.134).

### Differentially expressed genes and pathway enrichment analysis

Differentially expressed genes between well and poor responders were analyzed using Deseq2 v1.30 log_2_(fold change) >1 and adjusted *P* values <0.05 were considered the cutoff criteria for differentially expressed gene analysis. Pathway enrichment was analyzed by ClusterProfiler v.4.4.4 using differentially expressed genes, and Gene Set Enrichment Analysis (GSEA) tests were analyzed for their *q*-value, with gene signatures of cancer hallmarks obtained from Molecular Signatures Database v.7.4. Hallmark pathways were considered significant at Benjamin–Hochberg-adjusted *q*-values <0.05. The hallmark score was calculated using Gene Signatures Variation Analysis (v.1.46) with single-sample GSEA as an enrichment method. Gene signatures of significant pathways were used. Briefly, for each tumor sample and hallmark score, we obtained a score between [−2, 2], with extreme values close to 2 or −2, indicating the extent of enrichment of gene signatures. Volcano plots were generated by EnhancedVolcano (v.1.14.0).

### IFN/EMT signature

Sparse linear regression analysis (elastic net) was used in glmnet v.4.1.4 to capture features from the gene expression matrix across 18 samples. Genes were selected as candidate features if they belonged to EMT, *INF-*α or *INF-*γ gene sets and if there was at least a onefold difference between well and poor responders, resulting in a total of 134 candidate genes. The cost function of the elastic network regression algorithm combines lasso and ridge regression, which uses two parameters, λ and α, to control the size of penalty terms. We set the α value at 0.5 and the λ value at 0.24. Finally, pathway score was calculated for each sample using 12 selected genes with regression coefficients. Nine immunotherapy cohorts for patients treated with immunotherapy alone were used to verify whether the IFN/EMT signature could be widely applied in a variety of cancer types.

### Definition of cell scores and signature

The fractions of major cell types were calculated using CIBERSORTx v.1.0.4 and Ecotyper v.1.0 with RNA-seq expression profiles. ESTIMATE v.1.0.13 was used to calculate immune score and stromal scores using bulk RNA-seq data. To determine the correlation between the signature associated with inflamed TME and clinical benefits, TIL score was calculated as the sum of CD45^+^ T, CD8^+^ T, cytotoxic CD8^+^ T, exhausted CD8^+^ T, T helper 1, T_reg_ and B cells, using the nSolver Advanced data analysis package (v.2.0.134). The CD8 lineage signature was defined using average expression (measured by log_2_(fragments per kilobase of exon model per million mapped fragments + 1) of CD8 lineage markers (*CD2, CD3D, CD3E, CD8A* and *CD8B*). The exhausted signature was defined using the average expression of immune checkpoint markers (*CD274, HAVCR2, TNFRSF9, CTLA4* and *TOX*). The chemokine ligand signature was defined using the average expression of chemokine ligand genes (*GZMB, CXLC9, CXCL10* and *CCL5*). The HLA-II score, was defined as the mean expression of six *HLA-DR* genes (*HLA-DRA, HLA-DRB1, HLA-DRB2, HLA-DRB3, HLA-DRB4* and *HLA-DRB5*), four *HLA-DQ* genes (*HLA-DQA1, HLA-DQA2, HLA-DQB1* and *HLA-DQB2*) and two *HLA-DP* genes (*HLA-DPA1* and *HLA-DPB1*). The DC score was calculated based on the average expression of dendritic cell growth factor *FLT3* and dendritic cell markers (*CLEC9A* and *XCR1*). The immune cytolytic activity was measured as the geometric mean of *GZMA* and *PRF1* expression values.

### TME subtype identification and verification

Because we observed differential enrichment of hallmarks between well and poor responders, we further calculated the single-sample GSEA score of 13 cancer-associated hallmarks and performed half-supervised cluster analysis. To explore the correlation between inflamed TME-related signatures and clinical benefits, we defined ‘TME subtype’, and patients were clustered and labeled as IE, tumor-proliferation and fibroblast-enriched subtypes according to the half-supervised results of 13 cancer-associated hallmarks. To confirm the observed TME subtype pattern, we applied the TME subtype in the TCGA ESCC cohorts and several published pan-cancer immunotherapy datasets. Hierarchical clustering was then performed on these samples with heatmap v.1.0.12 using row-scaling, Euclidean distance and ward.D2 clustering.

### TCR-seq and assessment of the TCR repertoire

Multiplex PCR was performed to amplify the CDR3 regions of the rearranged TCR-β chain from genomic DNA. Thirty-two V-gene specific primers and 13 J-gene primers were used for multiplex PCR. The amplified products were cyclized into single-strand DNA libraries using an MGIeasy Circulation Kit, and then sequenced on the MGI2000 sequencer with 100 bp paired-end reads. VDJtools (v.1.2.1) was used for sequence alignment. TCR repertoire diversity was assessed by productive clonality, which was a measure of species diversity. CDR3 amino acid sequences that had stop or frameshift code, length <5 bp, or did start with ‘C’ or end with ‘F/W’ were considered nonproductive and excluded from subsequent clonotype analyses.

TCR richness was defined as the total number of unique clonotypes. A clonality value of 0 represented the most diverse repertoire (each T cell had a unique T cell clonotype), whereas a value of 1 represented a monoclonal T cell population.

### T cell fraction calculation

T cell fractions in tumor tissue and peripheral blood were estimated from WES data using the T cell exome TREC tool (T cell ExTRECT v.1.0.1) (ref. ^49^).

### Neoantigen-specific ITC prediction

Neoantigens were classified into immune clearance neoantigens and novel neoantigens according to the corresponding unique somatic mutations identified in pretreatment and post-treatment tumors. Neoantigens derived from somatic mutations that remained persistent during anti-PD-L1 treatment were defined as immune persistence neoantigens. ITCs specifically binding to the three types of neoantigen (neoantigen-specific ITCs) were inferred by pMTnet (v.1.0.0), which predicted the affinity between TCRs and peptide–MHC complex.

### Intratumoral T cells

ITCs were defined as T cells that shared the same clonotypes between pretreatment and post-treatment tumors. T cell clonal space was defined and calculated as the summed frequency of clones in each of the four respective groups relative to the total T cell repertoire. ITCs were ranked according to their frequency of 10^0^, 10^−1^, 10^−2^, 10^−3^, 10^−4^, 10^−5^, 10^−6^ in the resected tumor bed, and further divided into clonal expanded ITCs and clonal contracted ITCs based on the increase and decrease in the frequency of each clonotype during anti-PD-L1 therapy. New T clonotypes were defined as unique T cell clonotypes in the post-treatment tumors, suggesting a new infiltration of T cells.

The proportion of clonality and richness of ITCs between well and poor responders were compared by mean ± s.d. We further systematically evaluated T cell dynamics in peripheral blood from patients who had both tumor tissue and peripheral blood available at baseline, week 3 and week 6. Mean and s.e.m. were calculated at each time point. Circulating ITCs were defined as those that shared the same clonotypes between peripheral T cells and ITCs, implying that pre-existing ITCs also existed in peripheral blood. In addition, circulating ITCs were divided into circulating expanded and contracted ITCs; the former were defined as those that shared same clonotypes between peripheral TCRs and clonally expanded ITCs, suggesting that expanded ITCs also existed in peripheral blood. Circulating new T clonotypes were defined as those that shared the same clonotypes between peripheral TCRs and new T clonotypes in post-treatment tumors.

### Identification of differentially expanded/contracted clones

ITCs that had a significant increase in frequency after treatment compared with baseline were defined as differentially clonal expanded ITCs, whereas those with a significant decrease in frequency were defined as differentially clonal contracted ITCs. For differential frequency analysis of T clones between baseline and post-treatment tumors, Fisher’s exact test was used to determine differential expanded and contracted clones based on the clonotype count before and after therapy. *P* values <0.05 were considered statistically significant.

### Post hoc comparison with historical data

Our previously published cohort of 264 patients who received nCT or nCRT in the CMISG1701 trial were selected for post hoc comparative analysis^2^. We used the IPTW method^50^ to control the potential difference in baseline demographic and clinical characteristics and compare clinical efficacy between nAde and nCT/nCRT. We used the ipwpoint function in R package ipw (v.1.2) to estimate the inverse probability of treatment weights. Propensity scores were estimated using multinomial logistic regression with tumor site and clinical stage as covariates. To assess the balance, standardized mean differences in covariate values were compared across treatment groups in an IPTW sample. Propensity scores were fit iteratively by adding or deleting nonlinear terms and two-way interactions and checking balance statistics until an optimal balance was achieved. Following IPTW, sufficient balance based on a conservative cutoff of standardized mean difference <0.25 was achieved for both tumor site and clinical stage. Covariate-adjusted survival curves and cumulative incidence estimates were generated with Kaplan–Meier methods using IPTW. We also used IPTW to compare the difference in clinical outcome between neoadjuvant mono-immunotherapy and chemoimmunotherapy.

### Statistical analysis

Thirty patients were enrolled for this study. A Simon optimal two-stage design was used. Six patients were accrued to the first stage, and if five or more patients proceeded to surgery without extended treatment-related delays, 21 patients would be enrolled on the second stage. If more than 23 of the first 27 patients proceed to surgery without extended treatment-related delays, the primary efficacy endpoint would be met and this regimen would be considered worthy of further testing. This design allowed early study termination for excessive surgery delay. The probabilities of a type I error and type II error were set at 5% and 20%, respectively. Throughout the study, side effects, adverse events and feasibility were continuously monitored. Similar to a previous study^7^, we hypothesized that treatment would not be feasible if the probability that surgery would be delayed was ⪖90% for >25% of the patients. We also determined that the treatment was not safe if the probability of a risk of grade 3 or 4 toxic effects was ⪖70% for >25% of the patients.

All statistical analyses were carried out using R (v.4.1.1) and python (v.3.7.9). In all boxplots, the center line and box boundaries represent the median, 25th and 75th percentiles, upper and lower whiskers represent 75th percentiles +1.5× interquartile range and 25th percentiles −1.5× interquartile range, respectively, and points indicate outliers. A nonparametric two-sided Wilcoxon rank-sum test was used to compare two populations, unless they followed normal distributions, in which case a two-sided *t*-test was used. *P* values were adjusted for multiple comparison false discovery rate using the Benjamin–Hochberg procedure, and *P* < 0.05 was considered significant.

RFS was defined as the time from surgery to the date of progression/recurrence or death (if a patient died without progression/recurrence). OS was defined as the time from the start neoadjuvant therapy to last known vital status. Patients alive at the last follow-up date were censored. OS and RFS rates were calculated by the Kaplan–Meier method using survminer (v.0.4.9). The package gtsummary (v.1.6.2) was used to summarize 12- and 24-month survival probabilities. The group difference in OS and RFS between well and poor responders was evaluated using a two-sided log-rank test. HR and *P* values were calculated in survival (v.3.4). Cox proportional hazards regression model was applied to estimate the HR. The R packages purrr (v.0.3.5), plyr (v.1.8.8), tidyr (v.1.2.1), dplyr (v.1.0.10) and ggsignif (v.0.6.4) were used for data handling in R v.4.1.1. The R packages ggplot2 (v.3.4.0), cowplot (v.1.1.1), ggalluvial (v.0.12.3), RColorBrewer (v.1.1.3), ggrepel (v.0.9.2), ggthemes (v.4.2.4), ggpubr (v.0.5.0) were used for plotting.

### Reporting summary

Further information on research design is available in the Nature Portfolio Reporting Summary linked to this article.

## Online content

Any methods, additional references, Nature Portfolio reporting summaries, source data, extended data, supplementary information, acknowledgements, peer review information; details of author contributions and competing interests; and statements of data and code availability are available at 10.1038/s41591-023-02469-3.

### Supplementary information

Supplementary Information Supplementary Figs.1–8, Tables 1–7 and Study protocol.

Reporting Summary

Supplementary Tables Supplementary Tables 1–7.

### Source data

Source Data Fig. 1 Statistical source data.

Source Data Fig. 2 Statistical source data.

Source Data Fig. 3 Statistical source data.

Source Data Fig. 4 Statistical source data.

Source Data Fig. 5 Statistical source data.

Source Data Extended Data Fig. 2 Statistical source data.

Source Data Extended Data Fig. 3 Statistical source data.

Source Data Extended Data Fig. 4 Statistical source data.

Source Data Extended Data Fig. 5 Statistical source data.

Source Data Extended Data Fig. 6 Statistical source data.

Source Data Extended Data Fig. 7 Statistical source data.

Source Data Extended Data Fig. 8 Statistical source data.

## Figures and Tables

**Extended Data Table 1:**
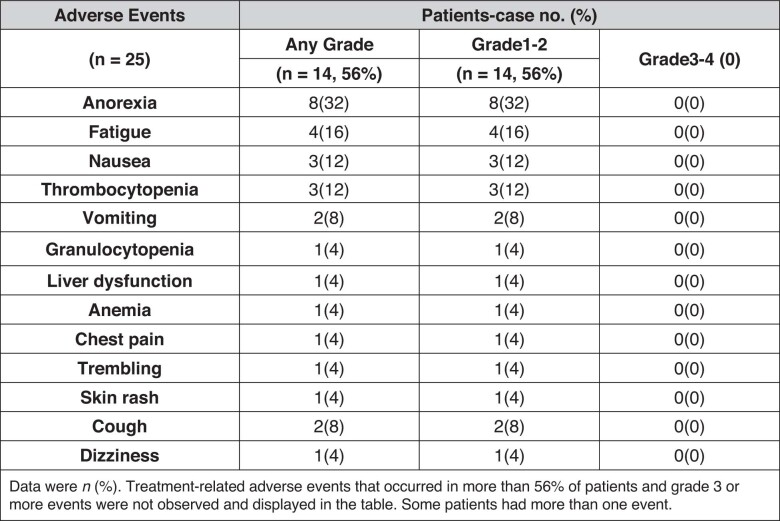
Treatment-related adverse event of neoadjuvant adebrelimab blockade

**Extended Data Table 2:**
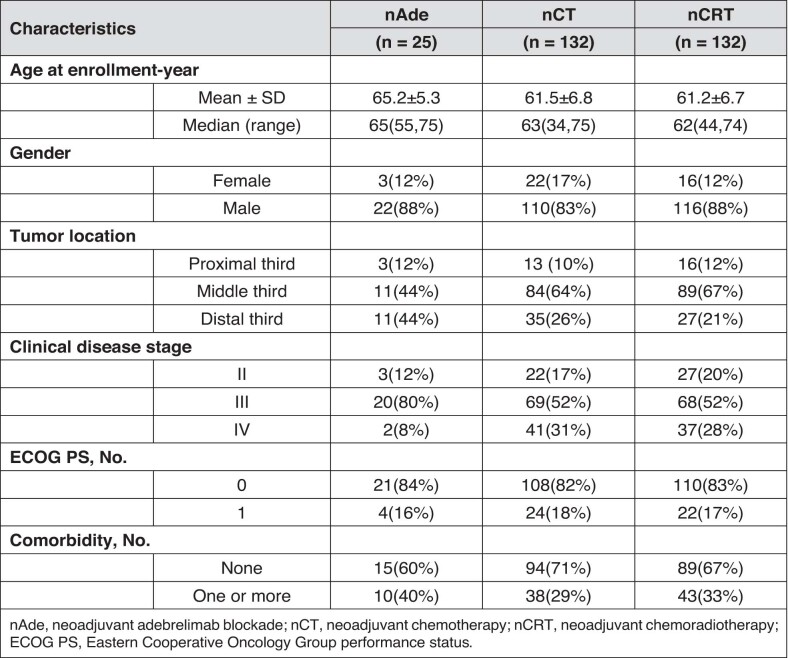
Patient characteristics between nAde and historical nCT or nCRT studies before IPTW adjustment

IPTW, inverse probability of treatment weighting.

## Data Availability

Deidentified raw sequencing data of participated patients were deposited in CNGB Nucleotide Sequence Archive (CNSA) with accession codes CNP0002585, CNP0003632, CNP0003659. Datasets of this clinical trial can be requested 12 months after this article is published. Researchers who request access to raw and analyzed data should send an email to the corresponding authors Q. Zhou and K. Wu to clarify the research purpose, and will be reviewed by the BGI Institutional Review Board, considering the risk of patient re-identification. Data are available for approved eligible applications and investigators, after signing a data access agreement. Source data are provided with this paper.

## Code availability

Custom code for data processing and analysis for WES, bulk RNA-seq and TCR data is available at https://github.com/yuanjingnan/ESCC-code.
