# Publisher Correction: Neoadjuvant adebrelimab in locally advanced resectable esophageal squamous cell carcinoma: a phase 1b trial

**DOI:** 10.1038/s41591-023-02511-4

**Published:** 2023-07-28

**Authors:** Jun Yin, Jingnan Yuan, Yunjin Li, Yong Fang, Ruoxi Wang, Heng Jiao, Han Tang, Shaoyuan Zhang, Siyun Lin, Feng Su, Jianmin Gu, Tian Jiang, Dong Lin, Zhiliang Huang, Chaoxiang Du, Kui Wu, Lijie Tan, Qing Zhou

**Affiliations:** 1 Department of Thoracic Surgery, Cancer Center, Zhongshan Hospital of Fudan University, Shanghai, China; 2 HIM-BGI Joint Lab, Hangzhou Institute of Medicine (HIM), Chinese Academy of Sciences, BGI-Hangzhou, Hangzhou, China; 3 Guangdong Provincial Key Laboratory of Human Disease Genomics, Shenzhen Key Laboratory of Genomics, BGI-Shenzhen, Shenzhen, China; 4 Zhejiang Cancer Hospital, Hangzhou Institute of Medicine (HIM), Chinese Academy of Sciences, Hangzhou, China; 5 Zhongshan Hospital (Xiamen), Fudan University, Xiamen, China

**Keywords:** Cancer immunotherapy, Cancer microenvironment

Correction to: *Nature Medicine* 10.1038/s41591-023-02469-3. Published online 24 July 2023.

In the version of this article initially published, the Abstract was missing the ClinicalTrials.gov identifier, NCT04215471, which has now been inserted in the HTML and PDF versions of the article.

