# Supplementary material for: Neoadjuvant adebrelimab in locally advanced resectable esophageal squamous cell carcinoma: a phase 1b trial
Source: Nat Med. 2023 Jul 24;29(8):2068–78. doi: 10.1038/s41591-023-02469-3 (PMC10427424; doi:10.1038/s41591-023-02469-3)
Supplement: Supplementary file 1 — Supplementary Figs.1–8, Tables 1–7 and Study protocol. [file 41591_2023_2469_MOESM1_ESM.pdf]

nature medicine

Article https://doi.org/10.1038/s41591-023-02469-3

Neoadjuvant adebrelimab in locally advanced resectable esophageal squamous cell carcinoma: a phase 1b trial

In the format provided by the authors and unedited

Supplementary information

Contents

1 Supplementary Figures……………………………………………………………….2

Supplementary Fig. 1 | Tumor length, diameter reduction between well and poor responders……………………………………………………………………………..2

Supplementary Fig. 2 | Mutational signature between well and poor responders……………………………………………………………….. ……....3

Supplementary Fig. 3 | Predicted mutation-associated neoantigen load was associated with TMB……………………………………………………………..4

Supplementary Fig. 4 | HLA-A loss of P20……………………………………5

Supplementary Fig. 5 | TILs calculated by nanostring data…………………….6

Supplementary Fig. 6 | Dynamic evolution of T cells following PD-L1 blockade ……………………………………………………………………………...7

Supplementary Fig. 7 | Clonotypic dynamics of ITCs during PD-L1 blockade..8

Supplementary Fig. 8 | Neoantigen and cognate TCR pairs of ITCs…………...9

2 Supplementary Study protocol and Statistical analysis plan…………………10

1 Supplementary Figures

Supplementary Fig. 1 | Tumor length, diameter reduction between well and poor responders

Tumor length and diameter reduction for two groups. For box plots, center line, box bound represented median, the 25th and 75th percentiles, and upper and lower whiskers represented 1.5× interquartile range from box bound, respectively. Data points were plotted as dots. A two-sided Wilcoxon rank-sum test was used.

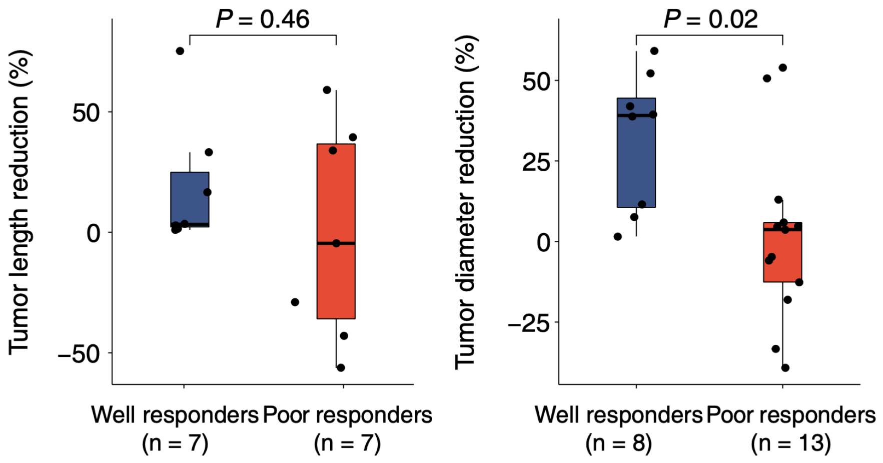

Supplementary Fig. 2 | Mutational signature between well and poor responders

Mutational signatures were displayed based on six types of substitutions (C>A, C>G, C>T, T>A, T>C and T>G). The mutation signatures were generated by incorporating the class of substitution as well as the bases immediately 5' and 3' to the mutated base. A slightly more prominent transversion-high signature (enriched in C>T single base substitutions, magenta bars) was seen in well responders compared with poor responders, however the differential enrichment did not reach statistical significance. Each mutational signature was displayed using a 96-substitution classification defined by the substitution class and the sequence context immediately 3' and 5' to the mutated base. The mutation types were shown on the horizontal axes and the vertical axes depicted the percentage of mutations attributed to a specific mutation type (blue: C>A, black: C>G, red: C>T, gray: T>A, green: T>C, beige: T>G). Well responders (n = 8), poor responders (n = 10).

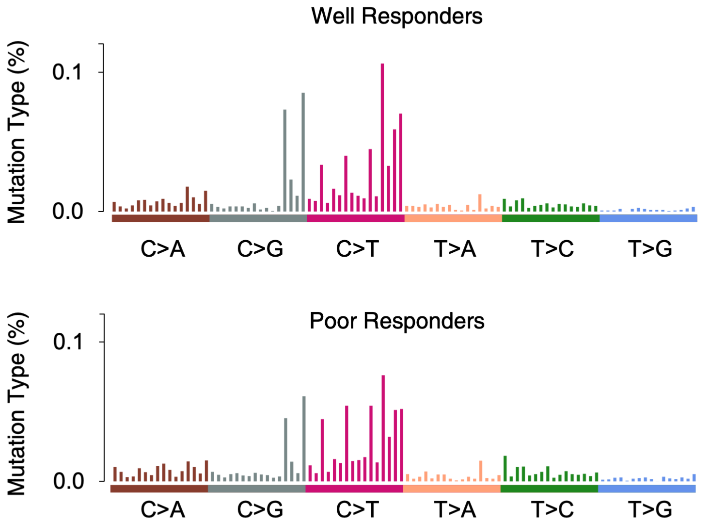

Supplementary Fig. 3 | Predicted mutation-associated neoantigen load was associated with TMB

Neoantigen candidates with predicted MHC affinity <500 nM was selected to estimate the neoantigen burden per tumor; the number of candidate neoantigens were found to be associated with TMB (Pearson R= 0.66, *P* = 0.005). Blue circles, well responders; red circles, poor responders. The gray area reflected the 95% confidence level interval for predictions of the linear regression model. *P* value from two-sided *t*-test was shown for statistical differences. TNB, Tumor neoantigen burden; TMB, Tumor mutation burden.

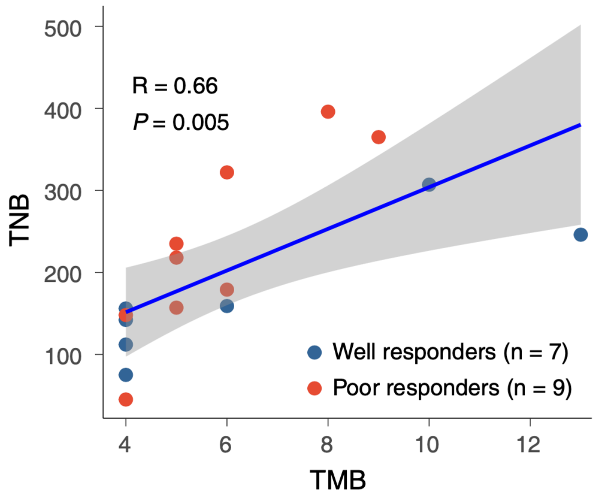

Supplementary Fig. 4 | HLA-A loss of P20

HLA LOH events and corresponding copy number status for tumors with HLA LOH in P20 before (**a**) and after (**b**) treatment. **c,** Heatmap showed a significant low expression of HLA complex from P20. Purple indicated high expression and green indicated low expression.

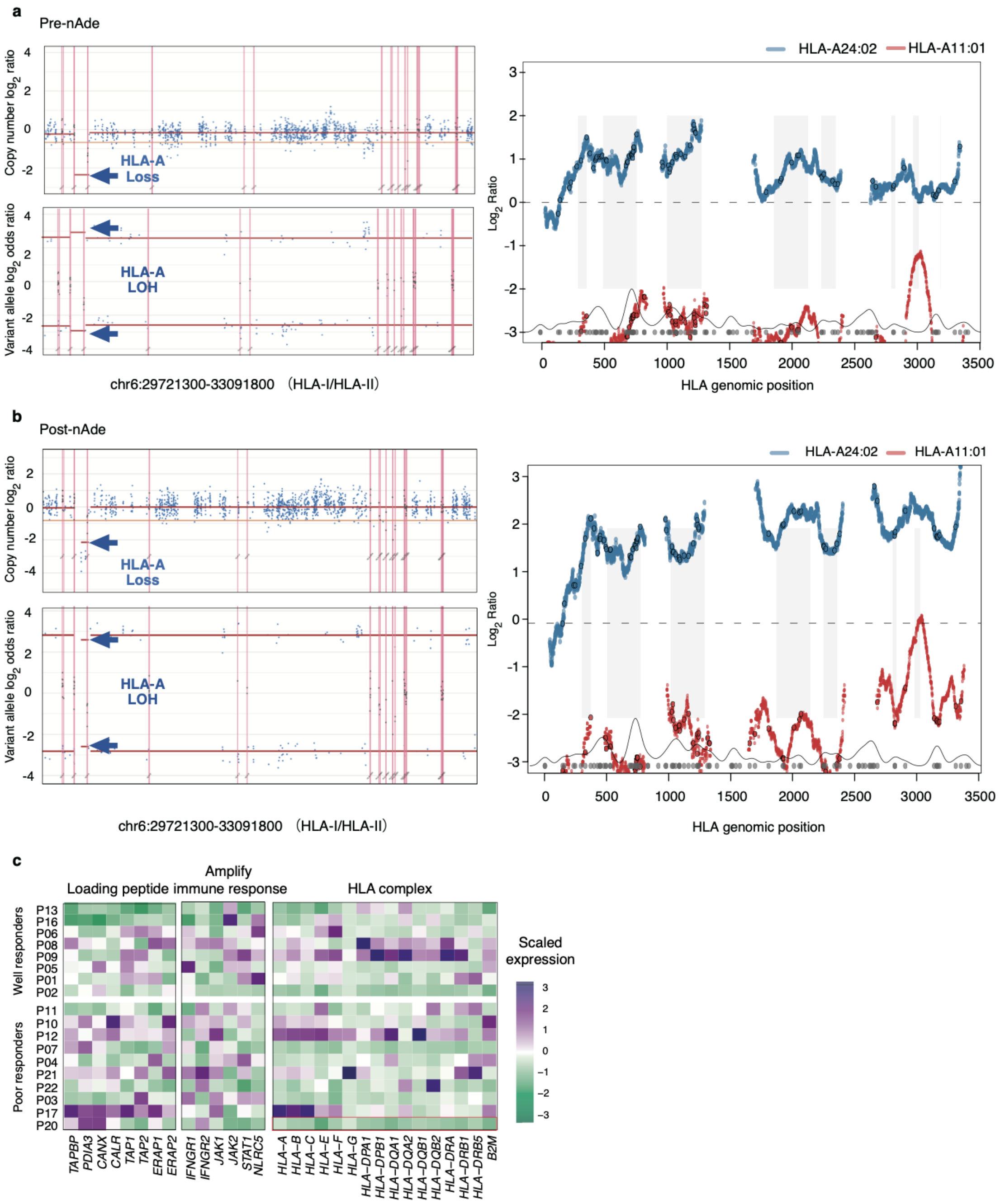

Supplementary Fig. 5 | TILs calculated by nanostring data

Unsupervised clustering of 13 cell type scores estimated by nanostring data for patients (n = 23) was shown, and the scores indicated relative proportions of immune cells in tissue. Response group was annotated. Red and Blue were used to indicate high and low expression.

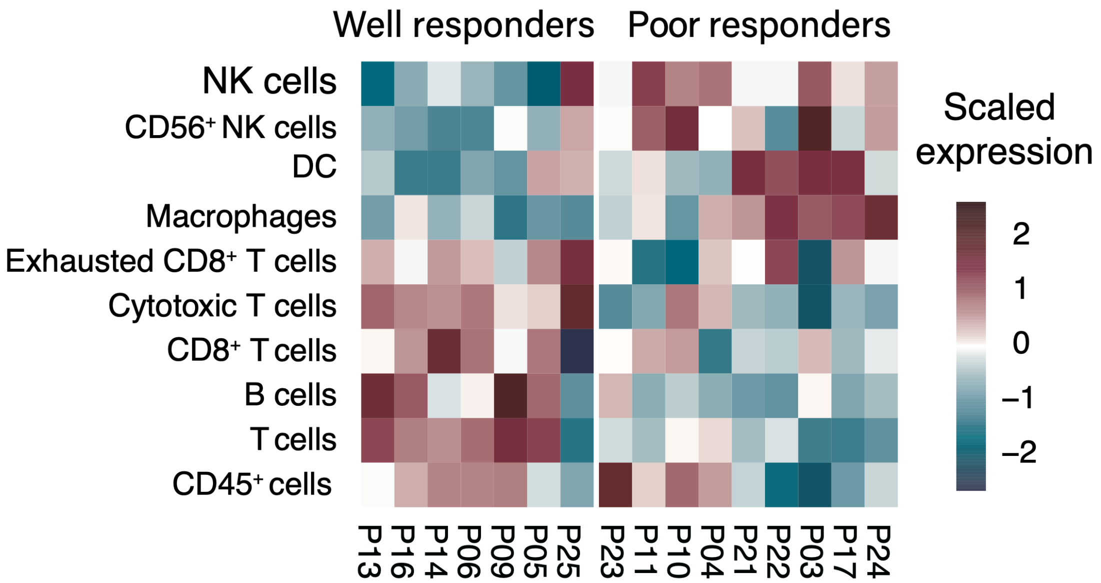

Supplementary Fig. 6 | Dynamic evolution of T cells following PD-L1 blockade

Sankey diagrams showed patterns of clonal replacement of ITCs (left panel) and dynamics of circulating ITCs (right panel) among different patients (n = 17). Colors indicated the clonally expanded ITCs (green), clonally contracted ITCs (red) and novel T cell (blue) clonotypes. The height indicated the productive frequency of the TCR repertoire, and clones connected between timepoints indicated that it was detected in both the pretreatment and posttreatment tumor.

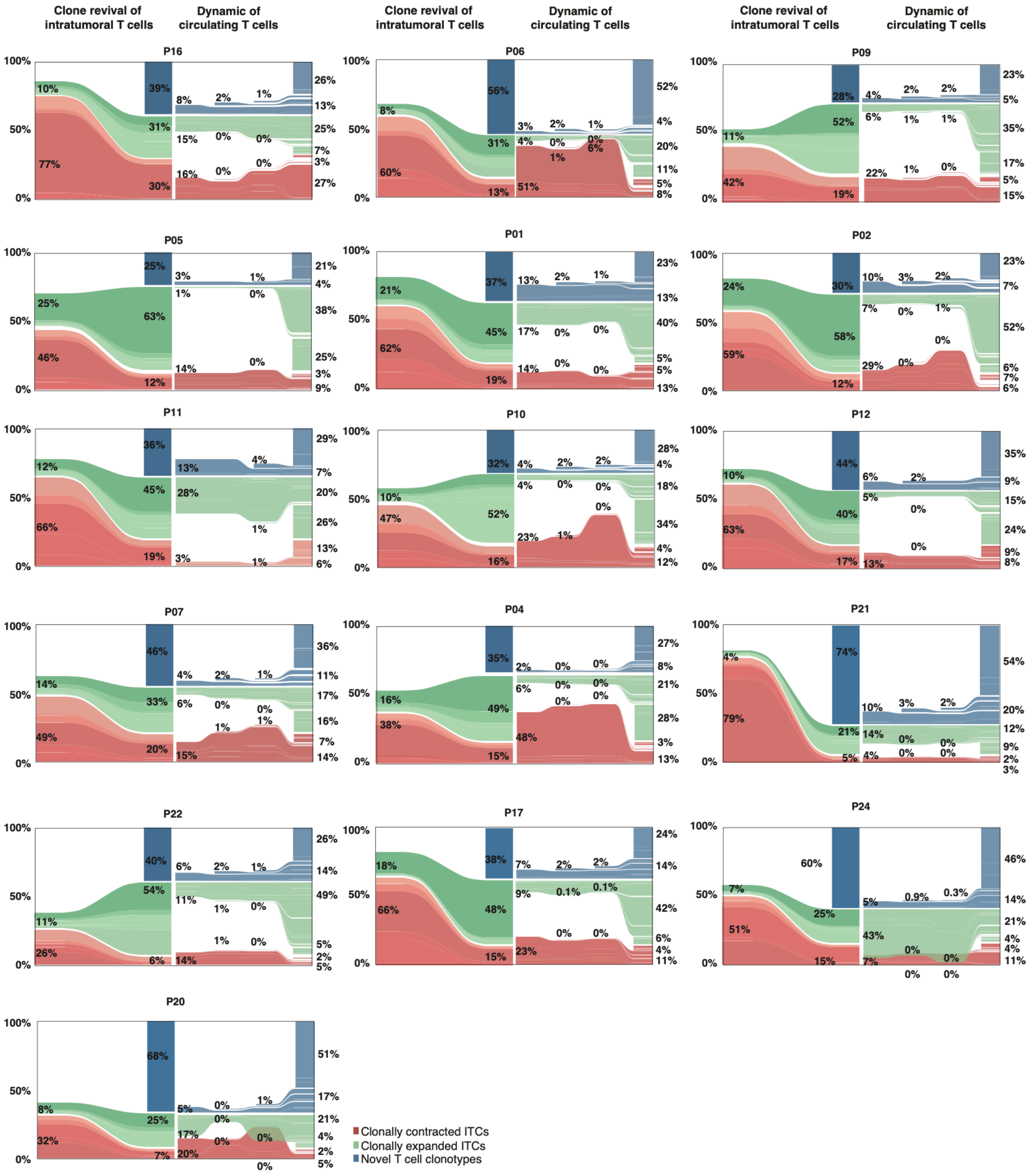

Supplementary Fig. 7 | Clonotypic dynamics of ITCs during PD-L1 blockade

Differential abundance analyses of 17 patients, with available tumor samples before and after treatment, represented TCR clonotypic expansions (labeled as significant positive; green) and contractions (labeled as significant negative; red). Fold change of intratumoral TCR clones was plotted on the x axis (log2 scale), and Fisher exact test *P* values was shown on the y axis (log scale) on the volcano plot. All *P* values were two-sided.

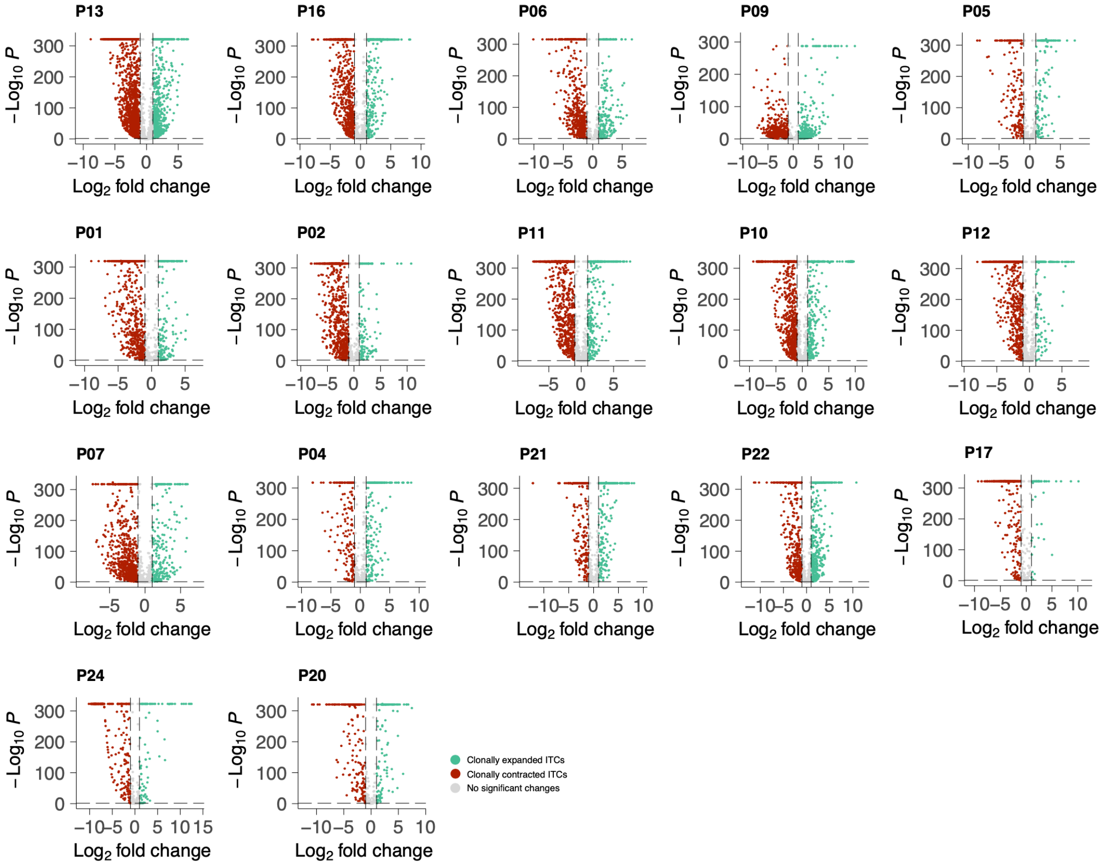

Supplementary Fig. 8 | Neoantigen and cognated TCR pairs of ITCs

Sankey diagram illustrated the relative flow between ITC and number of ITC-neoantigen pairs, interaction ITC and neoantigen, number of ITC-neoantigen pairs and neoantigen across 17 patients with ITCs. Two ITC clusters and three neoantigen clusters were defined and were vertically color coded, with expansion and contraction for ITC and immune clearance (neoantigens disappeared after nAde), immune tolerance (neoantigens kept persistent during nAde) and newly developed neoantigen (newly discovered neoantigens after nAde).

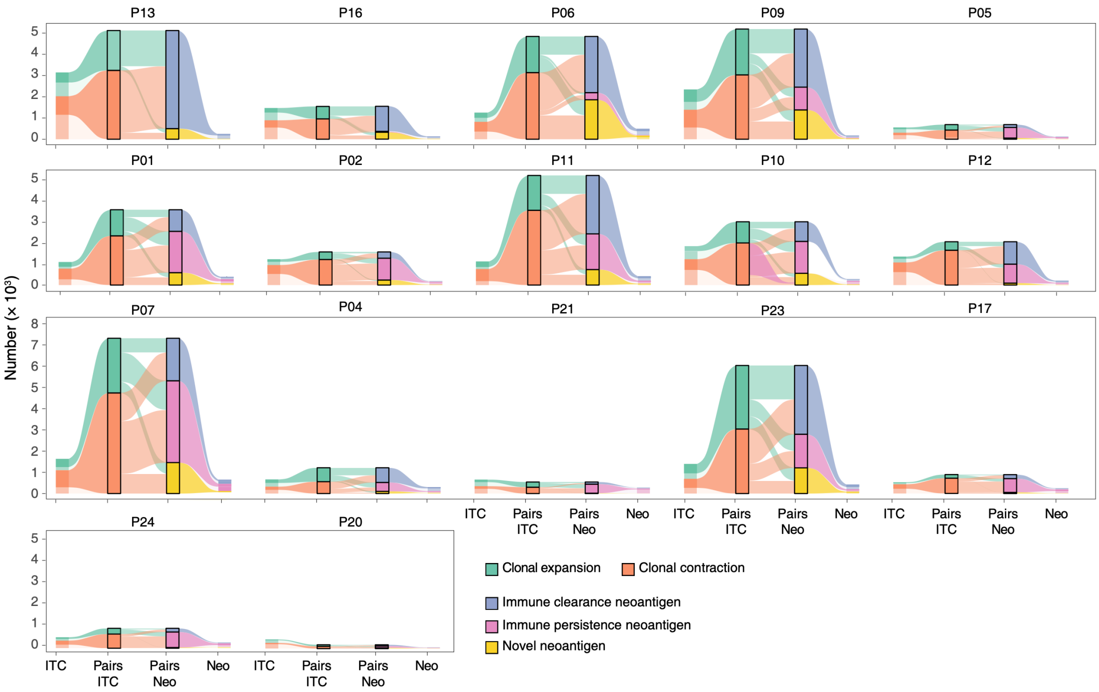

2 Supplementary Study protocol and Statistical analysis plan

Trial title: **N**eo**A**djuvant **T**herapy with **I**mmunoreagent (Adebrelimab, SHR-1316) for resectable es**O**phageal squamous cell carci**N**oma (NATION-1907)

Study protocol and statistical analysis plan

Sponsor:
Department of Thoracic Surgery, Cancer Center,
Zhongshan Hospital of Fudan University,
180 Fenglin Street,
Shanghai, 200032
China

Protocol version number:
NATION-1907 v1.0, dated 21, July 2019
NATION-1907 v1.1, dated 17, September 2019
NATION-1907 v1.3, dated 30, October 2019

CONFIDENTIALITY STATEMENT
The information is provided to you in confidence to enable you to perform the work. Do not give this document or any copy of it or reveal any proprietary information contained in it to any third party or person without the prior permission of an authorization.

Protocol ID number	B2019-205R
Test products	Adebrelimab(SHR-1316)
Principal Investigator	Lijie Tan, MD<br>Department of Thoracic Surgery, Cancer Center, Zhongshan Hospital of Fudan University, 180 Fenglin Street, Shanghai, 200032 China<br>Phone: (+86) 21 6404-1990-2917<br>Fax: 86-21-64038477<br>Email: tan.lijie@zs-hospital.sh.cn
Co-Investigators	Jun Yin, MD<br>Department of Thoracic Surgery,<br>Zhongshan Hospital of Fudan University<br>Email: jun_yin@fudan.edu.cn
	Yong Fang, MD<br>Department of Thoracic Surgery,<br>Zhongshan Hospital of Fudan University<br>Email: fang.yong1@zs-hospital.sh.cn
	Heng Jiao, MD<br>Department of Thoracic Surgery,<br>Zhongshan Hospital of Fudan University<br>Email: jiao.heng@zs-hospital.sh.cn
Statisticians	Fei Liang, MD<br>Department of Thoracic Surgery,<br>Zhongshan Hospital of Fudan University<br>Email: liang.fei@zs-hospital.sh.cn
Sponsor	Zhongshan Hospital of Fudan University
Support provided by	Hengrui Pharmaceuticals, China
Protocol version	NATION-1907 v1.3 dated 30 October 2019

Signature Page

Title: **N**eo**A**djuvant **T**herapy with **I**mmunoreagent (Adebrelimab, SHR-1316) for resectable es**O**phageal squamous cell carci**N**oma (NATION-1907)
Protocol ID no.: NATION-1907(B2019-205R)

I will conscientiously perform my duties as an investigator in accordance with the ICH-GCP regulations, and personally participate in or directly direct this clinical study. I have received the Investigator's Brochure for the investigational product for this clinical trial; I have known and read the preclinical research of the investigational drug and the study protocol of this clinical trial. I agree to perform my duties in accordance with the ICH-GCP, the Declaration of Helsinki, any applicable laws and regulations, and this study protocol. I will be responsible for making clinically relevant medical decisions, ensuring that subjects can receive appropriate treatment in time when adverse events occur during the study, and recording and reporting these adverse events in accordance with relevant national regulations. I promise to record the data in a true, accurate, complete and timely manner. I will accept the inspection of the drug supervision and management department to ensure the quality of clinical trials. I promise to keep the subject's personal information and related matters confidential. I agree to disclose to the Sponsor my full name, occupation, and, upon request, my expenditures related to the clinical study. I agree that the results of the study should be published. I will provide a resume of the principal investigator before the start of the study, submit it to the ethics committee, and submit it to the drug supervision and administration department for record.

Name	Title	Role	Signature	Date
Lijie Tan	Professor, MD, PhD, head of Department	Principal Investigator		
Jun Yin	Professor, MD, PhD, vice director of Department	Co-Investigator		

TABLE OF CONTENTS

Contact details…………………………………………………………………… 16
CONTENTS…………………………………………………………………………..…18

1 SUMMARY 19

2 STUDY DESIGN SCHEMA 23

2.1 STUDY DESIGN...23
2.2 STUDY ASSESSMENTS 24
2.3 TREATMENT ASSIGNMENT...24

3 OBJECTIVES 25

3.1 PRIMARY OBJECTIVES 25
3.1.1 TO ASSESS THE EFFICACY OF NEOADJUVANT ADEBRELIMAB FOR LOCALLY ADVANCED RESECTABLE ESCC [CLINICAL STAGE, T2-4AN0-2M0], AS MEASURED BY FEASIBILITY, BASED ON PATIENTS PROCEEDING TO SURGERY WITHOUT EXTENDED TREATMENT RELATED DELAYS. 25
3.1.2 TO ASSESS THE SAFETY OF NEOADJUVANT ADEBRELIMAB IN SAFETY RUN-IN PHASE, AS MEASURED BY DLT, BASED GRADE 3-4 TOXICITIES INCLUDING LIVER, GI, RENAL, PNEUMONITIS AND ANY OTHER GRADE 3-4 TOXICITY. 25
3.2 SECONDARY OBJECTIVES...25
3.2.1 TO ASSESS THE PATHOLOGICAL COMPLETE RESPONSE (PCR) RATE TO NEOADJUVANT ADEBRELIMAB IN RESECTED TUMOR AND LYMPH NODES... 25
3.2.2 TO ASSESS RECURRENCE-FREE SURVIVAL IN PATIENTS RECEIVING NEOADJUVANT ADEBRELIMAB IN THIS STUDY... 26
3.2.3 TO ASSESS OVERALL SURVIVAL IN PATIENTS RECEIVING NEOADJUVANT ADEBRELIMAB IN THIS STUDY. 26
3.2.4 TO ASSESS THE R0 RATE. 26
3.3 EXPLORATORY RESEARCH..27
3.3.1 TO INVESTIGATE DIFFERENCES IN GENETIC PROFILE (TMB, MSI) AS WELL AS EXPRESSION OF SELECTED IMMUNE MARKERS IN PATIENTS WHO WILL RESPONSE TO ADEBRELIMAB; TO DETERMINE DIFFERENCES IN THE QUALITY AND QUANTITY OF TUMOR INFILTRATING LYMPHOCYTES. 27
3.3.2 TO DETERMINE DIFFERENTIAL REGULATIONS OF IMMUNE CHECKPOINT PATHWAYS AND TUMOR IMMUNE MICROENVIRONMENT PHENOTYPES IN PATIENTS WHO WILL RESPONSE TO ADEBRELIMAB. 27
3.3.3 TO DETERMINE THE EFFECT OF NEOADJUVANT IMMUNOTHERAPY ON THE LOCAL AND SYSTEMIC IMMUNE STATUS OF ESCC. 27
3.3.4 TO EXPLORE THE ASSOCIATION BETWEEN ADEBRELIMAB EXPOSURE AND SELECTED PHARMACO-DYNAMIC MARKERS IN THE PERIPHERAL BLOOD AND IN THE TUMOR MICROENVIRONMENT, INCLUDING MEASUREMENT OF T CELL RECEPTOR (TCR) CLONOTYPES... 27

4 STUDY ENDPOINTS ASSESSMENTS 27

4.1 EFFICACY ENDPOINT 27
4.1.1 PRIMARY EFFICACY ENDPOINT 27
4.2 SECONDARY EFFICACY ENDPOINT 27
4.2.1 OVERALL SURVIVAL (OS) 27
4.2.2 RECURRENCE-FREE SURVIVAL (RFS) 28
4.2.3 PATHOLOGICAL COMPLETE RESPONSE RATE (PCR) 28
4.2.4 R0 RESECTION RATE 28
4.3 SAFETY ENDPOINT 28
4.3.1 PRIMARY SAFETY ENDPOINT 28

5 BACKGROUND AND RATIONALE 29

5.1 INTRODUCTION 29
5.2 PROGRAMMED DEATH-1 – MOLECULAR BIOLOGY 31
5.3 PROGRAMMED DEATH LIGAND -1 – EXPRESSION IN HUMANS 31
5.4 RATIONALE FOR NEOADJUVANT IMMUNOTHERAPY 32
5.5 DEVELOPMENT OF ADEBRELIMAB 34
5.6 CLINICAL EXPERIENCE WITH ADEBRELIMAB 34
5.7 RATIONALE FOR NEOADJUVANT ADEBRELIMAB IN ESCC, DOSING AND SCHEDULE AND INCLUSION/EXCLUSION CRITERIA 36
5.7.1 RATIONALE FOR NEOADJUVANT IMMUNOTHERAPY IN ESCC 36
5.7.2 RATIONALE FOR DOSING AND SCHEDULE 37
5.7.3 RATIONALE FOR MAIN INCLUSION AND EXCLUSION CRITERIA 38

6 PATIENT POPULATION 38

6.1 INCLUSION CRITERIA 38
6.1.1 MEN AND WOMEN AGE ≥ 18 YEARS, ≤ 75 YEARS. 38
6.1.2 HISTOLOGICALLY PROVEN ESCC (CORE BIOPSY REQUIRED). 38
6.1.3 STAGE 39
6.1.4 ADEQUATE ORGAN FUNCTION AS FOLLOWS 39
6.1.5 THE PATIENTS SHOULD BE ABLE TO UNDERSTAND OUR RESEARCH AND SIGN THE INFORMED CONSENT. 40
6.2 EXCLUSION CRITERIA 40
6.2.1 CANCER-RELATED CRITERIA 40
6.2.2 OTHER EXCLUSION CRITERIA 41
6.3 WITHDRAW CRITERIA 42

7 STANDARD OPERATION PROCEDURES (SOP) 42

7.1 CASE SELECTION 44

7.1.1 ASSESSMENT ... 44
7.1.2 SELECTION APPLICATION ... 44
7.1.3 ELIGIBILITY CONSULTING ... 44
7.1.4 ATTENTIONS ... 45
7.2 WRITTEN INFORMED CONSENT ... 45
7.3 NEOADJUVANT IMMUNOTHERAPY ... 45
7.4 ASSESSMENTS DURING NEOADJUVANT THERAPY ... 46
7.5 SURGERY PROCEDURE ... 46
7.5.1 SURGERY ... 46
7.5.2 OBSERVATIONS DURING OPERATION ... 46
7.6 POSTOPERATIVE MANAGEMENT ... 47
7.6.1 FLUID INFUSION AND NUTRITIONAL SUPPORT ... 47
7.6.2 REHABILITATION MANAGEMENT ... 47
7.6.3 DISCHARGE STANDARD ... 47
7.6.4 POSTOPERATIVE OBSERVATION ITEMS ... 47
7.7 FOLLOW-UP ... 49
7.7.1 FOLLOW-UP PERIOD AND ATTENTIONS ... 49
7.7.2 EXAMINATION ITEMS ... 49
7.8 ADJUVANT THERAPY ... 50
7.9 ASSESSMENTS DURING THE FOLLOW-UP ... 50
7.10 DEFINITIONS ... 50
7.10.1 ECOG PERFORMANCE STATUS ... 50
7.10.2 ASA CLASSIFICATION ... 51

8 EXPLORATORY IMMUNOLOGIC STUDIES ... 51

8.1 COHORT ... 51
8.2 TUMOR TISSUE SAMPLES ... 52
8.2.1 COLLECTION OF BIOPSY AND SURGICALLY RESECTED TUMOR TISSUE ... 52
8.2.2 TUMOR TISSUE HANDLING, TRANSPORTATION, STORAGE, AND PROCESSING ... 52
8.3 BLOOD SAMPLES ... 52
8.3.1 COLLECTION SCHEDULE ... 52
8.3.2 SPECIMEN HANDLING, TRANSPORTATION, STORAGE, PROCESSING ... 52
8.4 METHODS OF ANALYSIS ... 52
8.4.1 IMMUNOHISTOCHEMISTRY ... 52
8.4.2 MULTIPLEX IMMUNOFLUORESCENT STAINING ... 52
8.4.3 WHOLE EXOME SEQUENCING ... 53
8.4.4 BULK RNA SEQUENCING ... 53
8.4.5 TCR SEQUENCING ... 54

9 ADVERSE EVENTS ... 54

9.1 GENERAL ... 54
9.2 DEFINITIONS ... 54

9.2.1 ADVERSE EVENT (AE) 54
9.2.2 SERIOUS ADVERSE EVENT (SAE) 55
9.2.3 UNEXPECTED ADVERSE EVENT 55
9.2.4 EXPECTED (KNOWN) ADVERSE EVENT 56
9.2.5 RELATIONSHIP 56
9.3 SERIOUS ADVERSE EVENT COLLECTION AND REPORTING 56
9.4 NON-SERIOUS ADVERSE EVENTS AND REPORTING 57
9.4.1 LABORATORY TEST ABNORMALITIES 57
9.5 PREGNANCY 58
9.6 OVERDOSE 58
9.7 OTHER SAFETY CONSIDERATIONS 58

10 DATA MANAGEMENT 58

10.1 CASE REPORT FORM (CRF) 59
10.1.1 TYPES AND SUBMISSION DEADLINE 59
10.1.2 TRANSMISSION METHODS 59
10.1.3 AMENDMENT 59
10.2 MEETINGS 59
10.3 MONITORING 60
10.3.1 MONITORING ITEMS 60
10.3.2 ACCEPTABLE RANGE OF ADVERSE EVENTS 61
10.3.3 DEVIATION/VIOLATION OF STUDY PROTOCOL 61

11 PROVISIONS OF ADVERSE EVENTS 62

11.1 EVALUATION 62
11.2 REPORTING 63
11.2.1 **ADVERSE EVENTS WITH REPORTING OBLIGATIONS** 63
11.2.2 REPORTING PROCEDURE 64
11.3 RESPONSIBILITIES AND OBLIGATIONS 65
11.3.1 JUDGMENT OF STUDY DISCONTINUATION AND NECESSITY FOR SENDING AN EMERGENCY NOTICE TO THE HOSPITAL 65
11.3.2 REPORT TO PI EFFICACY AND SAFETY EVALUATION COMMITTEE 65
11.3.3 DISCUSSION OF ADVERSE EVENTS UNDER REGULARLY MONITORING 65
11.4 REVIEW OF EFFICACY AND SAFETY EVALUATION COMMITTEE 66

12 ETHICAL CONSIDERATIONS 66

12.1 RESPONSIBILITIES OF INVESTIGATORS 66
12.2 INFORMATION AND INFORMED CONSENT OF SUBJECTS 66
12.3 IDENTITY AND PRIVACY OF SUBJECTS 67
12.4 INDEPENDENT ETHICS COMMITTEE OR INSTITUTIONAL REVIEW COMMITTEE 67

12.5 SUPERVISORY AUTHORITY 68

13 ORGANIZATIONS AND RESPONSIBILITIES OF STUDY 68

13.1 RESEARCH COMMITTEE 68
13.2 EFFICACY AND SAFETY EVALUATION COMMITTEE 69
13.3 DATA CENTER 69
13.4 DATA AND SAFETY MONITORING BOARD (DSMB) 69
13.5 INDEPENDENT ETHICS COMMITTEE/INSTITUTIONAL REVIEW BOARD (IEC/IRB) 69
13.6 PRINCIPAL INVESTIGATOR RESPONSIBILITIES 69

14 GENERAL AND STATISTICAL CONSIDERATIONS 70

14.1 ANALYSIS SETS 70
14.1.1 MODIFIED INTENTION-TO-TREAT SET 70
14.1.2 SAFETY ANALYSIS SET 70
14.2 GENERAL CONSIDERATIONS 70
14.2.1 REFERENCE START DATE, END DATE AND STUDY DAY 70
14.2.2 BASELINE 70
14.2.3 ON-TREATMENT PHASE 70
14.2.4 DEFINITION AND USE OF VISIT WINDOWS 70
14.2.5 REPEATED OR UNSCHEDULED ASSESSMENTS OF SAFETY PARAMETERS 71
14.3 STATISTICAL CONSIDERATIONS 71
14.3.1 MISSING DATE OR INCOMPLETE DATE 71
14.3.2 CHARACTER VALUES OF CLINICAL LABORATORY TESTS 73
14.3.3 COMPUTING METHODS AND REPORTING CONVENTIONS 73
14.3.4 SUBGROUPS 74

15 STATISTICAL ANALYSIS 74

15.1 SUMMARY OF STUDY DATA 74
15.1.1 SUBJECT DISPOSITION 74
15.1.2 PROTOCOL DEVIATIONS 74
15.1.3 DEMOGRAPHIC AND BASELINE CHARACTERISTICS 74
15.1.4 TUMOR DIAGNOSIS AND TREATMENT HISTORY 75
15.1.5 TUMOR TREATMENT HISTORY 75
15.1.6 MEDICAL HISTORY 76
15.1.7 CONCOMITANT MEDICATIONS 76
15.2 EFFICACY ANALYSES 76
15.2.1 ANALYSIS OF PRIMARY EFFICACY ENDPOINTS 76
15.2.2 ANALYSIS OF SECONDARY EFFICACY ENDPOINTS 77
15.3 SAFETY ANALYSES 78
15.3.1 DOSING AND EXTENT OF EXPOSURE 78

15.3.2 ADVERSE EVENTS 78
15.3.3 CLINICAL LABORATORY EVALUATIONS 80
15.3.4 VITAL SIGN 81
15.3.5 ECG AND ECHOCARDIOGRAM 81
15.3.6 EASTERN COOPERATIVE ONCOLOGY GROUP (ECOG) PS 81

16 SAMPLE SIZE DETERMINATION 82

17 APPENDIX 83

17.1 LIST OF ABBREVIATIONS AND DEFINITIONS OF TERMS 83
17.2 TNM STAGING SYSTEM FOR ESOPHAGEAL CANCER (8TH EDITION) 84
17.2.1 DEFINITIONS FOR T, N, M 84
17.2.2 AJCC PROGNOSTIC STAGE GROUPS (SQUAMOUS CELL CARCINOMA) 84
17.3 ECOG PERFORMANCE STATUS SCALE 85
17.4 TIME POINT RESPONSE: PATIENTS WITH TARGET (+/- NON-TARGET) DISEASE 85
17.5 TIME POINT RESPONSE: PATIENTS WITH NON-TARGET DISEASE ONLY 85
17.6 INVESTIGATOR'S BROCHURE 85

18 REFERENCE 86

1 Summary

Investigational Products, Dose, Administration, Duration Of Treatment	This is a prospective single arm, non-randomized trial. Adebrelimab (SHR-1316) monotherapy administered intravenously over 30 minutes at 20 mg/kg every 3 weeks for two doses (administered Day 1 and Day 22) prior to surgical excision.
Research Hypothesis	Neoadjuvant immunotherapy may reduce micrometastatic niche, and prevent distant relapse, thereby prolonging recurrence-free survival and overall survival.
Objectives	Primary:<br>• Feasibility and safety<br><br>Secondary:<br>• Pathological complete response (pCR) rate<br>• Overall survival (OS)<br>• Recurrence-free survival (RFS)<br>• R0 resection rate<br><br>Exploratory endpoints:<br>• Identification of immunologic and genomic markers correlated with clinical and pathological response and resistance.
Study Design	This is a Phase 1b, non-randomized, non-comparative study of neoadjuvant adebrelimab monotherapy (adebrelimab 20 mg/kg, intravenously every 3 weeks, totally two cycles at Day 1 and Day 22) followed by minimally invasive esophagectomy in treatment naïve adult (18~75 years old) subjects with clinical stage T2-4aN0-2M0, resectable, locally advanced esophageal squamous cell carcinoma (ESCC). Patients with subsequent adjuvant therapy including chemotherapy, chemoradiotherapy or anti-PD-1 immunotherapy will be acceptable.

	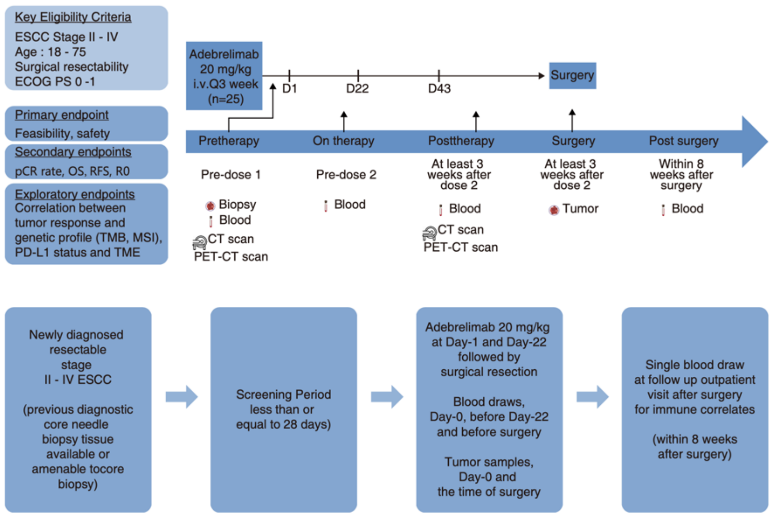<br><br>• Tissue samples at baseline will be collected for PD-L1 staining (IHC, 22C3).<br>• Tissue samples at baseline and surgery time will be collected for genetic profiling, transcriptomic analysis and intratumoral T cell diversity analysis.<br>• Peripheral blood at baseline, on-treatment, posttreatment will be collected to identify dynamic changes of T cells during neoadjuvant adebrelimab blockade.
Study Population	Key inclusion criteria:<br>• ECOG PS 0 or 1.<br>• Histologically confirmed resectable stage II to IV ESCC, as Eighth AJCC staging system.<br>• Patients must have at least one tumor amenable to serial biopsy in clinic or be willing to undergo serial biopsies through image-guided procedures. Baseline, on-treatment and surgical tissue tumor and peripheral blood samples will be collected for translational analysis.<br>• Tumor tissue at baseline will be assessed for PD-L1 expression via IHC.<br>Key exclusion criteria:<br>• Patients with non-squamous cell carcinoma histology;<br>• Patients with advanced inoperable or metastatic esophageal cancer;<br>• Brain metastases, leptomeningeal or bone metastases.<br>• Subjects with active, known or suspected autoimmune disease. Subjects with vitiligo, type I diabetes mellitus, residual hypothyroidism due to

	autoimmune condition only requiring hormone replacement, psoriasis not requiring systemic treatment, or conditions not expected to recur in the absence of an external trigger are permitted to enroll<br>• Subjects with a condition requiring systemic treatment with either corticosteroids or other immunosuppressive medications within 14 days of study drug administration. Inhaled or topical steroids, and adrenal replacement doses > 10 mg daily prednisone equivalents are permitted in the absence of active autoimmune disease.<br>• Accepted or are on the process of other chemotherapy, radiotherapy, targeted therapy with an immunotherapy or other anti-tumor drugs.
Study Assessment	Primary endpoint assessment:<br>• Feasibility of neoadjuvant adebrelimab will be based on patients proceeding to surgery without extended treatment related delays. Feasibility will be evaluated as the successful completion of neoadjuvant treatment and proceeding to surgery without any extended treatment-related delays. Extended treatment-related delay is defined as >37 days from pre-scheduled Day 0 in this context (>30 days from pre-scheduled day 0 plus 7 extra days to allow for OR scheduling constraints etc).<br>• Safety assessments will also be made by CTCAE version 5.0 criteria. Safety will be measured by:<br>❖ Frequency of drug related adverse events occurring up to 90 days after the last dose of adebrelimab or 30 days after surgery (whichever is longer).<br>❖ Frequency of serious adverse events occurring up to 90 days after the last dose of adebrelimab or 30 days after surgery (whichever is longer).<br>❖ Frequency of clinical laboratory test by worst toxicity grade (as assessed at the time intervals outlined in the study calendar)<br>Second endpoint assessment:<br>• pCR: Pathologic response to neoadjuvant therapy is secondary endpoint of the study. This will be assessed by percentage of viable tumor cells,

	percent tumor necrosis, amount of fibrosis and proliferation by phosphohistone H3. These factors will be assessed in a continuum over serial time-points including baseline, prior to dose two and surgical resection samples.<br>• RFS: It is defined as the time from the date of surgery (R0 resection) to the date of first recurrence (local, regional or distant) or enrollment assessed up to 24 months. Recurrence is examined by CT, PET-CT and/or upper endoscopy.<br>• OS: in the intent-to-treat population, which ends with the date of death of any causes since the date of randomization assessed up to 24 months. For patients alive at study closure, the survival time will be censored at time of last known survival status.<br>• R0 rate. No vital tumor is presented at the proximal, distal, or circumferential resection margin, then it is considered to be R0 resection. If a vital tumor is shown at 1 mm or less from the proximal, distal, or circumferential resection margin, it is considered to be microscopically positive (R1). Resection with macroscopic residual tumor is deemed R2 resection.
Statistical Considerations	Sample Size:<br>A total of 30 patients will be enrolled without randomization in this study. Patient enrollment is expected at a rate of 4-6 patients per month.<br>About sample size, a Simon optimal two-stage design was used. 70% of feasibility was considered unacceptable, and 90% of feasibility was considered promising. This design allows early study termination for excessive surgery delay. The probabilities of type I and type II errors were set at 0.05 and 0.2, respectively.<br>Six patients will be accrued to the first stage, and if five or more patients proceed to surgery without extended treatment related delays, 21 patients would be enrolled on the second stage. If more than 23 of the 27 patients proceed to surgery without extended treatment related delays, this regimen would be considered worthy of further testing.

2 Study design Schema

2.1 Study design

This is a prospective, single center, single arm, nonrandomized Phase IB study designed to detect feasibility and safety of neoadjuvant PD-L1 blockade in resectable ESCCs. 30 patients received up to two upfront doses of neoadjuvant adebrelimab monotherapy (adebrelimab 20 mg/kg, intravenously every 3 weeks, totally two cycles at Day 1 and Day 22) followed by surgery in treatment naïve adult (18~75 years old) subjects with clinical stage T2-4AN0-2M0, resectable, locally advanced ESCCs. Patients with subsequent adjuvant therapy including chemotherapy, chemoradiotherapy or anti-PD-1 immunotherapy will be acceptable.

Neoadjuvant therapy will be administered over 6 weeks and will then be followed by restaging scans. If at any point during the course of neoadjuvant therapy there is clinical (worsening performance status) and/or objective evidence (new imaging data) suggesting rapid disease progression, the patient will be taken off study and offered immediate surgery or other alternative treatment plan. If disease remains resectable based on the updated scans and the assessment of the treating surgical oncologist, patients will undergo definitive surgical excision of visible disease. Preliminary assessment of surgical margins will be obtained by intra-operative frozen section when appropriate. If surgical margins are found to be microscopically involved, patients will still be eligible for initiation of adjuvant therapy and no adjuvant radiation will be administered.

Patients will have a baseline biopsy and blood for genomic and immunologic analyses. All acquired tissue samples (baseline and surgical resection) will be used for biomarker analyses. Peripheral blood for biomarker analysis will be obtained at baseline, on-treatment, posttreatment and 1 month after surgery (if feasible).

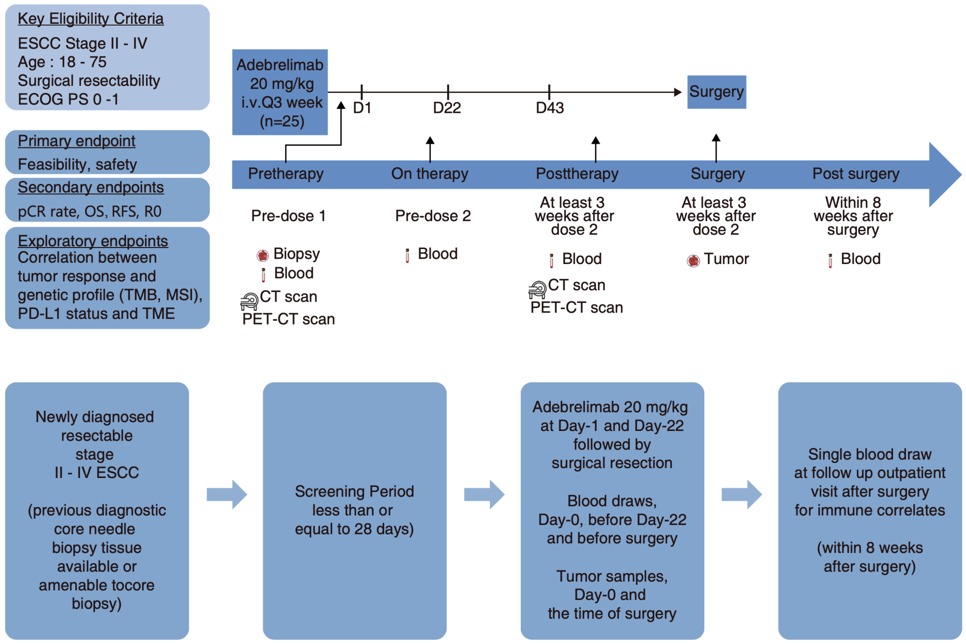

Figure 1 Study schema

2.2 Study Assessments

The primary objective of this study is assessment of feasibility and safety. Blood and tumor are required at baseline (samples must be obtained within 28 days of treatment initiation), at day 1 of neoadjuvant therapy and at the time of definitive surgical excision.

Secondary objectives of this study include assessment of pCR, RFS, OS, R0 rate. Exploratory objectives include immunologic changes in the tumor microenvironment and blood, treatment response based on imaging in response to neoadjuvant therapy, toxicity, RFS and OS.

Subjects will be assessed with computed tomography (CT), magnetic resonance imaging (MRI) or Positron emission tomography (PET-CT) at screening, after completion of neoadjuvant therapy and during the post-treatment follow-up period. Subjects will also be followed for survival. Subjects will be followed for a minimum of two years. Safety will be evaluated by clinical assessments including vital signs and complete physical examinations, chemistry and hematology laboratory values and formal assessments of adverse events (AEs).

2.3 Treatment Assignment

Subjects will be approved for treatment after a consensus panel of medical and surgical oncologists have determined that the disease is amenable to surgical resection and after the subject has passed screening evaluations. There will be no randomization for patients in this study.

3 Objectives

3.1 Primary objectives

3.1.1 To assess the efficacy of neoadjuvant adebrelimab for locally advanced resectable ESCC [clinical stage, T2-4AN0-2M0], as measured by feasibility, based on patients proceeding to surgery without extended treatment related delays.

Feasibility of neoadjuvant adebrelimab will be based on patients proceeding to surgery without extended treatment related delays. Feasibility will be evaluated as the successful completion of neoadjuvant treatment and proceeding to surgery without any extended treatment-related delays. Extended treatment-related delay is defined as >37 days from pre-scheduled Day 0 in this context (>30 days from pre-scheduled day 0 plus 7 extra days to allow for OR scheduling constraints etc).

3.1.2 To assess the safety of neoadjuvant adebrelimab in safety run-in phase, as measured by DLT, based grade 3-4 toxicities including liver, GI, renal, pneumonitis and any other grade 3-4 toxicity.

Safety assessments will also be made by CTCAE version 5.0 criteria. Safety will be measured by:

- ❖ Frequency of drug related adverse events

occurring up to 90 days after the last dose of adebrelimab or 30 days after surgery (whichever is longer).

- ❖ Frequency of serious adverse events

occurring up to 90 days after the last dose of adebrelimab or 30 days after surgery (whichever is longer).

- ❖ Frequency of clinical laboratory test by worst

toxicity grade (as assessed at the time intervals outlined in the study calendar)

3.2 Secondary objectives

3.2.1 To assess the pathological complete response (pCR) rate to neoadjuvant adebrelimab in resected tumor and lymph nodes.

pCR is to be assessed in the resected specimen following neoadjuvant therapy

using standardized work up of the resection specimen in the pathology department and standardized histological criteria for tumor regression grading. The degree of histomorphologic regression is clarified into four categories as follows: grade 1, no evidence of vital residual tumor cells (pCR); grade 2, less than 10% vital residual tumor cells; grade 3, 10 to 50%; and grade 4, more than 50% according to previous report.

3.2.2 To assess recurrence-free survival in patients receiving neoadjuvant adebrelimab in this study.

Recurrence-free survival (RFS): It is defined as the time from the date of surgery (R0 resection) to the date of first recurrence (local, regional or distant) or enrollment assessed up to 24 months. Recurrence is examined by CT, PET-CT and/or upper endoscopy.

3.2.3 To assess overall survival in patients receiving neoadjuvant adebrelimab in this study.

Overall survival (OS): in the modified intent-to-treat population, which ends with the date of death of any causes since the date of randomization assessed up to 24 months. For patients alive at study closure, the survival time will be censored at time of last known survival status.

3.2.4 To assess the R0 rate.

No vital tumor is presented at the proximal, distal, or circumferential resection margin, then it is considered to be R0 resection. If a vital tumor is shown at 1 mm or less from the proximal, distal, or circumferential resection margin, it is considered to be microscopically positive (R1). Resection with macroscopic residual tumor is deemed R2 resection.

3.3 Exploratory research

3.3.1 To investigate differences in genetic profile (TMB, MSI) as well as expression of selected immune markers in patients who will response to adebrelimab; to determine differences in the quality and quantity of tumor infiltrating lymphocytes.

3.3.2 To determine differential regulations of immune checkpoint pathways and tumor immune microenvironment phenotypes in patients who will response to adebrelimab.

3.3.3 To determine the effect of neoadjuvant immunotherapy on the local and systemic immune status of ESCC.

3.3.4 To explore the association between adebrelimab exposure and selected pharmaco-dynamic markers in the peripheral blood and in the tumor microenvironment, including measurement of T cell receptor (TCR) clonotypes.

4 Study endpoints assessments

4.1 Efficacy Endpoint

4.1.1 Primary Efficacy Endpoint

Feasibility of neoadjuvant adebrelimab will be based on patients proceeding to surgery without extended treatment related delays. A treatment related delay will be considered “extended” if it is greater than 37 days following the initially planned surgery date. For feasibility, an adebrelimab toxicity of any grade, that in the judgment of the investigator or surgeon could adversely impact perioperative morbidity or mortality, should delay the planned operative date.

4.2 Secondary Efficacy Endpoint

4.2.1 Overall Survival (OS)

Overall survival (OS): in the intent-to-treat population, which ends with the date of death of any causes since the date of randomization assessed up to 24 months. For patients alive at study closure, the survival time will be censored at time of last known survival status.

4.2.2 Recurrence-free Survival (RFS)

Investigator assessed, it is defined as the time from the date of surgery (R0 resection) to the date of first recurrence (local, regional or distant) or enrollment assessed up to 24 months. Recurrence is examined by CT, PET-CT and/or upper endoscopy.

4.2.3 Pathological complete response rate (pCR)

pCR is to be assessed in the resected specimen following neoadjuvant therapy using standardized work up of the resection specimen in the pathology department and standardized histological criteria for tumor regression grading. The degree of histomorphologic regression is clarified into four categories as follows: grade 1, no evidence of vital residual tumor cells (pCR); grade 2, less than 10% vital residual tumor cells; grade 3, 10 to 50%; and grade 4, more than 50% according to previous report

4.2.4 R0 resection rate

No vital tumor is presented at the proximal, distal, or circumferential resection margin, then it is considered to be R0 resection. If a vital tumor is shown at 1 mm or less from the proximal, distal, or circumferential resection margin, it is considered to be microscopically positive (R1).

4.3 Safety Endpoint

4.3.1 Primary Safety Endpoint

- Dose-limiting toxicity (DLT) of Patients in safety run-in phase will be assessed. The primary DLTs of concern for safety monitoring will be grade 3-4 toxicities including liver, GI, renal, pneumonitis and any other grade 3-4 toxicity that in the opinion of the investigator significantly interfered with the subjects' optimal perioperative management. They will be monitored continuously for six patients in the run-in phase through day 90 following the last dose of adebrelimab (or day 30 post-surgery, whichever is longer) for the last patient in the run-in. If three of the first six patients experience DLT, the study will be discontinued. Overall, the study will continue to the second stage if none or one of the 6 patients experience DLT in the run-in phase. If two patients experience grade 3-4 DLT, the study will be paused for a review and may or may not continue at the discretion of the investigators.
- Safety assessment will be made according to Common Terminology Criteria for Adverse Events CTCAE 5.0 criteria.

Safety endpoints include TEAE, clinical laboratory test parameters, vital sign, electrocardiogram, echocardiogram and Eastern Cooperative Oncology Group (ECOG) performance status (PS).

5 Background and Rationale

5.1 Introduction

Esophageal cancer was the seventh most frequently diagnosed cancer and the sixth leading cause of cancer-related death worldwide in 2020(*1*). Esophageal squamous cell carcinoma (ESCC) and adenocarcinoma are the two most common histologic subtypes, of which 90% of esophageal cancer cases in Asia and sub-Saharan Africa are ESCC(*2*). A large proportion of patients with ESCC are diagnosed at advanced stages because of the lack of distinguishing clinical indications and ultimately have poor prognosis, with the 5-year survival rate ranging from 15% to 25%(*3*). So far, surgical resection is still the most effective treatment for esophageal cancer, but for locally advanced esophageal cancer (cT2-4AN0-2M0), the 5-year survival rate of surgical resection alone is low, the metastasis and recurrence rates are high(*4*), and the curative effect is not good. Therefore, the treatment of esophageal cancer has changed to a multimodal comprehensive treatment program(*5*).

Host immunity is fundamental to the suppression of human cancer and conversely host immune evasion by tumor cells is an essential pathway in the development of human cancer(*6*). The concept of cancer immune editing is well described in animal models whereby tumors are capable of subverting host immunity despite developing frequent genetic aberrations with the potential to generate immunogenic neo-antigens(*7*). The three phases of immune editing are as follows: elimination (host immune system responds to tumor neo-antigens and destroys tumor cells), equilibrium (immune evasive tumor cells persist however growth and metastasis is restrained by residual host immunity) and escape (tumor cells overcome immune control and can develop into clinically evident cancers). The development of clinically apparent tumors indicates failure of the host immune system to recognize and destroy incipient cancers. This is due to induction of immune tolerance among tumor-specific T cells as well as expression of immune inhibitory ligands termed checkpoints. These ligands bind to receptors on T cells that signal to down-modulate effector functions such as cytokine production and killing activity.

Consequent strategies aimed at augmenting host anti-tumor immunity are attractive with potential for long-term tumor control or even cure if persistent immune responsiveness can be engendered particularly in earlier stages of disease. The integration of immune checkpoint inhibition into the treatment algorithm of ESCC marks a new era of treatment of this dismal disease. Treatment of esophageal carcinoma has changed dramatically following several landmark trials, which have proven the benefit of immunotherapy. The selective PD-1 inhibitor nivolumab has been shown to improve DFS in the

adjuvant therapy setting (CheckMate-577). In the first-line treatment, PD-L1 positive (CPS ⩾ 10) squamous cell carcinoma patients (pts) have been shown to have an increased OS following treatment with the PD-1-inhibitor pembrolizumab in combination with chemotherapy (KEYNOTE-590). Nivolumab also improved overall survival in the first line setting either combined with ipilimumab or with chemotherapy (CheckMate 648) compared to chemotherapy alone. In Asian first-line patients, phase III trials investigating camrelizumab (ESCORT1), toripalimab (JUPITER 06), or sintilimab (ORIENT 15) in addition to chemotherapy also showed significant survival benefits. In the second-line setting, monotherapy with nivolumab (ATTRACTION-03), pembrolizumab (KEYNOTE-181), camrelizumab (ESCORT), and tislelizumab (RATIONALE 302) demonstrated a benefit in OS in comparison to chemotherapy. Here we will review these trials and integrate them into the current treatment algorithm.

Table 1 – Immunotherapy in ESCC

Clinical Trials	Treatment	Pt No	ORR (%)	TRAE 3/4 (%)	mOS (month)	Ref.
**First-line**						
KEYNOTE-590	Pembrolizumab +CT	373	-	72	12·6	(*8*)
CheckMate-648	Nivolumab+CT	321	47	47	13.2	(*9*)
	nivolumab+ipilimumab	325	28	32	12.8	
JUPITER-06	Toripalimab+CT	514	69.3	73.2	17	(*10*)
RATIONALE-311	Tislelizumab plus concurrent chemoradiotherapy	366	-	-	-	(*11*)
**Second-line**						
ATTRACTION-3	Nivolumab	210	19	18	10.9	(*12*)
KEYNOTE-181	Pembrolizumab	198	16.7	18.2	9.3	(*13*)
ESCORT	Camrelizumab	457	20.2	19	8.3	(*14*)
**Adjuvant therapy**						
CheckMate-577	Nivolumab	532	-	13	mDFS : 22.4	(*15*)
**Neoadjuvant therapy**						
PALACE-1	Pembrolizumab +CRT	20	-	65	-	(*16*)

KEYSTONE-001	Pembrolizumab+ CT	42	96.6	0	-	(*17*)
ESPRIT	Camrelizumab+C T	48	66.7	2.1	-	(*18*)
TD-NICE	Tislelizumab+CT	45	-	42.2	-	(*19*)

Treatment-related grade 3/4 adverse events
ORR: Objective Response Rate;
mOS: median overal survival

5.2 Programmed death-1 – Molecular Biology

Programmed death-1 (PD-1 or CD279), primarily expressed on activated T cells, B cells and myeloid cells, is a 55 kD type I transmembrane protein that is a member of the CD28 family of T-cell co-stimulatory receptors that also includes CD28, CTLA-4, ICOS and BTLA(*20, 21*). Two ligands specific for PD-1 have been identified: PD-L1 (also known as B7-H1 or CD274) and PD-L2 (also known as B7-DC or CD273), each of which are primarily expressed on antigen presenting cells. PD-L1 and PD-L2 have been shown to downregulate T-cell activation upon binding to PD-1 in both murine and human systems(*22*). PD-1 has been shown to inhibit CD28-mediated upregulation of IL-2, IL-10, IL-13, IFN-γ and Bcl-xL. PD-1 expression has also been noted to inhibit T cell activation and expansion of previously activated cells. PD-1 contains an intracellular membrane proximal immunoreceptor tyrosine inhibitory motif (ITIM) and a membrane distal immunoreceptor tyrosine based switch motif (ITSM). Both Src homology region 2 domain-containing phosphatase (SHP) -1 and -2 have been found to bind to the cytoplasmic tail of PD-1 and mediate its signaling. Once this signaling has occurred, PD- 1 binds to the phosphorylated tyrosine residue in the ITSM in its cytoplasmic region and mediates the suppressive effects of PD-1(*23, 24*).

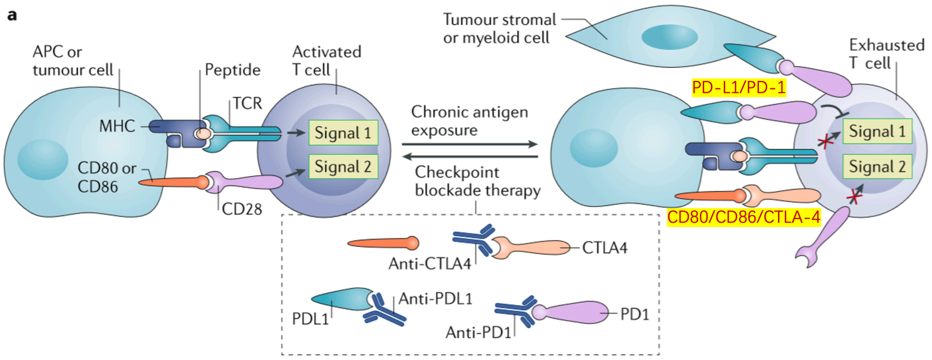

Figure 2 Blockade mechanism of PD-1/PD-L1axis

5.3 Programmed Death Ligand -1 – Expression in Humans

In humans, constitutive PD-L1 expression is normally limited to macrophage-

lineage cells, although expression of PD-L1 can be induced on other hematologic cells as well including activated T cells. Aberrant expression of PD-L1 by tumor cells (retrospectively detected by immunohistochemistry, IHC) has been reported in a number of human malignancies(*25*). Positive PD-L1 expression in esophageal cancer approxiamately account for 18%.

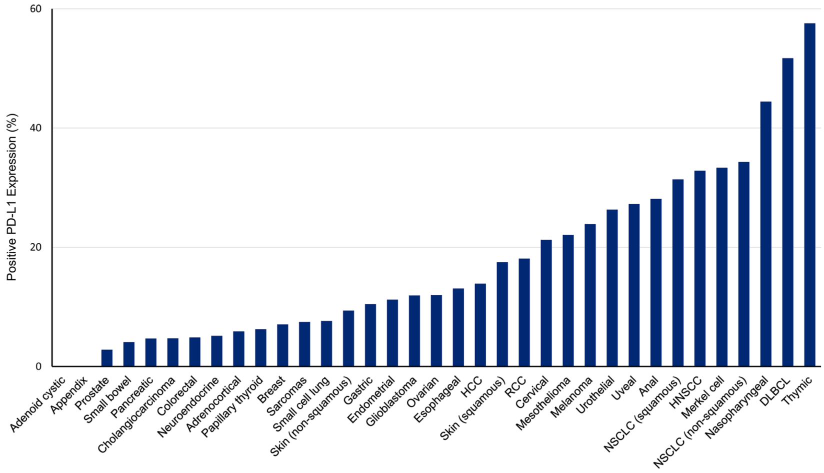

Figure 3 Landscape of PD-L1 expression across the major tumor types. Percentage of tumors with positive PD-L1 expression by IHC within 35 major tumor types, from the lowest frequency of positivity (left) to the highest frequency (right).

5.4 Rationale for neoadjuvant immunotherapy

Increasingly, immunotherapy is being given in the neoadjuvant setting (prior to surgery) in variety of cancer types including breast(*26*), lung(*27*), rectal(*28*), and melanoma cancers(*29*). There are many practical and theoretical advantages to giving systemic immunotherapy up front. Scientific evidence that supports two different but not mutually exclusive models by which neoadjuvant PD-(L)1 blockade may promote systemic antitumor immunity(*30*).

- First, anti-PD-(L)1 rejuvenates tumor-specific cytotoxic T cells that already reside in the TME, causing their activation, proliferation, and trafficking to micrometastatic deposits.
- Second, tumor-draining lymph nodes (TDLN) appear to be the focal point for anti-PD-(L) 1 activity, where dendritic cell presentation of tumor antigens to T cells is enhanced; these tumor-specific T cells then enter the bloodstream and migrate to tumor sites.

The destruction of micrometastases is central to the notion that neoadjuvant PD-(L)1 blockade should result in enhanced relapse-free and overall survival in operable patients who would otherwise relapse after surgery alone. There is a strong rationale for evaluating neoadjuvant immunotherapy across tumor types. Presurgical drug administration provides abundant on-therapy tissue for in-depth mechanistic and biomarker studies.

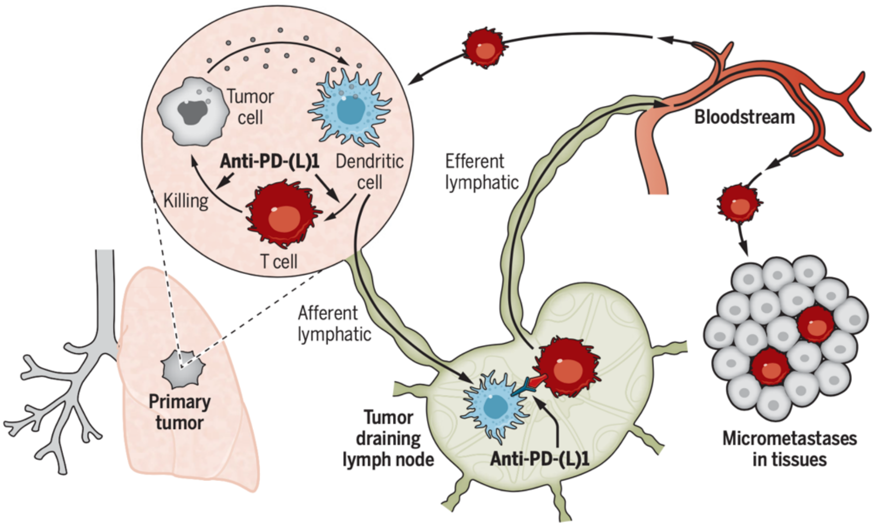

Figure 4 Two potential mechanisms for the enhancement of systemic antitumor T cell immunity after neoadjuvant PD-(L)1 blockade.

PD-(L)1 blockade could result in the “in situ” expansion of tumor-specific T cell clones already within the tumor microenvironment. This expansion and activation is largely driven by PD-L1– and PD-L2–expressing dendritic cells in the tumor. Tumor-specific tumor-infiltrating lymphocytes may represent naïve T cells or T cells that have already been “primed” to tumor antigen before PD-1 pathway blockade. In addition, tumor antigen containing dendritic cells that originate in the tumor pick up tumor antigens and traffic to the tumor-draining lymph nodes, herethey present antigens either ineffectively or in a tolerogenic fashion to tumor-specific T cells. PD-(L)1 blockade could act at this point, enhancing productive stimulation of tumor-specific T cells or partially reversing toleruction. Activated T cells enter the circulation by way of efferent lymphatics and then egress into tissues.

To date, published reports of neoadjuvant anti-PD-(L)1-based immunotherapies have centered on relatively small investigator-initiated clinical trials with a rich correlative scientific component. In 2018, Forde et al. presented the first literature report of neoadjuvant anti-PD-1 therapy, describing an investigator-initiated phase 2 trial of nivolumab in 21 patients with high-risk (stages I to IIIA) NSCLC(*27*). In this study, previously untreated patients received nivolumab preoperatively for a brief 4-week period. Surgery was then carried out according to standard practice, resecting the primary tumor mass along with surrounding normal lung tissue and TDLN. The treatment regimen was found to be safe, with no surgical delays, no unanticipated toxicities, one treatment-related grade 3 adverse event, and one patient who was found to have an unresectable tumor intraoperatively. Particularly revealing was a comparison of tumor response assessments conducted radiographically, with computerized tomographic (CT) scans at baseline and just before surgery, or histologically by examining the surgical specimen for evidence of pathologic response. Although partial radiographic responses (defined as ⩾ 30% decrease in the sum of tumor diameters) were observed in only 2 of 21 (10%)

patients, major pathologic responses at the primary tumor site (⩽10% viable tumor cells remaining) were seen in 9 of 20 (45%) operable cases. Thus, conventional radiographic studies underestimated the extent and rapidity of pathologic responses, which were characterized by an influx of immune cells and proliferative fibrosis, which is consistent with an immunological mechanism. The degree of pathologic response significantly correlated with tumor mutational burden and with the computationally predicted neoantigen burden. Furthermore, an in-depth study of one patient revealed that neoantigen-specific T cell clones that were present intratumorally and in TDLN expanded in the peripheral blood during preoperative nivolumab therapy; these clones persisted in the periphery for weeks after tumor resection.

5.5 Development of Adebrelimab

Adebrelimab (Hengrui Pharmaceuticals, SHR-1316) is a fully human, IgG4 (kappa) monoclonal antibody that binds PD-1 with high affinity blocking its interactions with its ligands PD-L1 (B7-H1) and PD-L2 (B7-DC) and increasing tumor antigen specific T cell proliferation and cytokine secretion. In a phase 1 trial in patients with solid advanced tumours, adebrelimab showed preliminary antitumour activity and acceptable safety, with no dose limiting toxicity reported up to a dose of 20 mg/kg every 3 weeks(*31*).

Adebrelimab is currently in clinical development for the treatment of extensive-stage small-cell lung cancer including studies in the first-line combined with carboplatin and etoposide in CAPSTONE-1 trial(*31*), as well as for the treatment of resectable stage II-III NSCLC in the peroperative setting combined with chemotherapy(*32*). The clinical efficacy and tolerability of adebrelimab have been demonstrated in the first-line combinated with liposomal irinotecan and 5-fluorouracil in advanced ESCC(*33*).

In the proposed study the safety and feasibility of preoperative adebrelimab will be assessed as well as the impact of preoperative anti-PD-L1 blockade on anti-tumor immunity in resectable ESCC.

5.6 Clinical experience with adebrelimab

The results of a randomized, double-blind, placebo-controlled phase 3 CAPSTONE-1 study (NCT03711305) recently evaluated the safety and effectiveness of adebrelimab as a first-line treatment for extensive stage SCLC patients (*31*). 73 patients randomly received adebrelimab (20mg/kg, the first day of each 21 day cycle)+carboplatin+etoposide or placebo+carboplatin+etoposide at a ratio of 1:1 for an induction period of 4 – 6y cycles, and then maintained adebrelimab or placebo until the investigator evaluated the progress of the disease using the solid tumor response evaluation standard version 1.1 (RECIST v1.1). A total of 462 patients were

enrolled, 230 patients received adebrelimab plus chemotherapy (adebrelimab group), and 232 patients received placebo plus chemotherapy (placebo group). All treatments were IV administered. At the data cutoff (October 8, 2021), the median follow-up time was 13.5 months (IQR 8.9 - 20.1). Median OS was significantly improved in the adebrelimab group (median 15.3 months [95% confidence interval 13.2-17.5]), while in the placebo group (12.8 months [11.3-13.7]; hazard ratio 0.72 [95% confidence interval 0.58-0.90]; unilateral dp=0.0017). The most common treatment-related grade 3 or 4 adverse events were decreased neutrophil count (174 [76%] patients in the adebrelimab group and 175 [75%] patients in the placebo group), decreased white blood cell count (106 [46%] and 88 [38%]), decreased platelet count (88 [38%] and 78 [34%]), and anaemia (64 [28%] and 66 [28%]). Treatment-related serious adverse events occurred in 89 (39%) patients in the adebrelimab group and 66 (28%) patients in the placebo group. Four treatment-related deaths were reported: two each in the adebrelimab group (respiratory failure and interstitial lung disease and pneumonia) and placebo group (multiple organ dysfunction and unknown cause of death).

Furthermore, a multicentre, open-label study evaluated the efficacy and safety of adbrelimab plus liposomal irinotecan and 5-fluorouracil as the first-line treatment for patients with advanced ESCC(*33*). Eligible patients received adbrelimab (10 mg/kg), liposomal irinotecan (60 mg/m2 for the first cycle, 80 mg/m2 thereafter), and 5-fluorouracil (2400 mg/m2) every 14 days until disease progression, intolerable toxicity or withdrawal of consent. A total of 23 patients were enrolled between 11 March 2019 and 31 May 2019. The median follow-up duration was 15.2 months (95% CI 14.2–16.2). The median PFS was 8.5 months (95% CI 1.2–15.8), and objective response rate (ORR) and disease control rate (DCR) were 52.2% (95% CI 30.1–74.3) and 73.9% (95% CI 54.5–93.3), respectively. The median OS was 11.6 months (95% CI 6.7–16.6). The most common treatment-related grade 3–4 adverse events (AEs) were neutropenia (17.4%), nausea (13.0%), and anorexia (13.0%). Treatment-related serious AEs occurred in two patients. No treatment-related deaths occurred.

Based on the positive clinical outcomes of immunotherapy in neoadjuvant and adjuvant settings(*31, 33*), a randomized, double-blind, multicenter, phase 1b/3 trial evaluated the efficacy and safety of adebrelimab plus chemotherapy vs placebo plus chemotherapy as perioperative treatment for resectable NSCLC(*32*). A total of 37 patients were enrolled and received planned neoadjuvant therapy. As of data cutoff on Jan 25, 2022, 19 (51.4%, 95% CI 35.9-66.6) of the 37 patients achieved Major pathologic response (MPR) per blinded independent pathological review (BIPR) and 11 (29.7%, 95% CI 17.5-45.8) patients achieved pathological complete response. 26 (70.3%, 95% CI 54.2-82.5) patients had an objective response per RECIST v1.1. The 12-month

event-free survival (EFS) rate was 77.8% (95% CI 54.1-90.3). 29 (78.4%) patients had grade ≥3 treatment-related AEs and nine (24.3%) had treatment-related serious AEs. No treatment-related deaths occurred. Grade ≥3 surgery-related AEs within 30 or 90 days after surgery were both reported in 5 (14.7%) patients.

Adebrelimab combined with chemo-radiotherapy is being evaluated in a placebo-controlled, multicenter, randomized, double-blind, phase 3 trial (NCT04691063), in which patients with limited SCLC were recruited. In addition, a randomized, double-blind, multicenter, phase 1b/3 study (NCT04316364) is evaluating adebrelimab or placebo combined with chemotherapy as perioperative treatment for resectable stage II or III NSCLC(*32*).

5.7 Rationale for neoadjuvant adebrelimab in ESCC, dosing and schedule and inclusion/exclusion criteria

5.7.1 Rationale for neoadjuvant immunotherapy in ESCC

Neoadjuvant therapy can improve the prognosis of patients with esophageal cancer. Immunotherapy with PD-1/PD-L1 antibody has achieved good results in neoadjuvant therapy of advanced lung cancer, and no treatment-related deaths have occurred, which is safe and effective. Currently, immunotherapy (IO) agents have led to significant improvements in the first-line and second-line settings in advanced-stage ESCC diseases(*12, 34-36*) with good objective response rates and no treatment-related deaths(*10, 37*), but not been approved in the preoperative setting.

- At present, there are no clinical studies on the application of neoadjuvant immunotherapy in resectable esophageal cancer, and the safety and efficacy of neoadjuvant immunotherapy are not clear.
- At present, there is no specific study on the immune microenvironment of esophageal cancer, and the biomarkers for the sensitivity judgment of esophageal cancer immunotherapy need to be further explored.

The benefit of IO combined with neoadjuvant chemotherapy (nICT) or chemoradiation (nICRT) is being explored in a number of ongoing phase II and III trials including HCHTOG1909(*38*), KEYSTONE-002(*39*), PERFECT(*40*). nICT and nICRT were expected to improve pCR rates(*41*), however, a meta-analysis showed that the estimated rates of pCR of nICRT and nICT (32.7% vs. 26.3%, P = 0.37) were comparable with nCRT of 35.7% in our ESCC-based CMSG1701 trial (*42*). Additionally, data from esophageal adenocarcinoma demonstrated that additional atezolizumab to nCRT did not improve median OS (29.7 vs. 34.3 mons, P = 0.43) (*40*). The aforementioned results indicate that the clinical benefits of IO combined nCRT or nCT still remain controversial. More importantly, precision medicine should be guided by an understanding of

mechanisms underpinning sensitivity/resistance - an evidence-based approach rather than empirical random combination with available therapies.

The proposed, single-arm, open, single-center, Phase IB clinical study will evaluate the safety and feasibility of neoadjuvant administration adebrelimab in patients with locally advanced resectable ESCC (clinical stage T2-4aN0-2M0) and will facilitate a comprehensive exploratory characterization of the tumor immune milieu, genetic profile, circulating immune cells and clonotypic dynamic of intratumoral immune cells in these patients. Data obtained in this study will provide solid evidence for planning further prospective clinical trials of anti-PD-L1 in ESCC. Importantly, it is highly desirable to identify prospective biomarkers of response and toxicity allowing patients with ESCC who are most possibly to derive benefit to receive checkpoint inhibition treatment, and conversely to prune the risk of toxicity and ineffective/unnecessary treatment for patients who are unlikely to benefit.

5.7.2 Rationale for Dosing and Schedule

The dose of adebrelimab was based on a phase 1 trial in Chinese patients with advanced solid tumors (NCT03474289; principal investigator, Prof. Xichun Hu, Fudan University Shanghai Cancer Center; unpublished data).
In this dose-escalation (3+3 design) and expansion trial enrolling a total of 41 patients, 4 dose levels were evaluated (3 mg/kg/Q3W, 10 mg/kg/Q3W, 20 mg/kg/Q3W, and 10 mg/kg/Q2W). There was no dose-limiting toxicity observed during the dose-escalation phase, and the maximum tolerated dose was not reached. No obvious correlations were detected between the dose of adebrelimab and the incidences of treatment-related adverse events (TRAEs), grade ≥3 TRAEs, and treatment-related serious adverse events. In terms of efficacy, the objective response rate was 33.3% (1/3), 23.1% (3/13), 23.1% (3/13), and 25.0%(3/12) in the 3 mg/kg/Q3W, 10 mg/kg/Q3W, 20 mg/kg/Q3W, and 10 mg/kg/Q2W groups, respectively. There was no apparent correlation between dose and clinical efficacy.

Adebrelimab exposure escalated in a dose-proportional manner over the dose range of 3-20 mg/kg/Q3W. After multiple administrations, adebrelimab reached steady state after 3-5 treatment cycles, with no detectable accumulation at steady state. Receptor occupancy of PD-L1 remained above 90% before and after the steady state and upon treatment discontinuation in all dose groups except for the 3 mg/kg/Q3W group.

The dose of 20 mg/kg as well as Q3W dosing frequency were adopted from previous published studies in resectable non small-cell lung cancer (NSCLC) (*32*) and extensive-stage small-cell lung cancer (*31*). In this trial, two cycles of adebrelimab were administered based on the fact that current most neoadjuvant therapy studies are using two cycles of treatment.

5.7.3 Rationale for Main Inclusion and Exclusion Criteria

The choice of T2-4aN0-2M0 ESCC was made as these patients have a high risk of tumor relapse and death with upfront operation. Although current standard therapy including neoadjuvant chemo/radiotherapy has extended the OS and DFS significantly in these patients, the long-term follow up of CROSS trial and NEOCRTEC5010 trial both showed that there are still a considerable portion of patients suffered from relapse and recurrence. Distant relapse occupies vast majority of these recurrent patients, thus there is an urgent need for improved, novel and enhanced systemic therapies.

Preclinical studies revealed that anti-PD-1/PD-L1 inhibitor in combination with chemotherapy can further enhance the immune response and inhibit the immune escape for cancer cells(*43*). Emerging evidence has suggested that immunotherapy can improve overall survival in unresectable tumors with manageable safety profile(*8, 12-14*). In addition, the efficacy of neoadjuvant therapy with PD-1 inhibitors has been reported in lung cancer(*27*). Considering these promising results, it is conceivable that neoadjuvant immunotherapy will provide survival benefit with manageable safety profile and cause no delay of scheduled operation.

Patients who are assessed clinically by their surgeon as possibly requiring a pneumonectomy to obtain complete surgical resection of their primary tumor have been excluded from this pilot study, as these patients have a significantly higher risk of postoperative complications including respiratory distress. In addition, stage IIIA patients with N2 disease have been excluded as these patients may require neoadjuvant chemotherapy and/or radiation as standard therapy.

The choice of a single arm pilot study was made to allow initial assessment of the safety and feasibility of preoperative anti-PD-1. Data obtained from the proposed study of patients treated with preoperative anti-PD-1 will be compared with untreated patients proceeding directly to surgery on a companion tissue collection protocol.

6 Patient Population

6.1 Inclusion Criteria

6.1.1 Men and women age ≥ 18 years, ≤ 75 years.

6.1.2 Histologically proven ESCC (core biopsy required).

- Patients who were diagnosed as squamous cell carcinoma by

gastroscopic biopsy and whose tissue specimens were taken before treatment.

- The primary tumor was located in the chest. The primary site of esophageal cancer was determined by the position of the esophagus where the upper edge of the mass was located (upper thoracic esophagus: from the thoracic inlet to the level of the lower edge of the azygos vein arch, 20 cm to < 25 cm from the incisor by endoscopy; Esophagus in the middle part of the chest: from the lower edge of the azygos arch to the level of the inferior pulmonary vein, 25 cm to < 30 cm from the incisor on endoscopy; Lower thoracic esophagus: above the level of the inferior pulmonary vein and down to the stomach. Endoscopy: 30cm to < 40cm from the incisor.

6.1.3 Stage

- Pre-treatment stage as cT2-4aN0-2M0 (AJCC/UICC 8th Edition) (In case of stage cT4a, curative resectability has to be explicitly verified by the experienced surgical investigator);

  ESCC with resection option for potential cure, as diagnosed by gastroscopic biopsy. According to the above examinations, patients with esophageal cancer that can be resected clinically before operation (PET-CT, chest and abdomen enhanced CT, cervical lymph node ultrasound and other means to assess whether the tumor has obvious invasion, whether the mediastinal lymph nodes have obvious enlargement, whether there is distant organ metastasis; If the primary tumor was suspected to be T4, multiple mediastinal lymph node metastases or distant metastases, the whole-body PET-CT and endoscopic ultrasonography (EUS) (optional) were performed to further clarify the clinical stage. This includes clinical stage II-IVA, Physical condition score ECOG 0 ~ 1 estimated survival period ≥ 12 months.

6.1.4 Adequate organ function as follows

- White blood cell (WBC) > 4.0 × 10^9/L
- Absolute neutrophil count (ANC) ≥ 2.0 × 10^9/L
- Platelet count > 100 × 10^9L
- Hemoglobin > 90 G/L
- Adequate lung function: Forced expiratory volume in 1 second (FEV1) ≥ 1.2L, FEV1% ≥ 50%, Diffusing capacity for carbon monoxide (DLCO) ≥ 50%
- Adequate liver function: Serum bilirubin was less than 1.5 times the maximum normal value, Alanine transaminase (ALT) and Aspartate transaminase (AST) below the maximum normal values

1.5 times.

- Adequate renal function: Serum creatinine (SCr) ≤ 120 μmol/L, creatinine clearance (CCr) ≥ 60 ml/min

6.1.5 The patients should be able to understand our research and sign the informed consent.

6.2 Exclusion Criteria

- Patients with non-squamous cell carcinoma histology;
- Patients with advanced inoperable or metastatic esophageal cancer;
- Pre-treatment stage as non cT2-4aN0-2M0 (AJCC/UICC 8th Edition);
- Non-curatively-resectable verified by the surgical investigator, AJCC/UICC 8th Edition);
- Patients with another previous or current malignant disease which is likely to interfere with treatment or the assessment of response in the judgement of the surgical investigator.
- Any patient with a significant medical condition which is thought unlikely to tolerate the therapies. Such as cardiac disease (e.g. symptomatic coronary artery disease or myocardial infarction within last 12 months), clinically-significant lung disease, clinically-significant bone marrow, liver, renal function disorder;
- Participation in another intervention clinical trial with interference to the immunotherapy intervention during this study or during the last 30 days prior to informed consent;
- Expected lack of compliance with the protocol.

6.2.1 Cancer-related criteria

- Subjects with the stage of tumor is T4b (AJCC/International Union Against Cancer (UICC) 8th Edition) which can not be resected according to imaging examinations like thoracic and abdominal enhanced CT, cervical lymph nodes ultrasound, whole body PET-CT scan (optional) or endobronchial ultrasonography (EBUS) (optional); several enlarged lymph nodes existed (≥3 estimated lymph nodes metastasis); multiple station enlarged lymph nodes existed (≥2 estimated stations of lymph nodes metastasis); distant metastasis existed.
- Endoscopic examination showing non-squamous carcinoma.
- Accepted or are on the process of other chemotherapy, radiotherapy, targeted therapy with an anti-PD-1, anti-PD-L1, anti-PDL-2, or anti-CTLA-4 antibody (or any other antibody targeting T cell co-regulatory pathways) or other anti-tumor drugs.

6.2.2 Other exclusion criteria

- Any active or history of chronic or autoimmune disease (including any history of inflammatory bowel disease), or history of syndrome that required systemic steroids or immunosuppressive medication
- Subjects who have severe systematic intercurrent disease, such as active infection or poorly controlled diabetes; coagulation disorders; hemorrhagic tendency or under treatment of thrombolysis or anticoagulant therapy.
- Subjects who have severe systematic intercurrent disease, such as active infection or poorly controlled diabetes; coagulation disorders; hemorrhagic tendency or under treatment of thrombolysis or anticoagulant therapy.
- Subjects who require or may require pneumonectomy, as assessed by their surgeon, to obtain potentially curative resection of primary tumor.
- Administration of other chemotherapy, radiotherapy or targeted therapy in the pre-operative period.
- Subjects with previous malignancies (except non-melanoma skin cancers, in situ bladder, gastric, breast, colon or cervical cancers/dysplasia).
- Subjects with a history of interstitial lung disease, pulmonary fibrosis, diverticulitis or systematic ulcerative gastritis.
- Active infection of HIV, hepatitis B virus (HBV), hepatitis C virus (HCV) or be HIV serum positive; or HBV, HCV RNA positive.
- History of severe hypersensitivity to antibody drugs and allergy to study drug components.
- History of congestive heart failure, angina without good control with medicine; ECG-proved penetrating myocardial infarction; hypertension with bad control; valvulopathy with clinical significance; arrhythmia with high risk and out of control.
- History of organ transplantation (including autologous bone marrow transplantation and peripheral stem cell transplantation.
- History of peripheral nerve system disorders, obvious mental disorders or central nerve system disorders.
- Women who are pregnant or nursing, or people at child bearing stage who are reluctant to use contraception measures
- Men with female partners (WOCBP) that are not willing to use contraception.
- Underlying medical conditions that, in the Investigator's opinion, will make the administration of study drug hazardous or obscure the interpretation of toxicity or adverse events.
- Prisoners or subjects who are involuntarily incarcerated or compulsorily detained for treatment of either a psychiatric or physical (e.g. infectious disease) illness

6.3 Withdraw Criteria

- Confirmed that it is unable to do resection due to the disease progression after neoadjuvant treatment;
- Patients requiring simultaneous surgical treatment for other diseases;
- Sudden severe comorbidities in the perioperative period (intolerable surgery or anesthesia), which are unsuitable or unable to implement the treatment protocol of this study as scheduled;
- Patients are confirmed to require emergency surgery according to the condition changes verified by attending doctors after being enrolled in this study;
- Patients are voluntary to quit or discontinue treatment due to personal reasons in any stage after being enrolled in this study;
- Treatment that proved to violate the study protocol.

7 Standard Operation Procedures (SOP)

Treatment Schedule

Treatment Phase	Screening	Neoadjuvant treatment	Preoperative evaluation	Operation	Follow up
Time point	<14 days before enrollment	Week 1-5 At day 1 of each cycle	Within 3-5 weeks after neoadjuvanttreatment	Day of hospital discharge from Surgery	Starting 1 month after surgery, every 3/6 months*
Items	(Vs)	(V_n)	(Vr)	(Vs)	(F1--x)
Informed consent	x				
Inclusion/exclusion	x				
Demography[i]	x				
Medical history	x				
Vital sign (P, R, T, BP)	x	x	x	x	
Physical examination[ii]	x		x		x
Body weight	x	x	x	x	x
ECOG performance score	x				
CT thorax/abdominal	x[iii]		x		x[iv]
Endoscopic ultrasound	x		x		x
Upper GI endoscopy	x				
gastroscopy	x				x[v]
Histopathology report	x			x	
Blood and Tissue	x			x	

specimen[vi]					
ECG[vii]	x		x		
Pulmonary function	x		x		
Blood routine and biochemistry [viii]	x	x	x	x	
Laboratory Infection[ix]	x				
Laboratory Coagulation	x		x		
Pregnancy test (only women)	x				
Blood sample[x]	x			x	x
Adverse events and complications [xi]	x				
Concomitant medication[xii]	x				

* The first follow-up visit is performed 1 months after surgery. From then on, follow-up visits are carried out every 3 months (+/- 7days) in the first two years of follow-up and every 6 months (+/- 7days) from the third year after treatment until the end of follow-up (min. 3 years).

i. Demography includes sex, age, height, race, ethnicity, job category, allergy and so on
ii. Physical examination includes, but is not limited to, cardiovascular, gastrointestinal, hepatobiliary, respiratory, musculoskeletal, skin, neurological, genitourinary/renal and other organ systems.
iii. Not earlier than 14 days before date of enrollment, and it must be contrast-enhanced CT. If suspected to be T4b stage, multiregional lymph node metastases or distant metastases, PET-CT or endoscopic ultrasound (EUS) (selectable) is performed to ensure pre-treatment cTNM stage.
iv. Contrast-enhanced CT of Thorax/Abdomen is carried out regularly at follow-up visits. Further diagnostic investigations, including PET-CT, Upper endoscopy are performed only if suspected to be recurrence or metastasis at the discretion of the investigator/treating physician.
v. Gastroscopy is performed once a year.
vi. Representative blocks from the initial biopsy and the operative specimen will be requested from the reporting pathologists.
vii. ECG must be performed during screening and preoperative. Patients with a cardiac history should have echocardiography, further cardiac examinations can be performed if necessary to exclude contraindication.
viii. Blood routine includes hemoglobin, total red count, total white blood count, platelet count, and a differential white count including neutrophils, lymphocytes, monocytes, eosinophils and basophils. Biochemistry includes (but is not limited to) AST, ALT, total bilirubin, blood glucose, serum creatinine, sodium, potassium.

ix. HBV, HCV, HIV serological examinations Coagulation includes PT, PTT, and INR.
x. Two blood samples are collected for translational research before treatment, before surgery, 4 months after surgery and the time of recurrence or metastasis, respectively.
xi. The AE reporting period for this trial begins after first intake of medication within the study and until 8 months after enrollment. All adverse events have to be documented in the CRF.
xii. Concomitant medication must be available in the source data and don`t be captured in the CRF.

7.1 Case Selection

When admitted to hospital, the potential patients who are meeting all the inclusion criteria and are beyond any one of exclusion criteria are selected.

7.1.1 Assessment

The clinical examination results that got between hospital admission and study enrollment (usually 1 week) are determined as the baseline data. These data must include:

- General status: height, weight, ECOG performance score, ASA score;
- Peripheral venous blood: Hb, RBC, WBC, LYM, NEU, NEU%, PLT;
- Blood biochemical indexes: ALB, prealbumin, TBil, DBil, AST, ALT, Cr, BUN, BG, CRP, HbA1c, Glycated albumin;
- Serum tumor markers: CEA, SCC;
- Imaging examinations, including thoracoabdominal enhanced computed tomography, cervical ultrasonography, endoscopic ultrasonography (performed as possibly) and positron emission tomography (optional when necessary)
- Standard 12-lead electrocardiogram;
- Pulmonary function examination: FEV1, FVC.

7.1.2 Selection Application

Before enrollment in this study, the research assistant should fill in the **Eligibility Application Form** for patients that meet all the inclusion criteria and are beyond any one of exclusion criteria and then send it to the PI research team through e-mail or fax for reviewing whether the patients are eligible.

7.1.3 Eligibility Consulting

Contact Information and Working Hours of Research Committee:
Add: Research Committee of Esophageal Cancer Treatment, Zhongshan Hospital, Fudan University

Tel: 021-64041990 –2917

Contact Information:
Lijie Tan
Add: Department of Thoracic Surgery, Zhongshan Hospital, Fudan University, Fenglin Road 180, Xuhui District, Shanghai.
Code: 200032
Tel: 021-64041990 -2917
E-mail: tan.lijie@zs-hospital.sh.cn

Jun Yin
Add: Department of Thoracic Surgery, Zhongshan Hospital, Fudan University, Fenglin Road 180, Xuhui District, Shanghai.
Code: 200032
Tel: 021-64041990 -2917
E-mail: jun_yin@fudan.edu.cn

7.1.4 Attentions

1) The application and confirmation of eligibility should be completed preoperatively.
2) Eligibility Application Form must be completely filled; otherwise, it will not be accepted.
3) After accredited by the Research Committe, the case should be numbered (Baseline Number, BN), and the eligibility confirmation notice should be emailed to the applicant.
4) The research assistant is responsible for the eligibility confirmation notice keeping.
5) Once selected for registration, the content of the eligibility application form will be entered into the database, and the eligibility is not allowed to be artificially canceled (the relevant information cannot be deleted from the database), unless the patient declines the information to be used in this study.
6) The data center will reject any repeatedly registered information. If it happens, the first registered data will be used (first BN).
7) In case of repeat selection or incorrect registration, the research assistant should contact the Research Committee and record it.

7.2 Written Informed Consent

The written informed consent is provided by the patient after comprehensively understanding of the trial.

7.3 Neoadjuvant immunotherapy

Patients received adebrelimab (administered intravenously at a dose of 20 mg

per kilogram of body weight every three weeks) on day 1 of a planned 21-day cycle, and two doses in total before surgery. Patients discontinued treatment if one of the following occurred: the patient made the decision to withdraw or there was unacceptable toxicity, disease progression according to RECIST 1.1 criteria, undercurrent illness, or changes in patient condition preventing further treatment at the discretion of the investigator. Safety and efficacy data were reviewed by an independent data monitoring committee.

7.4 Assessments during neoadjuvant therapy

The patients will be closely monitored for toxic effects of chemotherapy with the use of the CTCAE version 5.0. Vital sign, body weight, description of discomfort symptoms and standard laboratory tests (blood routine, blood biochemistry) will be obtained and recorded weekly before and during neoadjuvant therapy period, which assessed the toxicity of preoperative therapy. After 4 weeks of neoadjuvant therapy, a CT (or PET-CT) scan of thorax and abdomen and ultrasound of the neck will be performed to re-staging of the tumor.

7.5 Surgery Procedure

7.5.1 Surgery

After 4-8 weeks of neoadjuvant therapy, minimally invasive esophagectomy (MIE) will be performed. The procedure in details is referred in previous articles(*44-46*). To achieve an accurate ypTNM stage, the extent of lymphadenectomy demands a radical dissection. Dissected lymph nodes were classified according to lymph node stations adopted by the Japanese Classification(*47*). The dissected nodes in thoracic cavity should include the upper paraesophageal (no.105), paratracheal (no.106r and 106tb), subcarinal (no.107), middle paraesophageal (no.108), bilateral hilar lymph nodes (no.109), lower paraesophageal (no.110), posterior mediastinal lymph nodes (no.111), and diaphragmatic (no.112) ones. The dissected abdominal nodes should include the nodes lateral to the paracardia, lesser curvature, greater curvature, left gastric, common hepatic, splenic, and celiac stations. If neoplasm is located at upper mediastinum, cervical nodes in the cervical paraesophageal (no.101) and supraclavicular regions (no.104) should be dissected.

7.5.2 Observations during operation

The research assistant after operation should record the specific items:

- Name of doctor in charge;
- Operation starting time (min), Operation finishing time (min);
- Operation type, extent of lymphadenectomy, reconstruction method;
- Incision length (cm), number of trocars;
- Whether the MIE is transferred to open surgery and reasons;
- Estimated blood loss during operation (ml);

- Volume of blood transfusion (defined as transfusion of red cell suspension or plasma, ml);
- Tumor position;
- Tumor size;
- Invasion depth, distant metastasis (position);
- Proximal resection margin length (mm), distal resection margin length (mm), radical degree (R0/R1);
- Intraoperative complications;
- Intraoperative death: regardless of any reason.

7.6 Postoperative Management

7.6.1 Fluid Infusion and Nutritional Support

Postoperative fluid infusion (including glucose, insulin, electrolytes, vitamins etc.) or nutritional support (enteral/parenteral) is performed according to the experience of the doctor in charge and clinical routines, which is not specified in this study.
After oral feeding, fluid infusion/ nutritional support should gradually reduce until stop.

7.6.2 Rehabilitation Management

Management of incision, chest tube, cervical drainage-tube, and abdominal cavity drainage-tube: following the clinical routines.
Recovery eating time and transition strategies of diet: following the clinical routines.

7.6.3 Discharge Standard

No postoperative complications, meeting “body temperature is less than 37°C”, “the pain can be tolerated”, and “starting oral intake for more than 2 days”, a patient can be arranged for discharge, which should be recorded in the CRF.

7.6.4 Postoperative Observation Items

Definition of postoperative “n days”: One day from 0:00 to 24:00. The time frame from the end of surgery to 24:00 of the surgery day is defined as “postoperative 0 day”; the next day from 0:00 to 24:00 is “postoperative 1 day”, and so on.
From postoperative 1 day to discharge day, the research assistant should timely record the items. The observation items include:

7.6.4.1 Pathological Results

- Surgical outcomes (R0/R1/R2);

- Histological type of primary lesion;
- Depth of esophageal wall invasion;
- Histological grade (G1/G2/G3/G4/GX);
- Lymphovascular invasion;
- Total number of retrieved lymph nodes, number of lymph nodes in each group, number of lymph node metastasis in each group, and the total number of lymph node metastasis;

7.6.4.2 Early postoperative complications:

Time frame: 30 days after operation (postoperative hospital stay ≤ 30 days) or operation to first discharge from hospital (postoperative hospital stay > 30 days). Observation items:

- Surgery-related complications: Wound complications (infection, effusion, dehiscence, poor healing, etc.), active bleeding, anastomotic stenosis, intestinal fistula, pancreatic fistula, chylous fistula, abscess formation, intestinal paralysis, intestinal obstruction, cholecystitis, pancreatitis, etc.
- System-related complications: Pneumonia, pleural effusion, pulmonary embolism, cardio-cerebrovascular complications (including thrombosis and embolism), deep venous thrombosis, urinary tract complications, catheter-related complications, etc.

Classification of Surgical Complications (Clavien-Dindo Classification）

- Grade I: Any deviation from the ordinary postoperative course without the need for pharmacological treatment or surgical, endoscopic, and radiological interventions. Acceptable therapeutic regimens are: drugs as antiemetics, antipyretics, analgesics, diuretics, electrolytes, and physiotherapy. This grade also includes wound infections opened at the bedside.
- Grade II: Requiring pharmacological treatment with drugs other than such allowed for grade I complications. Blood transfusions and total parenteral nutrition are also included.
- Grade III: Requiring surgical, endoscopic or radiological intervention
  - IIIa: Intervention not under general anesthesia
  - IIIb: Intervention under general anesthesia
- Grade IV: Life-threatening complication (including CNS complications)* requiring IC/ICU management
  - IVa: Single organ dysfunction (including dialysis)
  - IVb: Multiorgan dysfunction
- Grade V: Death of a patient

Suffix “d”: If the patient suffers from a complication at the time of discharge, the suffix “d” (for “disability”) is added to the respective grade of complication. This label indicates the need for a follow-up to evaluate the complication fully.

Brain hemorrhage, ischemic stroke, subarachnoidal bleeding, but excluding transient ischemic attacks.
IC: Intermediate care; ICU: Intensive care unit

7.6.4.3 Blood test items (Postoperative day 1, 3, 6, 10)

- Peripheral venous blood: Hb, RBC, WBC, LYM, NEU, NEU%, PLT;
- Blood biochemical indexes: ALB, prealbumin, TBil, DBil, AST, ALT, Cr, BUN, BG, CRP;

7.6.4.4 Postoperative rehabilitation evaluation items

- First ambulation time (hour);
- First anal exsufflation/ defecation time (hour);
- Time to full/semi-liquid food intake (hour);
- Daily highest body temperature (℃);
- Chest tube extubation time (hour), daily drainage volume (ml);
- Peritoneal drainage tube extubation time (hour), daily drainage volume (ml);
- Volume of blood transfusion (defined as transfusion of red cell suspension or plasma, ml));
- Hospitalization time after operation (d).

7.7 Follow-up

7.7.1 Follow-up period and attentions

- Research center should arrange a specialist to carry out the follow-up 30 days after operation (postoperative hospital stay ≤ 30 days) or operation to first discharge from hospital (postoperative hospital stay > 30 days).
- In this study, it is recommended that the follow-up examination should be conducted in the research center, and the specialist should record the results.
- The specialist should evaluate and record the recovery situation of patient through analyzing the examination results.
- If the patient refuses the follow-up according to the protocol, it will be recorded as a case of “lost to follow-up”, and analyzed together with the cases meeting the study criteria at the end of the study.

7.7.2 Examination Items

7.7.2.1 Physical Examination

The doctor in charge should conduct a physical examination at the time of follow-up, and be aware of the vital signs, systemic superficial lymph nodes and

so on.

7.7.2.2 Blood test items:

- Peripheral venous blood: Hb, RBC, WBC, LYM, NEU, NEU%, PLT；
- Blood biochemical indexes: ALB, prealbumin, TBil, DBil, AST, ALT, Cr, BUN, BG；
- Serum tumor markers: CEA, SCC；

7.7.2.3 Imaging Examination:

- Thoracic enhanced CT (slice thickness of 5mm or less. If patients are allergic to the contrast agent, plain CT is permitted);
- Gastroscopy, ultrasonography, whole-body bone scan, PET-CT, etc., when attending doctors evaluate that it's necessary.

7.8 Adjuvant Therapy

- According to the postoperative pathological results, R0 resection cases with Stage II/III/IV are recommended to receive adjuvant chemotherapy or immunotherapy. The regimen is not specified in this study.
- For non-R0 resection cases, radiotherapy will be recommended in addition.
- For relapse cases after surgical resection, the follow-up treatment protocols are not specified in this study.

7.9 Assessments during the follow-up

The first follow-up visit will be performed 1 month after surgery. From then on, follow-up visits are carried out every 3 months till the end of follow-up. For all patients, follow-up assessment is performed until the end of the trial or death. The end of the trial will be 2 years after the study treatment of the last patient started. The detailed examination items include standard laboratory tests (blood routine, tumor biomarker), a CT scan of thorax, an ultrasound of the neck and abdomen.

7.10 Definitions

7.10.1 ECOG Performance Status

Developed by the Eastern Cooperative Oncology Group:

- 0: Fully active, able to carry on all pre-disease performance without

restriction.

- 1: Restricted in physically strenuous activity but ambulatory and able to carry out work of a light or sedentary nature, e.g., light housework, office work.
- 2: Ambulatory and capable of all selfcare but unable to carry out any work activities; up and about more than 50% of waking hours.
- 3: Capable of only limited selfcare; confined to bed or chair more than 50% of waking hours.
- 4: Completely disabled; cannot carry on any selfcare; totally confined to bed or chair.
- 5: Dead.

Patients at Grade 3, 4, and 5 are generally considered to be unsuitable for surgical treatment or immunotherapy.

7.10.2 ASA Classification

- ASA I: A normal healthy patient.
- ASA II: A patient with mild systemic disease.
- ASA III: A patient with severe systemic disease.
- ASA IV: A patient with severe systemic disease that is a constant threat to life.
- ASA V: A moribund patient who is not expected to survive without the operation.
- ASA VI: A declared brain-dead patient whose organs are being removed for donor purposes.

*The addition of "E" denotes Emergency surgery: (An emergency is defined as existing when delay in treatment of the patient would lead to a significant increase in the threat to life or body part)
Generally, ASA I/II patients are considered to be suitable for surgical treatment. ASA III patients are exposed to have some risks of anesthesia, and adequate preparation should be made before anesthesia. ASA IV patients are exposed to have high risks of anesthesia, and the perioperative mortality rate is very high even if the preoperative preparation is adequate. ASA V/VI patients are considered to be unsuitable for surgical treatment.

8 Exploratory immunologic studies

8.1 Cohort

Patients enrolled in this study will undergo the same laboratory correlate studies on tumor biopsy, resection specimen and serum samples as subsequently enrolled patients.

8.2 Tumor Tissue Samples

8.2.1 Collection of biopsy and surgically resected tumor tissue

All biopsy and surgically resected tumor samples were macroscopically reviewed by two experienced pathologists. Spatially separated localized tumors were collected within 30 min after esophagoscopy and surgery, and snap frozen in liquid nitrogen for subsequent processing following a Standard Operating Procedure. The tumor specimen was dissected by the pathologist with new and sterile scalpel blades on a clean surface to avoid contamination with other tissues. A cut fully bisecting the tumor, without compromising integrity of the whole specimen, was made.

8.2.2 Tumor tissue Handling, Transportation, Storage, and Processing

Tumors will be collected and snap frozen in liquid nitrogen for subsequent processing following a Standard Operating Procedure. Select a box that is large and thick enough to hold the samples, plus enough dry ice to keep the samples frozen during transit.

8.3 Blood Samples

8.3.1 Collection schedule

Blood samples will be drawn at the time points identified on the study calendar. Time points include: Day-0, before Day-22, before surgery and after surgery within 8 weeks.

8.3.2 Specimen handling, transportation, storage, processing

Peripheral blood will be collected at the time of biopsy and collected in streck tubes and processed per manufacturer's instructions. Viable peripheral blood will be stored in cryopreservation medium in liquid nitrogen.

8.4 Methods of Analysis

8.4.1 Immunohistochemistry

We determine by immunohistochemistry (PD-L1 IHC 22C3 pharmDx, Agilent Technologies, Carpinteria, CA) using the combined positive score (CPS) defined as the number of PD-L1 positive cells (including tumor cells, macrophages, and lymphocytes) divided by the total number of tumor cells, multiplied by 100.

8.4.2 Multiplex Immunofluorescent Staining

Tissue sections will be blocked with 3% hydrogen peroxide in TBST for 10 min

and staining with multiplex mIHC kit (Panovue, 10004100100), Briefly, the slides incubated with primary antibody for 60 min, then incubated using the HRP-polymer detection system for 10 min each step, before visualization using TSA 520 for another 10 min. Following this, antigen retrieval will be conducted again to prepare the slides for the next antibody. Using this TSA multiplex immunofluorescent staining method, all samples will be stained sequentially with the primary antibody.
All tissue sections that undergo multiplex fluorescent staining for each fluorophore will be imaged using the Polaris imaging system (PerkinElmer, Shanghai Kelin Institute) under the appropriate fluorescent filters for multispectral analysis. A whole slide scan of the multiplex tissue sections produce multispectral fluorescent images visualized in Phenochart (PerkinElmer, Shanghai Kelin Institute) and imaging at 200x magnification for further image analysis.

8.4.3 Whole exome sequencing

Genomic DNA and RNA of tumor tissue will be extracted using the QIAamp AllPrep DNA/RNA mini-Kit (Cat#80204, QIAGEN) according to the manufacturer's specifications including the steps of DNA/RNA separation, purification, and collection in columns. Genomic DNA of peripheral blood will be extracted using the QIAamp DNA mini-Kit (Cat#51304, QIAGEN) according to the manufacturer's specifications including the steps of DNA separation, purification, and collection in columns. The concentrations of DNA will be quantified using Qubit dsDNA BR Assay Kit (Cat#Q32850, Thermo Fisher Scientific) and the quality of the DNA was evaluated by agarose gel electrophoresis. The exome libraries will be constructed using the MGIEasy Exome Universal Library Prep Set (Cat#1000009657, MGI) according to the instructions. In brief, DNA is fragmented, followed by the steps of adaptor ligation at both ends, probe hybridization and PCR amplification. The concentrations of libraries will be quantified using Qubit dsDNA HS Assay Kit (Cat#Q32851, Thermo Fisher Scientific) and the quality of the libraries will be evaluated using the Agilent DNA 1000 Kit (Cat# 5067-1504, Agilent).

8.4.4 Bulk RNA sequencing

The concentrations of RNA will be quantified using Qubit RNA HS Assay Kit (Cat# Q32852, Thermo Fisher Scientific) The quality and quantity of RNA will be evaluated using the Agilent RNA 6000 pico Kit (Cat#5067-1513, Agilent). Library of RNA sample will be prepared using MGI rRNA removal kit (Cat#1000005953, MGI) and MGIEasy RNA Library Prep Set (Cat#1000006383, MGI) according to the manufacturer's protocol. The concentrations of libraries will be quantified using Qubit dsDNA HS Assay Kit (Cat# Q32851, Thermo Fisher Scientific) and the quality of the libraries will be evaluated using the Agilent DNA 1000 Kit (Cat#5067-1504, Agilent). Libraries

will be sequenced on a MGISEQ2000 sequencer (MGI, China) with 100 bp paired-end reads. Paired-end reads will be first processed to remove adapters and low-quality sequences. Reads will be then aligned to the GRCh37 reference genome using STAR v2.5.1b58 and duplicate reads will be filtered using Picard Tools.

8.4.5 TCR sequencing

Multiplex PCR will be designed to amplify CDR3 regions of the rearranged TCRβ chain (TRB) from genomic DNA. The enriched TRB products will be cyclized into ssDNA libraries by MGIeasy Circulation Kit, and then will be sequenced on the MGI2000 sequencer with 100bp paired end reads.

9 Adverse Events

9.1 General

The descriptions and grading scales listed in the revised National Cancer Institute Common Terminology Criteria for Adverse Events (NCI-CTCAE) Version 5.0 will be adopted in this study. Adverse event reporting can be found at http://ctep.cancer.gov/reporting/ctc.html.

All adverse events that occur after the time of first dose of study medication to 90 days after the last dose must be monitored and documented. Subjects continuing to experience toxicity after discontinuation of the study drug may be contacted for additional assessments until the toxicity has resolved or is deemed irreversible. Any adverse event experienced during additional preoperative treatment or after the surgical procedure that the investigator feels is related to study treatment will be captured. All adverse events must be followed until resolved, return to baseline, improved to Grade ≤ 1, loss to follow-up, or death.

9.2 Definitions

9.2.1 Adverse Event (AE)

An AE is defined as any untoward medical occurrence in a patient or clinical investigation subject administered a medicinal product and does not necessarily have a causal relationship with this treatment. An AE can therefore be any unfavorable and unintended vital sign, symptom, laboratory test abnormality, or disease, including the following:

Worsening of pre-exiting medical conditions/diseases;

Any new adverse medical conditions. Medical conditions/diseases present before starting study treatment are only considered adverse events if they

worsen after starting study treatment (any procedures specified in the protocol);

Clinically significant laboratory abnormalities. Abnormal laboratory values or test results constitute adverse events only if they induce clinical signs or symptoms or require therapy.

9.2.2 Serious Adverse Event (SAE)

- A serious AE (SAE) is any untoward medical occurrence that:
- results in death
- is life-threatening (defined as when the subjects are at immediate risk of death at the time of the event);
- results in hospitalization or prolongation of existing hospitalization
- results in persistent or significant disability/incapacity
- is a congenital anomalies or birth defect
- is other important medical events that may not be immediately life-threatening or result in death or hospitalization but, based upon appropriate medical and scientific judgment, may jeopardize the subject or may require intervention [i.e. medical, surgical] to prevent one of the other serious outcomes listed in the definition above.)

Suspected transmission of an infectious agent (ie, any organism, virus or infectious particle, pathogenic or non-pathogenic) via the study drug is an SAE. Events related to pregnancy, overdose, cancer, and potential drug induced liver injury (DILI) are not always serious by regulatory definition, but these events must be handled as SAEs and then reported (see section 11.5, 11.6, 11.7).
The following hospitalizations are not considered SAEs of this study (unrelated to the worsening of AEs):

- due to a pre-existing disease without new AE and aggravation of a pre-existing condition (e.g., hospitalization for laboratory abnormalities that have persisted from before the trial to the present);
- for management reasons (e.g., routine health assessment);
- elective treatment, surgery, planned prior to signing consent;
- planned medical treatments or surgery that should be documented throughout the study protocol and/or in the subject's individual baseline information;
- for using of blood products

9.2.3 Unexpected adverse event

Any event of which the nature, severity, specificity, or result are inconsistent with the descriptions listed in the Investigator’s Brochure and (or) package insert, is considered “unexpected”.

9.2.4 Expected (known) adverse event

An expected adverse event is a common AE that have been observed in former studies and reported in the Investigator's Brochure. Any expected AE should be collected in a systematic and standardized manner, and the methods for determining shall be described.

9.2.5 Relationship

The clinical physician should comprehensively determine the relationship between adverse events and the serious adverse events according to the five-level classification as follows:
Definitely-related: An adverse event which has a timely relationship to the administration of the investigational drug/agent, follows a known pattern of response, for which no alternative cause is present.

Possibly-related: An adverse event, which has a timely relationship to the administration of the investigational drug/agent, follows a known pattern of response, but for which a potential alternative cause may be present.

Unlikely-related: An adverse event which does not have a timely relationship to the administration of the investigational drug/agent, follows no known pattern of response, does not reappear or worsen after re-administration of the investigational drug/agent (if applicable), and for which there is evidence that it is related to a cause other than the investigational drug/agent.

Unrelated: An adverse event, for which there is evidence that it is definitely related to a cause other than the investigational drug/agent. In general, there is no timely relationship to the administration of the investigational drug/agent, or if there is a timely relationship, the event does not follow a known pattern of response, and there is an alternative cause.

Indeterminable: An adverse event, which has a timely relationship to the administration of the investigational drug/agent, follows no known pattern of response, but a potential alternative cause does not exist.

9.3 Serious Adverse Event Collection and Reporting

All SAEs, whether related to study drug or not, are collected since the consent form is signed until the completion of the safety follow-up.

The investigators must complete "NMPA Serious Adverse Event Report Form" immediately, sign and date it when an SAE occurs. Symptoms, severity, causality, occurrence time, duration of treatment and outcomes of SAEs should be documented in SAE report. If the investigators believe that an SAE is

potentially related to the study drug, the relationship should be detailed in the narrative section of the SAE report form.

All SAEs, whether related or unrelated to adebrelimab and all pregnancies must be reported to the sponsor (by the investigator or designee) within 24 hours. The email address for SAE reporting is: hengrui_drug_safety@hrglobe.cn.

If the intensity of the ongoing SAE or its relationship to the study treatment changes, a follow-up report should be submitted immediately. If an error is found in a previously reported SAE, the SAE may be modified, withdrawn or downgraded in subsequent reports and reported in accordance with SAE reporting procedures.

SAEs must be recorded on the SAE Report Form.
If only limited information is initially available, follow-up reports are required. (Note: Follow-up SAE reports should include the same investigator term(s) initially reported.) If an ongoing SAE changes in its intensity or relationship to study drug or if new information becomes available, a follow-up SAE report should be sent within 24 hours. All SAEs should be followed to resolution or stabilization.

9.4 Non-serious Adverse Events and Reporting

A non-serious adverse event is an AE not classified as serious. All non-serious adverse events (not only those deemed to be treatment-related) should be collected continuously during the treatment period and for a minimum of 90 days following the last dose of study treatment.
Non-serious AEs should be followed to resolution or stabilization, or reported as SAEs if they become serious. All identified non-serious AEs must be recorded and described on the non-serious AE page of the CRF (paper or electronic).

9.4.1 Laboratory Test Abnormalities

Completion of supplemental CRFs may be requested for AEs and/or laboratory abnormalities, including but not limited to abnormal hepatic enzymes are reported/identified during the study.
Serious AE CRF page or SAE Report Form (paper or electronic) as appropriate:

- Any laboratory test result that is clinically significant or meets the definition of an SAE;
- Any laboratory abnormality that required the subject to have study drug discontinued;
- Any laboratory abnormality that required the subject to receive specific corrective therapy.

It is expected that wherever possible, the clinical, rather than the laboratory

term would be used by the reporting investigator (ie, anemia versus low hemoglobin value).

9.5 Pregnancy

Any female subject who becomes pregnant during the clinical study must exit the group. Any pregnancy that occurs in a partner of male subject, he is admitted to continue the study. The investigators must fill out the "Pregnancy Report/Follow-up Form for Hengrui's Clinical Study" and report to the sponsor in 24 h after awareness of pregnancy, and report to the ethics committee promptly.

The outcome of the pregnancy should be followed up until 1 month after delivery and reported to the sponsor. Pregnancy outcome including stillbirth, spontaneous abortion and fetal malformation are considered SAEs and need to be reported based on requirements.

9.6 Overdose

An overdose is defined as the accidental or intentional administration of any dose of a product that is considered both excessive and medically important. Simple drug overdose event is not regarded as an SAE, but should be documented as a protocol deviation. If causing AE/SAE, then this will be recorded separately.

9.7 Other Safety Considerations

Any significant worsening noted during interim, such as progressive disease, or final physical examinations, electrocardiograms, x-rays, and any other potential safety assessments, whether these procedures are required by the protocol, should also be recorded as a non-serious or serious AE, and reported accordingly.

10 Data Management

All information will be collected on study-specific case report forms by the study staff.

All study process records and source documents must be retained for regulated time required by applicable guidelines, or specified by Hengrui Pharmaceuticals. By the time the study is completed, the case report form of the subject must be reviewed and locked. The completed forms will then be forwarded for central review and inclusion in the study dataset with relevant source documentation as outlined in the case report forms. The data submission schedule is as follows:

At the time of registration:

- Registration Form

- Informed Consent Form (signed by the subject)
- Eligibility Checklist
- Source documents related to eligibility and randomization

Within 2 weeks after registration:
- Baseline study case report forms
- Pertinent source documents

Within 2 weeks after final dose of study medication:
- On study case report forms
- Pertinent source documents

10.1 Case Report Form (CRF)

10.1.1 Types and Submission Deadline

CRF used in this study and the submission deadline is as follows:
- Case screening: 7 days prior to surgery (time frame: 3 days)
- Enrolling: submitted to the data center one day prior to surgery
- Surgery: within 1 day after surgery
- Postoperation-Discharge: within 3 days after the first discharge
- Follow-up records: 7 days after each follow-up point

10.1.2 Transmission Methods

Paper CRF and web-based eCRF form are used for data submission.

10.1.3 Amendment

After the start of the study, if the CRF is found lack of necessary data items or unclear items, under the premises of ensuring the amendment of the CRF does not cause medical and economic burden and increased risks to the selected patients, the CRF can be modified after the Research Committee adopt it through discussing at the meeting. If the amendment of the CRF does not require to modify the study protocol, this study protocol will not be modified. That whether it is necessary to submit a report or lodge an application to research center's IRB for the CRF amendment should follow the provisions of various centers.

10.2 Meetings

All participants of study will hold a meeting after the site enrolled a subject. The other members in the trial will attend as appropriate, including study coordinator, data manager, study nurse, associate researcher, collaborator (if applicable), and statistician.

During these meetings, issues related to the expected enrollment rate, characteristics of participants, retention of participants, compliance with agreements (potential or actual violations), validity and integrity of data, toxicity, collection and transfer of serum samples to laboratories, and progress of target data will be discussed.

10.3 Monitoring

In order to study whether the implementation follows the protocol safely, to study whether to collect the data correctly, monthly monitoring should be implemented during the period of selection of cases in principle. The monitoring is based on the hospital visit to compare the difference between and the original data and data submitted.
The periodic data report completed by the data center should be submitted to the Research Committee, the Research Responsible Person and Efficacy and Safety Evaluation Committee, and should be discussed and analyzed in accordance with relevant monitoring provisions. The regular monitoring is to aim at feedback, improving the scientific, ethical nature of the study rather than trying to expose study or hospital issues. The Research Committee, the Research Responsible Person, and the person in charge of research participating hospitals should strive to improve and to avoid the problems pointed out in the regular monitoring reports.

Evaluation of research safety, the quality of data and whether the study is complete and reliable will be monitored continuously through day 90 following the last dose of adebrelimab. The evaluations will be conducted under the direction of the authorized representatives (CRA auditors) and the study statistician.

10.3.1 Monitoring items

- Data collection completion status: Selected registration number (cumulative/different time of period)
- Eligibility: Ineligible patients/potentially ineligible patients
- Different end of treatment, the reasons for suspension/end (different hospitals) in the study protocol
- Background factors, pre-treatment report factors, post-treatment report factors when selected for registration
- Severe adverse events
- Adverse events/adverse reactions
- Proportion of conversion to open surgery
- Protocol deviation
- Progress and safety of the study, other issues

10.3.2 Acceptable range of adverse events

Based on the qualification of the research centers in this study, in general, treatment-related death and life-threatening complications caused by surgeries do not happen basically; the percent of more than 3% is considered unacceptable. If treatment-related death is suspected or non-hematologic Grade 4 toxicity having a causal relationship with the surgery is determined, adverse events on each patient should be respectively reported to the Efficacy and Safety Evaluation Committee. If the number of treatment-related deaths or the number of patients with determined non-hematologic Grade 4 toxicity having a causal relationship with the surgery is up to 4, the final incidence proportion of adverse events will be apparently more than 3%, and therefore the inclusion of patients must be immediately suspended. Whether the study can continue to proceed should be determined until reviewed by the PI Efficacy and Safety Evaluation Committee.

10.3.3 Deviation/Violation of study protocol

Surgical resection, clinical examinations, or toxicity, efficacy evaluation and so on failing to be conducted in accordance with the study protocol are the deviation of the study protocol. When the monitoring is carried out, deviations developed by the Data Center and Research Committee in advance (allowed to after the start of the study in special circumstances) beyond the acceptable range specified in each study center should be included in the monitoring report in the form of "cases of deviation possibility", and divided into any arbitrary one of the following after discussed by the Research Committee:

10.3.3.1 Violation

Clinically inappropriate, a deviation at least complying with one of the following items specified in the protocol is called "violation"

- Affecting the study endpoint evaluation
- The responsibility lays the doctor in charge/hospital
- Intentional or systematic
- Significant danger or the degree of deviation
- Papers should record content violation in principle.

10.3.3.2 Acceptable deviation

- The acceptable deviation represents the acceptable range of each item set by the Research Representative/Committee and the data center before or after the beginning of the study.
- If it is within an acceptable range of deviation set in advance, no record is required in the monitoring report.

10.3.3.3 Deviation

- Items that do not comply with “Violation” or with "Acceptable deviation” are deviation items.
- Specific deviations that occur several times should be recorded as much as possible when the paper is published.
- When the monitoring report is discussed, the deviation should be classified as the following:
  - Deviated from undesired results: should be reduced
  - Deviation (inevitable): not to be actively reduced
  - Deviation (clinically appropriate): positive affirmation of the judgment by the doctor in charge/ hospital

11 Provisions of Adverse Events

The evaluation in this study refers to CTCAE v5.0 and “Accordion Severity Grading System”.

11.1 Evaluation

- Evaluation of adverse event/adverse reaction comprehensively refers to the [Accordion Severity Grading System] and [CTCAE v5.0].
- Adverse events are graded according to the content that is the nearest Grade 0 ~ 4 definition.

For treatment-related death, death adverse events are classified as Grade 5 in the original CTCAE.

- Toxicity items specified in the, Grade, and the discovery date of Grade should be recorded in the treatment process report. For other toxicity items observed, observed Grade 3 toxicity items are only recorded the freedom registration column of the treatment process report, as well as Grade and the discovery date of Grade. The grade recorded in the treatment process report must be recorded in the case.
- CTCAE v5.0, the so-called “Adverse Event”, “all observed, unexpected bad signs, symptoms and diseases (abnormal value of clinical examination are also included) in the treatment or disposal, regardless of a causal relationship with the treatment or intervention. So it can be divided into two types based on whether there is a causal relationship or not.
- Therefore, even if events that “obviously caused by primary disease (cancer)” or caused by supportive therapy or combination therapy rather than the study regimen treatment (protocol treatment) are defined as “adverse events”.
- For adverse event data collection strategy, the following principle should be complied with in this study: Adverse events within 30 days from the last

treatment day of the study regimen or hospitalization before first discharge (postoperative hospital stay > 30 days) (protocol treatment) should be collected entirely, regardless of the presence or absence of a causal relationship. (When adverse events are reported, the causality and classification of adverse events are separately discussed)

11.2 Reporting

- When "severe adverse events" or "unexpected adverse events" occur, the Research Responsible Person of research center should report to the Research Committee/PI (Lijie Tan). Before the start of the study, the Research Committee should send the report template to research center in advance. When "severe adverse events" or "unexpected adverse events" occur, the Research Responsible Person of research center should report them to the Research Committee/PI (Lijie Tan).
- Adverse events based on the relevant laws and regulations should be reported to the province (city) Health Authority at the location of research center. Severe adverse events based on clinical research-related ethical guidelines should be reported to the person in overall charge of the medical institution. The appropriate reporting procedures should be completed in accordance with the relevant provisions of all medical institutions at the same time. The person in charge of each center should hold obligations and responsibility for the emergency treatment of patients with any degree of adverse events to ensure patient safety.

11.2.1 Adverse Events with Reporting Obligations

11.2.1.1 Adverse Events with Emergency Reporting Obligations

Any of the following adverse events is the object that any adverse event should be reported urgently to:

- All patients died during the course of treatment or within 30 days from the last treatment day, regardless of the presence or absence of a causal relationship with the study regimen treatment. If cases are withdrawn of treatment, even if the latter treatment has begun, those patients also belong to emergent reporting objects, as long as within 30 days from the last treatment day or during hospitalization (hospital stay > 30 days). (day 0 is the final treatment day and 30 days is starting from the next day)
- Those patients with unexpected Grade 4 non-hematologic toxicity (CTCAE v5.0 adverse events other than the blood/bone marrow group), having a causality with the treatment (any of definite, probable, possible) are also emergent reporting objects.

11.2.1.2 Adverse Events with Regular Reporting Obligations

Any one of the following adverse events is a regular reporting object:

- After 31 days from the last treatment day, death that cannot rule out the causal relationship with treatments, including suspected treatment-related death; death due to apparent primary disease is excluded.
- Expected Grade 4 non-hematologic toxicity (CTCAE v5.0 adverse events other than the blood/bone marrow group).
- Unexpected Grade 3 adverse events: Grade 3 adverse events are not recorded in the “expected adverse events”.
- Other significant medical events: adverse events that the study group deems are found to bring essential and potentially permanent, significant impact on their offspring (except for MDS myelodysplastic syndrome, and secondary cancer).

Adverse events among above, determined to have a causal relationship (any of definite, probable, and possible) with the study regime are regular reporting objects.

11.2.2 Reporting procedure

11.2.2.1 Emergency reporting

- When emergent adverse events of emergency study reporting objects happened, the doctor in charge will quickly report it to the Research Responsible Person of the research participating hospitals. Where no contact can be gotten with the Research Responsible Person of the hospital, the coordinator, or the doctor in charge of the hospital must perform the responsibility instead.
- First Reporting: Within 72 hours after the occurrence of adverse events, the Research Responsible Person of the hospital should complete the “AE/AR/ADR first emergency report” and send it to the Research Committee by FAX and telephone.
- Second Reporting: The Research Responsible Person of each research participating hospital completes the “AE/AR/ADR Report” and a more detailed case information report (A4 format), and then fax the two reports to the Research Committee within 15 days after the occurrence of adverse events. If any autopsy examination, the autopsy result report should be submitted to the Research Committee.

11.2.2.2 General reports

The Research Responsible Person of each research participating hospital completes the “AE/AR/ADR report”, and then fax it to the Research Committee

within 15 days after the occurrence of adverse events.

11.3 Responsibilities and Obligations

11.3.1 Judgment of Study Discontinuation and Necessity for Sending an Emergency Notice to the Hospital

After the receipt of the report of the Research Responsible Person of the research participating hospital, the Research Committee reply to the Research Responsible Person of the unit for confirmation and negotiation, and then they jointly determine the urgency, importance, and influence of reporting events; if necessary, they temporarily stop the study, and contact with all research participating hospitals to take emergency notification countermeasures. According to the severity of urgency, data center and research participating hospitals can be contacted by telephone or instrument FAX as soon as possible after the initial contact by phone.

11.3.2 Report to PI Efficacy and Safety Evaluation Committee

After notifying, discussing and clarifying the adverse events in line with adverse events with reporting obligations in the emergency reports or regular reports to the Research Responsible Person of research participating units, the Research Committee should submit a report to the Efficacy and Safety Evaluation Committee within 3 days after the occurrence of adverse events and request a review that whether the reason analysis of and solution to the adverse events by the Research Responsible Person are appropriate.

At that time, “AE/AR/ADR First Emergency Report” and “AE/AR/ADR Report” submitted by the research participating hospital should include the discussion results and countermeasures of the Research Committee/Research Responsible Person (including the judgment of research continue/discontinue). For death within 30 days, treatment-related death among death after 31 days and expected Grade 4 non-hematologic toxicity, not only the course of individual patient is included, but also consideration given to that whether the frequency of occurrence falls within the expected range are included. If the frequency of occurrence exceeds the expected range, it should be faithfully recorded in the “II classification of adverse events-others” of “AE/AR/ADR Report”.

11.3.3 Discussion of Adverse Events under Regularly Monitoring

During the regular monitoring, the Research Committee/Research Responsible Person should carefully discuss, study adverse events in the monitoring report submitted by the research data center to confirm no missing report by each research participating hospital. The existence or inexistence of under-reporting

adverse events should be clearly documented in the discussion results of [regularly monitoring report] of the Research Committee.

11.4 Review of Efficacy and Safety Evaluation Committee

The Efficacy and Safety Evaluation Committee reviews and discusses the report in accordance with the procedures recorded in the Clinical Safety Information Management Guideline, and raises the recommendations in written form for the Research Responsible Person, including whether to continue to enroll the study objects or whether to need to modify the study protocol.

12 Ethical considerations

12.1 Responsibilities of investigators

The investigators are responsible for the implementation of this study in its center. The investigators will ensure the implementation of this study in accordance with the study protocol and in compliance with the Declaration of Helsinki, as well as domestic and international ethical guiding principles and applicable regulatory requirements. It is especially noted that the investigators must ensure that subjects giving the written informed consent can be enrolled in this study only.

12.2 Information and informed consent of subjects

An unconditional prerequisite for subjects to participate in this study is his/her written informed consent. The written informed consent of subjects participating in this study must be given before study-related activities are conducted.
Therefore, before obtaining informed consent, the investigators must provide sufficient information to the subjects. In order to obtain informed consent, the investigators will provide the information page of subjects, and the information required to comply with the applicable regulatory requirements. While providing written information, the investigators will orally inform the subjects of all the relevant circumstances of this study. In this process, the words used must be fully, easily understood by non-professionals, so that they can sign on the informed consent form according to their willingness based on subjects' fully understanding of this study.

The informed consent form must be signed and dated personally by the subjects and investigators. All subjects will be asked to sign on the informed consent form to prove that they agree to participate in the study. The signed informed consent form with signature and date should be kept in the research center where the investigators are located and must be properly safe kept for the future review at any time during the audit, inspection, inspection period.

Before participating in the study, the subjects should provide a copy of signed and dated informed consent form.

At any time, as long as access to important new information that may be related to the consent of the subjects, the investigators will revise the information pages and any other written information provided to the subjects and re-submit them to the IEC/IRB for review and raising a favorable opinion. The revised information agreed will be provided to each subject participating in the study. The researchers will explain the changes made to the previous version of ICF to the subjects.

12.3 Identity and Privacy of Subjects

After obtaining an informed consent form, each selected subject is assigned with subject number (Allocation Number, AN). This number will represent the identity of the subject in the whole study and the clinical research database for the study. The collected data of subjects in the study will be stored in the ID.

In the entire study, various safety measures to minimize leaking risks in the utilization process of personal information will be taken, including: (1) only the investigators were able to link the research data of the subjects with themselves through the identify table kept in the research center after authorized; (2) in the raw data auditing on-site conducted by the supervisors of this study, as well as relevant inspection and inspection visit by the supervision departments, the personnel engaging above activities may view the original medical information of subjects that will be kept strictly confidential.

Data collection, transmission, handling, and storage of subjects will comply with the data protection and privacy regulations. This corresponding information will be provided to the subjects, and the subjects were asked to provide their consent for the treatment procedures of above data in accordance with national regulations.

12.4 Independent Ethics Committee or Institutional Review Committee

Before beginning the study, the Research Center will be responsible for submitting the study protocol and relevant documents (informed consent form, subject information page, CRF, and other documents that may be required) to the Independent Ethics Committee (IEC)/ Institutional Review Board (IRB) to obtain their favorable opinion/approval. The favorable opinion/approval documents of the IEC/IRB will be archived in the research center folders of the investigators.

Before obtaining the written proof of favorable opinions/approval of the IEC/IRB, the investigators are forbidden to begin the study in the center. The IEC/IRB

will be asked to provide the written proof of the date of the favorable opinions/approval meeting and the written proof of the members presenting at the meeting and voting members. The IEC/IRB should provide the written proof of the favorable opinion/approval, recording the reviewed study, protocol version, and Informed Consent Form version. If possible, a copy of the minutes should also be obtained.

In the case of major revisions in this study, the amendment of the study protocol will be submitted to the IEC/ IRB prior to performing. In the course of the study, the relevant safety information will be submitted to the IEC/IRB in accordance with national regulations and requirements.

12.5 Supervisory Authority

The study protocol and any relevant documents (for example, the study protocol, the subject's informed consent form) will be submitted according to the Ethical Review Approach of Biomedical Research Involving Human Beings (Trial) (2007) and the applicable regulatory requirements of our country or will notify the ethical review guidance counseling organization of the provincial health administrative departments at the location of each research center.

13 Organizations and Responsibilities of Study

13.1 Research committee

- Being responsible for developing study protocol, auditing eligibility for inclusion, and guiding the interpretation of informed consent; being responsible for the collection of hazardous/adverse event reports, guiding the clinical diagnosis and treatment of such events, and the emergency intervention of serious adverse events.
- The PI of Research Committee: Lijie Tan (Department of Thoracic Surgery, Zhongshan Hospital, Fudan University). Add: Department of Thoracic Surgery, Zhongshan Hospital, Fudan University, Fenglin Road 180, Shanghai 200032, China; Tel: 86-21-64041990-2917; Fax: 86-21-64038477; E-mail: tan.lijie@zs-hospital.sh.cn.
- Research Representative: Jun Yin (Department of Thoracic Surgery, Zhongshan Hospital, Fudan University). Add: Department of Thoracic Surgery, Zhongshan Hospital, Fudan University, Fenglin Road 180, Shanghai 200032, China; Tel: 86-21-64041990-2917; Fax: 86-21-64038477 ; E-mail: jun_yin@fudan.edu.cn.

13.2 Efficacy and Safety Evaluation Committee

- Being responsible for the supervision, monitoring of the treatment safety, and therapeutic efficacy of this study.
- The PI of Efficacy and Safety Evaluation Committee: Lijie Tan (Department of Thoracic Surgery, Zhongshan Hospital, Fudan University)

13.3 Data Center

- Participating in the design of this study protocol, being responsible for data analysis, statistical interpretation, and issuing of statistical reports.
- Being responsible for the formulation and provision of CRFs and eCRF (web-based electronic case report forms) and management, storage of research data, and maintenance of database.
- Person in charge of Data Center: Qing Zhou (Zhongshan-BGI Precision Medical Center, Zhongshan Hospital, Fudan University, Shanghai 200032, China)
- The Second Person in Charge of Management of Study Data: CRO

13.4 Data and Safety Monitoring Board (DSMB)

- DSMB is responsible for the supervision of efficacy, the safety of this study, supervising of all aspects performed of the study, and licensing before the release of the validity of the study results.
- Person in Charge of DSMB: CRO

13.5 Independent Ethics Committee/Institutional Review Board (IEC/IRB)

- Being Responsible for evaluating this study in order to determine “whether to minimize risks that the subjects are exposed to” and “whether the risks that the subjects are exposed to are reasonable compared to expected benefits”.
- The independent Ethics Committee/Institutional Review Board (IEC/IRB) at the location of research center is responsible for the ethics review of all research participating units.

13.6 Principal Investigator Responsibilities

The Protocol Chair is responsible for performing the following tasks:

- Coordinating, developing, submitting, and obtaining approval for the protocol as well as its subsequent amendments.
- Assuring that the correct version of the protocol is used.
- Taking responsibility for the overall conduct of the study and for monitoring the progress of the study.
- Reviewing and ensuring reporting of Serious Adverse Events (SAE).
- Reviewing data from all sites.

14 General and statistical considerations

14.1 Analysis Sets

14.1.1 Modified Intention-To-Treat Set

The modified Intention-To-Treat Set (mITT) will include all recruited subjects who received at least 1 dose of study drug and have at least 1 post-baseline tumor assessment. mITT will be used for the supportive analyses.

14.1.2 Safety Analysis Set

The Safety Analysis Set (SS) will include all subjects who received at least 1 dose of study drug and have a safety record after drug administration. SS will be used for the safety analyses.

14.2 General Considerations

14.2.1 Reference Start Date, End Date and Study Day

- Reference start date is the first date of study treatment administration
- Reference end date is the last date of study treatment administration
- Study day = event/assessment date – reference start date + (event/assessment date ≥ reference start date)

14.2.2 Baseline

In general, baseline is defined as the last non-missing measurement taken prior to reference start date. In the case where the last non-missing measurement and the reference start date coincide, that measurement will be considered pre-baseline, but Adverse Events (AEs) and medications commencing on the reference start date will be considered post-baseline.

14.2.3 On-treatment phase

In general, on-treatment phase is defined as the period from first adebrelimab administration to within 30 days after surgery or 90 days after last dose of adebrelimab administration, which occurs latter.
On-treatment phase is primarily defined for safety analysis.

14.2.4 Definition and Use of Visit Windows

All the by-visit analysis will use the original visits being recorded in the eCRF. No derivation of visit will be performed.

14.2.5 Repeated or Unscheduled Assessments of Safety Parameters

In general, all the by-visit summaries will only present the data being recorded in scheduled visits. Measurements recorded in unscheduled visits will not be included in the by-visit summaries, but will contribute to determine the best/worst case value if required (i.e. shift table).
If there are multiple values for the same test before the initiation of study treatment, the last available assessment will be used.
Listings will include all the scheduled and unscheduled data.

14.3 Statistical Considerations

14.3.1 Missing Date or Incomplete Date

In general, the incomplete date will be imputed following the below rule unless otherwise specified if it has impact on the statistical analysis:

- Will not impute if completely missing.

14.3.1.1 Missing or Incomplete Date Information of Study Treatment

14.3.1.1.1 Missing or Incomplete Start Dates

- Only day is missing: impute 1 to the day part.
- Month and day are missing: impute to Jan 01.
- Completely missing date will not be imputed.

14.3.1.1.2 Missing or Incomplete End Dates

- Only day is missing: impute to the last day of the month.
- Month and day are missing: impute to Dec 31.
- Completely missing date: impute to the cutoff date as 28 Jan 2021.

In case the imputed start date is later than the stop date, then the start date will be imputed using the end date.

14.3.1.2 Missing or Incomplete Date Information for New Antitumor Therapies

- Only day is missing: impute 1 to the day part.
- Month and day are missing: impute to Jan 01.
- Completely missing date will not be imputed.

In case the imputed new antitumor therapy date is earlier than the next day of the last date of study treatment administration, then the new antitumor therapy date will be imputed using the next day of the last date of study treatment administration.

14.3.1.3 Missing or Incomplete Date Information for the First Pathology Diagnosis

- Only day is missing: impute 1 to the day part.
- Month and day are missing: impute to Jan 01.
- Completely missing date will not be imputed.

Imputed date should not be earlier than the birth date.

14.3.1.4 Missing or Incomplete Date Information for Adverse Events and Concomitant Medications

14.3.1.4.1 Missing or Incomplete Start Dates

- Missing Day Only
  - If the known parts of the incomplete date is the same as the corresponding parts of the first date of study treatment administration, then impute to the date of first study treatment administration.
  - Otherwise impute 1 to the day part.
- Missing Month and Day
  - If the year of the incomplete date is the same as the year of the first date of study treatment administration, then impute to the date of first study treatment administration.
  - Otherwise impute to Jan 1.
- Completely missing date will not be imputed.

14.3.1.4.2 Missing or Incomplete End Dates

- Only day is missing: impute to the last day of the month. If the imputed date is later than the death date, then use the death date to impute.
- Month and day are missing: impute to Dec 31. If the imputed date is later than the death date, then use the death date to impute.
- Completely missing date will not be imputed.

In case the imputed start date is later than the end date, then the start date will be imputed using the end date.

14.3.1.5 Missing or Incomplete death date

- Only day is missing: impute 1 to the day part.
- Month and day are missing: impute to Jan 01.
- If completely missing: impute to the next day of last date known to be alive.

In case the imputed date is earlier than the next day of last date known to be alive, then the death date will be imputed using the next day of last date known to be alive.

14.3.1.6 Missing or Incomplete PD date

- Missing Day Only
  - If the known parts of the incomplete date is the same as the corresponding parts of the last non-PD tumor assessment date, then impute to the next day of the last non-PD tumor assessment date.
  - Otherwise impute 1 to the day part.
- Missing Month and Day
  - If the year of the incomplete date is the same as the year of the last non-PD tumor assessment date, then impute to the next day of the last non-PD tumor assessment date.
  - Otherwise impute to Jan 1.
- Completely missing date will not be imputed, the subjects will be considered to be censored.

In case the imputed PD date is later than the death date, then the PD date will be imputed using the death date.

14.3.2 Character Values of Clinical Laboratory Tests

If the reported value for a clinical laboratory test is in form of a character string, then the numeric part of the string will be used for the analysis purpose.
If the result is reported as below-limit-of quantification (BLQ), then the lower boundary of the quantifiable assessment range will be used for the analysis purpose. However, the actual values as reported in the database will be presented in data listings.

14.3.3 Computing Methods and Reporting Conventions

All the statistical analysis will be performed with SAS® Version 9.4 or above.

14.3.3.1 Statistical Summary Conventions

For continuous variables, descriptive statistics including number of subjects with non-missing values, mean, standard deviation, median, minimum and maximum will be presented. For categorical variables, frequencies and percentages will be presented. Time-to-events data will be analyzed by Kaplan-Meier method and K-M plots will be presented if necessary.

14.3.3.2 General Reporting Conventions

The means and medians should have 1 more decimal place than the observed values, the standard deviations should have 2 additional decimal places than the observed values. Min and Max should have the same decimal place with the observed values.
p-values will be reported to 4 decimal places, the p-values less than 0.0001 will be reported as “<0.0001”, while the p-values greater than “0.9999” will be

reported as “>0.9999”.

14.3.4 Subgroups

Subgroup analysis will be conducted for efficacy by genetic profile or immune status, as exploratory analysis, which will be described in corresponding sections. It should be noted that the study is not designed to detect the difference in treatment within subgroups. If the subject number of the group is less than 10 or with other considerations, it may be pooled with others.
The following subgroups will be assessed:

- Age as categorical variable (< 65 years, ≥ 65 years)
- ECOG performance status (0, 1–2)
- Genetic profile (TMB, MSI)
- tumor immune microenvironment phenotypes
- Immune status of ESCC
- T cell receptor (TCR) clonotypes

15 Statistical analysis

15.1 Summary of Study Data

15.1.1 Subject Disposition

The number and percentage of subject in each analysis set (modified intention-to-treat set and safety analysis set) will be summarized by treatment group. The number and percentage of subjects who completed and discontinued treatment and study will be presented for each treatment group. Reasons for treatment and study discontinuation recorded on the EOS form of eCRF will be further summarized as number of subjects and percentage for each treatment group. In addition, the duration of subject in study will also be summarized.
Duration in study (months) = (Last day of known to be alive – randomization date + 1)/30.4375
Data listing of analysis sets, as well as end of treatment and end of study will be provided.

15.1.2 Protocol Deviations

Protocol deviations will be summarized by the treatment group and overall and furthermore summarized by severity.
Protocol deviations will be listed.

15.1.3 Demographic and Baseline Characteristics

Demographic and baseline characteristics will be summarized by treatment

group and overall for mITT using the following information from the 'Demographic Characteristic' eCRF pages.

- Age as continuous variable
- Age as categorical variable (<65-year, ≥65-year)
- Gender (male, female)
- Baseline ECOG PS (0, 1, 2)
- Baseline height (cm)
- Baseline weight (kg)
- Baseline Body Mass Index (BMI) (kg/m^2) (BMI (kg/m^2) = weight (kg)/height $(m)^2$)
- Genetic profile (TMB, MSI)
- Tumor immune microenvironment phenotypes
- Immune status of ESCC
- T cell receptor (TCR) clonotypes

The continuous data will be summarized by using descriptive statistics, including mean, standard deviation, median, maximum and minimum values. Categorical data will be presented as frequencies and percentages.
Data listing for demographic information will be provided.

15.1.4 Tumor Diagnosis and treatment history

The tumor diagnosis and prior treatment information listed below will be summarized by treatment group and overall for mITT using the following information from the 'Tumor History' eCRF pages.

- Time since initial diagnosis to date of randomization (months), defined as (date of randomization- date of initial diagnosis)/ 30.4375
- Method of diagnosis (Surgery, Biopsy)
- Serous carcinoma histologic subtype (High grade, Low grade)
- Non-serous carcinoma histologic subtype (Well differentiated, Moderately differentiated, Poorly differentiated)
- Ascites (yes, no)
- Tumor location (Proximal third, middle third, distal third)
- Clinical disease stage (II, III, IV)
- Combordity (None, one or more)

Data listing for tumor diagnosis will be provided.

15.1.5 Tumor Treatment History

Tumor treatment history will be summarized by treatment group and overall for mITT using the following information from the 'Past History' eCRF pages.
Following information will be summarized as follows based on the number and percentage of patients with the following:

- At least one prior chemotherapy
- Previous treatment lines. If there are patients received multiple treatment

lines, summarize the highest line only

- Best response, which is derived from the highest treatment line
- At least one prior radiotherapy
- At least one prior anti-cancer surgery

Listing of prior chemotherapy history (including treatment lines, start date, end date, chemotherapy regimens, number of treatments, best response, progression date, treatment types), prior anti-cancer radiotherapy (including radiotherapy sites, start date, end date, overall radiotherapy dose, best response) and prior anti-cancer surgery (including surgery name and surgery date) will be provided.

15.1.6 Medical History

Medical history will be coded using the most current available version 22.0 of Medical Dictionary for Regulatory Activities (MedDRA) and will be summarized by treatment group and overall for mITT from the 'Past History' eCRF page. Medical history will be summarized as the number and percentage of patients by MedDRA preferred term (PT) as event category and MedDRA primary system organ class (SOC) as summary category. Each patient will be counted only once within each PT or SOC.
Data listing of medical history will be provided.

15.1.7 Concomitant Medications

The concomitant medications are collected under the 'Therapeutic Medication' eCRF pages.
Concomitant medications are medications, other than study medications, which started prior to first dose date of study treatment and continued on on-treatment period as well as those started during the on-treatment period.
Summary of concomitant medications will include the number and percentage of patients in each treatment group and overall for SS by Anatomical Therapeutic Chemical (ATC) Classification level 2 and preferred term.
Data listing of concomitant medications will be provided.

15.2 Efficacy Analyses

15.2.1 Analysis of Primary Efficacy Endpoints

mITT will be used as the primary analysis set for the primary efficacy analysis.
The primary endpoint is feasibility, which will be analyzed primarily based on mITT.
The 95% confidence interval of feasibility will be estimated by using Clopper-Pearson method.
Subgroup analysis will be performed for the baseline factors listed in section 14.3.4.

Data listing regarding response status will be provided for subjects in mITT.

15.2.1.1 Subgroup analysis

In addition, subgroup analysis will be performed for the baseline factors listed in section .3.4.

15.2.2 Analysis of Secondary Efficacy Endpoints

15.2.2.1 Overall survival

The analysis of Overall survival (OS) is based on mITT. OS is defined as from the date of recruitment to the date of death. If a subject lost to follow-up or withdrawal from study before death is observed, the subject will be censored at the last date known to be alive.
The Kaplan-Meier method will be used to estimate the distribution of RFS (including the median RFS) for each treatment group, the 95% confidence intervals of mRFS will be calculated by using the Brookmeyer and Crowley method. The corresponding Kaplan-Meier curves will also be presented. The Kaplan-Meier method will be used to estimate the distribution of RFS (including the median RFS) for each treatment group, the 95% confidence intervals of mRFS will be calculated by using the Brookmeyer and Crowley method. The corresponding Kaplan-Meier curves will also be presented.
The Kaplan-Meier method will be used to estimate the distribution of OS (including the median OS), the 95% confidence intervals of mRFS will be calculated by using the Brookmeyer and Crowley method. 1-year and 2-year OS rate and their corresponding log(-log) transformed 95% CIs will be calculated. The corresponding Kaplan-Meier curves will also be presented.
Subgroup analysis will be performed for the baseline factors listed in section .3.4.
OS relevant information will be listed for all subjects in the mITT.

15.2.2.2 Recurrence-free survival

The analysis of Recurrence-free survival (RFS) is based on mITT. If a subject lost to follow-up or withdrawal from study before death is observed, the subject will be censored at the last date known to be alive.
The Kaplan-Meier method will be used to estimate the distribution of RFS (including the median RFS) for each treatment group, the 95% confidence intervals of mRFS will be calculated by using the Brookmeyer and Crowley method. The corresponding Kaplan-Meier curves will also be presented.
Subgroup analysis will be performed for the baseline factors listed in section 14.3.4.
RFS relevant information will be listed for all subjects in the ITT.

15.2.2.3 Pathological complete response rate (pCR) and R0 resection rate

Pathological complete response rate (pCR) will be analyzed primarily based on mITT. pCR is defined as the proportion of subjects with complete pathological response.

R0 resection rate will be analyzed primarily based on mITT.

The 95% confidence interval of pCR and R0 resection rate will be estimated by using Clopper-Pearson method.

Data listing regarding response status will be provided for subjects in mITT.

15.3 Safety Analyses

All the safety analysis will be performed for SS except for otherwise specified. Safety endpoints include treatment-emergent adverse events (TEAE), clinical laboratory tests, vital signs, electrocardiogram (ECG) parameters, echocardiogram (ECHO) parameters and Eastern Cooperative Oncology Group performance status (ECOG PS).

15.3.1 Dosing and Extent of Exposure

The following items reflecting drug exposure will be summarized by treatment group for adebrelimab based on SS:

Adebrelimab:

- Duration of exposure (cycle) = (last dose date of adebrelimab – first dose date of adebrelimab + 1)/28
- Duration of exposure (day) = (last dose date of adebrelimab – first dose date of adebrelimab + 1)
- Actual duration of exposure (cycle) = [(last dose date of adebrelimab – first dose date of adebrelimab + 1) – sum of dose interruption days of adebrelimab]/7
- Total dose (mg) = sum of dose of adebrelimab per cycle
- Dose intensity (mg/kg/cycle) = Total dose (mg/kg)/ Duration of exposure (cycle)
- Relative dose intensity (%) = Dose intensity (mg/kg/cycle)*100/ planned dose per day (20 mg/kg/cycle)

In addition, number and percentage of subjects who did not experience dose reduction or experienced dose reduction for at least 1 time will be summarized for each treatment group. Similarly, number and percentage of subjects who did not experience drug interruption or experienced drug interruption for at least1 time drug interruption will be summarized for each treatment group.

15.3.2 Adverse Events

Adverse events (AE) will be coded using Version 22.0 or above of the Medical Dictionary for Regulatory Activities (MedDRA) and classified according to the

NCI-CTCAE criteria version 5.0. AE will be summarized by treatment group.

- An AE will be considered as a TEAE if it occurs or becomes worse in severity after the initiation of study treatment and within 30 days after surgery or 90 days after last adebrelimab administration, which occurs latter.

If the CTCAE grade of an AE is missing, it will be considered as grade 3 AE.

The TEAEs which being recorded as being "definitely related with", "highly-possibly related with" and "possibly related with" study drug in eCRF will be considered as drug related TEAE (TRAE). If the relationship to the study treatment is missing, it will be considered to be related to study treatment.

A high-level summary of the number of subjects with TEAEs will be presented by treatment group, including the number and percentage of subjects with:

- Any TEAEs
- TEAEs with CTCAE grade ≥ 3
- TEAE leading to dose reduction of study treatment
- TEAE leading to interruption of study treatment
- TEAE leading to permanent discontinuation of study treatment
- TEAE leading to death
- Serious TEAE (TESAE)
- Study treatment related TEAEs (TRAEs)
- TRAEs with CTCAE grade ≥ 3
- TRAEs leading to dose reduction of study treatment
- TRAEs leading to interruption of study treatment
- TRAEs leading to death
- Serious TRAEs

The number and percentage of subjects reporting TEAEs in each treatment group will be summarized overall, by system organ class (SOC) and preferred term (PT) and further be summarized by severity (overall and for CTCAE grade ≥ 3). A subject who reports more than 1 TEAEs with the same SOC/PT will only be counted once at the corresponding SOC/PT by using the most severe CTCAE grade.
In addition, TEAE with incidence rate ≥10% will also be summarized by PT.
Data listing will be provided.

15.3.2.1 Treatment-related TEAEs

The number and percentage of subjects reporting TRAEs in each treatment group will be summarized overall by SOC/PT and further be summarized by severity (overall and for CTCAE grade ≥ 3). Only the occurrence with the highest CTCAE grade and the closest relationship to the study treatment will

be counted at the corresponding SOC/PT level if a subject who reports more than 1 TRAEs with different CTCAE grade and relationship to the study treatment which could be coded to the same SOC/PT.
In addition, TRAEs with incidence rate ≥10% will also be summarized by PT.
Data listing of TRAEs will be provided with the information of subject ID, AE name, start/end date, severity, CTCAE grade, relationship to the study drug, outcome, SAE or not, the action taken for AE and whether it has led to the withdrawal from study.

15.3.2.2 Serious adverse events

SAE will be summarized in the similar way of TEAE. The number and percentage of subjects reporting SAEs in each treatment group will be summarized overall by SOC/PT and further be summarized by severity (overall and for CTCAE grade ≥ 3).
Treatment related SAEs will be summarized in the similar way of SAEs.
Data listing of SAE will be listed.

15.3.2.3 TEAE leading to study drug interruption, dose reduction and discontinuation

The number and percentage of subjects reporting TEAEs which lead to study drug interruption, dose reduction and drug discontinuation in each treatment group will be summarized overall by SOC/PT and further be summarized by severity (overall and for CTCAE grade ≥ 3). A subject who reports more than 1 TEAEs with the same SOC/PT will only be counted once at the corresponding SOC/PT by using the most severe CTCAE grade.

TRAEs which lead to study drug interruption, dose reduction and drug discontinuation in each treatment group will be summarized in the similar way of TEAEs.
Data listing of TEAEs leading to study drug interruption, dose reduction and drug discontinuation will be listed.

15.3.2.4 Death, Other Serious Adverse Events and Other Significant Adverse Events

The number and percentage of TEAEs and TRAEs leading to death will be summarized will be summarized by SOC/PT for each treatment group.
In addition, the time to the first occurrence of TEAEs leading to death will be provided.

15.3.3 Clinical Laboratory Evaluations

The clinical laboratory evaluation system and parameters listed below will be summarized if applicable.
Table 2 List of Laboratory Tests

Hematology	Hemoglobin, white blood cell (WBC) count, absolute neutrophil count (ANC) and platelet (PLT) count
Urinalysis	Urinary pH, urine protein, urine red blood cells and urine white blood cell
Biochemistry	Liver function tests (ALT, AST and TBIL), renal function tests (BUN and Cr) and electrolyte tests (serum potassium, sodium and chloride)
Coagulation	Prothrombin time

The abnormality for each laboratory parameter will be assessed by investigator. A shift table from baseline to the worst post-baseline assessment according to the investigator's evaluation for each parameter listed above (if applicable) will be presented by treatment group.

In addition, all the laboratory abnormality will be graded according to CTCAE criteria version 5.0, if applicable. A shift table from baseline to the worst on-treatment CTCAE grade for each parameter (if applicable) will be presented.

Data listing for the abnormal laboratory tests results as well as the stool blood assessment results will be presented.

15.3.4 Vital Sign

The results and their changes from baseline of each vital sign parameter, including height (cm), weight (kg), BSA (m^2), temperature (℃), heart rate (beats/min), breath (breaths/min), systolic blood pressure (mmHg) and diastolic blood pressure (mmHg), will be summarized by descriptive statistics for baseline and each post-baseline scheduled visits as well as corresponding changes from baseline (if applicable) by study treatment.
Data listing will be provided for the vital sign results.

15.3.5 ECG and Echocardiogram

The quantitative results and their changes from baseline of each ECG parameter, including heart rate (beats/min), QTc (ms) and LVEF (%), will be summarized by descriptive statistics for baseline (changes from baseline is not applicable) and each post-baseline scheduled visits by treatment group.
The abnormality of ECG and Echocardiogram will be assessed by investigator. A shift table from baseline to the worst post-baseline assessment according to the investigator's evaluation will be presented for ECG and Echocardiogram by treatment group.
Data listing will be provided for the ECG and Echocardiogram results.

15.3.6 Eastern Cooperative Oncology Group (ECOG) PS

The analysis of ECOG will be performed based on ITT. The ECOG PS will be

summarized as categorical variables for baseline and each post-baseline scheduled visit by treatment group. In addition, the shift table from baseline to the worst score will be provided.
Data listing will be provided for the ECOG PS.

16 Sample size determination

The primary endpoint of this trial was feasibility. Feasibility of neoadjuvant adebrelimab will be defined as patients proceed to surgery without extended treatment related delays. A treatment related delay will be considered “extended” if it is greater than 37 days following the initially planned surgery date. For feasibility, an adebrelimab toxicity of any grade, that in the judgment of the investigator or surgeon could adversely impact perioperative morbidity or mortality, should delay the planned operative date.
A Simon optimal two-stage design was used. 70% of feasibility was considered unacceptable, and a 90%of feasibility was considered promising. This design allows early study termination for excessive surgery delay. The probabilities of type I and type II errors were set at 0.05 and 0.2, respectively.
Six patients will be accrued to the first stage, and if five or more patients proceed to surgery without extended treatment related delays, 21 patients would be enrolled in the second stage. If more than 23 of the 27 patients proceed to surgery without extended treatment related delays, this regimen would be considered worthy of further testing.

The first six patients will also be considered as the safety run-In phase. The primary DLTs of concern for safety monitoring will be grade 3-4 toxicities including liver, GI, renal, pneumonitis and any other grade 3-4 toxicity that in the opinion of the investigator significantly interfered with the subjects’ optimal perioperative management. They will be monitored continuously for six patients in the run-in phase through day 90 following the last dose of adebrelimab (or day 30 post-operation, whichever is longer) for the last patient in the run-in. If three of the first six patients experience DLT, the study will be discontinued. Overall, the study will continue to the second stage if none or one of the 6 patients experience DLT in the run-in phase. If two patients experience grade 3-4 DLT, the study will be paused for a review and may or may not continue at the discretion of the investigators.

17 Appendix

17.1 List of abbreviations and definitions of terms

Abbreviation	Definition
AE	Adverse event
ALT	Alanine aminotransferase
ANC	Absolute neutrophil count
AST	Aspartate aminotransferase
ATC	Anatomical therapeutic chemical class
BLQ	Below level of quantification
BMI	Body mass index
BOR	Best overall response
BUN	Blood urea nitrogen
CR	Complete response
ECG	Electrocardiogram
ECHO	Echocardiogram
ECOG	Eastern cooperative oncology group
ESCC	Esophageal squamous cell carcinoma
eCRF	Electronic case report form
EOS	End of study
HRQoL	Health-related quality of life
MSI	
ITT	Intention-to-treat
LVEF	Left ventricular ejection fraction
MedDRA	Medical Dictionary for Regulatory Activities
NE	Not evaluable
OS	Overall survival
pCR	Pathological Complete Response
PD	Progression disease
PFS	Progression-free survival
PLT	Platelet count
PR	Partial response
PS	Performance status
PT	Preferred term
QTc	Q-T interval corrected for heart rate
RECIST	Response Evaluation Criteria in Solid Tumors
RS	Raw score
SAE	Serious adverse events
SAP	Statistical analysis plan
SAS	Statistical Analysis System
SD	Stable disease
SOC	System organ class

Abbreviation	Definition
SS	Survival analysis set
TBIL	Total bilirubin
TCR	T Cell Receptor
TEAE	Treatment-emergent adverse event
TMB	Tumor mutation burden
TRAE	Treatment-related TEAE
TTR	Time to response
WBC	White blood cells

17.2 TNM staging system for esophageal cancer (8th edition)

17.2.1 Definitions for T, N, M

T	Primary Tumor
TX	Primary tumor cannot be assessed
T0	No evidence of primary tumor
Tis	High-grade dysplasia, defined as malignant cells confined to the epithelium by the basement membrane
T1	Tumor invades the lamina propria, muscularis mucosae, or submucosa
T1a	Tumor invades the lamina propria or muscularis mucosae
T1b	Tumor invades the submucosa
T2	Tumor invades the muscularis propria
T3	Tumor invades adventitia
T4	Tumor invades adjacent structures
T4a	Tumor invades the pleura, pericardium, azygos vein, diaphragm, or peritoneum
T4b	Tumor invades other adjacent structures, such as the aorta, vertebral body, or airway

N	Regional Lymph Nodes
NX	Regional lymph nodes cannot be assessed
N0	No regional lymph node metastasis
N1	Metastasis in one or two regional lymph nodes
N2	Metastasis in three to six regional lymph nodes
N3	Metastasis in seven or more regional lymph nodes

M	Distant Metastasis
M0	No distant metastasis
M1	Distant metastasis

G	Histologic Grade
GX	Grade cannot be assessed
G1	Well differentiated
G2	Moderately differentiated
G3	Poorly differentiated, undifferentiated

Squamous Cell Carcinoma

Location	Location Criteria
X	Location unknown
Upper	Cervical esophagus to lower border of azygos vein
Middle	Lower border of azygos vein to lower border of inferior pulmonary vein
Lower	Lower border of inferior pulmonary vein to stomach, including gastroesophageal junction

Note: Location is defined by the position of the epicenter of the tumor in the esophagus.

17.2.2 AJCC Prognostic Stage Groups (Squamous Cell Carcinoma)

Clinical Staging (cTNM)

	cT	cN	M
Stage 0	Tis	N0	M0
Stage I	T1	N0-1	M0
Stage II	T2	N0-1	M0
	T3	N0	M0
Stage III	T3	N1	M0
	T1-3	N2	M0
Stage IVA	T4	N0-2	M0
	Any T	N3	M0
Stage IVB	Any T	Any N	M1

Pathological (pTNM)

	pT	pN	M	G	Location
Stage 0	Tis	N0	M0	N/A	Any
Stage IA	T1a	N0	M0	G1	Any
	T1a	N0	M0	GX	Any
Stage IB	T1a	N0	M0	G2-3	Any
	T1b	N0	M0	G1-3	Any
	T1b	N0	M0	GX	Any
	T2	N0	M0	G1	Any
Stage IIA	T2	N0	M0	G2-3	Any
	T2	N0	M0	GX	Any
	T3	N0	M0	G1-3	Lower
	T3	N0	M0	G1	Upper/middle
Stage IIB	T3	N0	M0	G2-3	Upper/middle
	T3	N0	M0	GX	Lower/upper/ middle
	T3	N0	M0	Any	Location X
	T1	N1	M0	Any	Any
Stage IIIA	T1	N2	M0	Any	Any
	T2	N1	M0	Any	Any
Stage IIIB	T2	N2	M0	Any	Any
	T3	N1-2	M0	Any	Any
	T4a	N0-1	M0	Any	Any
Stage IVA	T4a	N2	M0	Any	Any
	T4b	N0-2	M0	Any	Any
	Any T	N3	M0	Any	Any
Stage IVB	Any T	Any N	M1	Any	Any

Postneoadjuvant Therapy (ypTNM)

	ypT	ypN	M
Stage I	T0-2	N0	M0
Stage II	T3	N0	M0
Stage IIIA	T0-2	N1	M0
Stage IIIB	T3	N1	M0
	T0-3	N2	M0
	T4a	N0	M0
Stage IVA	T4a	N1-2	M0
	T4a	NX	M0
	T4b	N0-2	M0
	Any T	N3	M0
Stage IVB	Any T	Any N	M1

Continued

17.3 ECOG Performance Status scale

Score	Definition
0	Asymptomatic
1	Symptomatic, fully ambulatory
2	Symptomatic, in bed less than 50% of day
3	Symptomatic, in bed more than 50% of day, but not bedridden
4	Bedridden

17.4 Time point response: patients with target (+/- non-target) disease

Target lesions	Nontarget lesions	New lesions	Overall response
CR	CR	No	CR
CR	non-CR/non-PD	No	PR
CR	Not fully evaluable	No	PR
PR	non-PD or not fully evaluable	No	PR
SD	non-PD or not fully evaluable	No	SD
Not fully evaluable	non-PD	No	NE
PD	Any	Yes or no	PD
Any	PD	Yes or no	PD
Any	Any	Yes	PD
CR = complete response; PR = partial response; SD = stable disease; PD = progressive disease; NE =non-evaluable			

17.5 Time point response: patients with non-target disease only

Nontarget lesions	New lesions	Overall response
CR	No	CR
Non-CR or non-PD	No	Non-CR or non-PD
Not fully evaluable	No	NE
Unconfirmed PD	Yes or no	PD
Any	Yes	PD

17.6 INVESTIGATOR'S BROCHURE

18 Reference

1. H. Sung *et al.*, Global Cancer Statistics 2020: GLOBOCAN Estimates of Incidence and Mortality Worldwide for 36 Cancers in 185 Countries. *CA Cancer J Clin* **71**, 209-249 (2021).
2. A. K. Rustgi, H. B. El-Serag, Esophageal carcinoma. *N Engl J Med* **371**, 2499-2509 (2014).
3. A. Pennathur, M. K. Gibson, B. A. Jobe, J. D. Luketich, Oesophageal carcinoma. *Lancet* **381**, 400-412 (2013).
4. J. Lagergren, E. Smyth, D. Cunningham, P. Lagergren, Oesophageal cancer. *Lancet* **390**, 2383-2396 (2017).
5. M. A. Shah *et al.*, Treatment of Locally Advanced Esophageal Carcinoma: ASCO Guideline. *J Clin Oncol* **38**, 2677-2694 (2020).
6. M. D. Vesely, M. H. Kershaw, R. D. Schreiber, M. J. Smyth, Natural innate and adaptive immunity to cancer. *Annu Rev Immunol* **29**, 235-271 (2011).
7. J. S. O'Donnell, M. W. L. Teng, M. J. Smyth, Cancer immunoediting and resistance to T cell-based immunotherapy. *Nat Rev Clin Oncol* **16**, 151-167 (2019).
8. J. M. Sun *et al.*, Pembrolizumab plus chemotherapy versus chemotherapy alone for first-line treatment of advanced oesophageal cancer (KEYNOTE-590): a randomised, placebo-controlled, phase 3 study. *Lancet* **398**, 759-771 (2021).
9. Y. Doki *et al.*, Nivolumab Combination Therapy in Advanced Esophageal Squamous-Cell Carcinoma. *N Engl J Med* **386**, 449-462 (2022).
10. Z. X. Wang *et al.*, Toripalimab plus chemotherapy in treatment-naïve, advanced esophageal squamous cell carcinoma (JUPITER-06): A multi-center phase 3 trial. *Cancer Cell* **40**, 277-288.e273 (2022).
11. R. Yu *et al.*, RATIONALE 311: tislelizumab plus concurrent chemoradiotherapy for localized esophageal squamous cell carcinoma. *Future Oncol* **17**, 4081-4089 (2021).
12. K. Kato *et al.*, Nivolumab versus chemotherapy in patients with advanced oesophageal squamous cell carcinoma refractory or intolerant to previous chemotherapy (ATTRACTION-3): a multicentre, randomised, open-label, phase 3 trial. *Lancet Oncol* **20**, 1506-1517 (2019).
13. T. Kojima *et al.*, Randomized Phase III KEYNOTE-181 Study of Pembrolizumab Versus Chemotherapy in Advanced Esophageal Cancer. *J Clin Oncol* **38**, 4138-4148 (2020).
14. J. Huang *et al.*, Camrelizumab versus investigator's choice of chemotherapy as second-line therapy for advanced or metastatic oesophageal squamous cell carcinoma (ESCORT): a multicentre, randomised, open-label, phase 3 study. *Lancet Oncol* **21**, 832-842 (2020).
15. R. J. Kelly *et al.*, Adjuvant Nivolumab in Resected Esophageal or Gastroesophageal Junction Cancer. *N Engl J Med* **384**, 1191-1203 (2021).

16. C. Li *et al.*, Preoperative pembrolizumab combined with chemoradiotherapy for oesophageal squamous cell carcinoma (PALACE-1). *Eur J Cancer* **144**, 232-241 (2021).
17. X. Shang *et al.*, LBA3 Safety and efficacy of pembrolizumab combined with paclitaxel and cisplatin as a neoadjuvant treatment for locally advanced resectable (stage III) esophageal squamous cell carcinoma (Keystone-001): Interim analysis of a prospective, single-arm, single-center, phase II trial. *Annals of Oncology* **32**, S1428-S1429 (2021).
18. J. Ma *et al.*, 65P Camrelizumab combined with paclitaxel and nedaplatin as neoadjuvant therapy for locally advanced esophageal squamous cell carcinoma (ESPRIT): A phase II, single-arm, exploratory research. *Annals of Oncology* **32**, S1400 (2021).
19. X. Yan *et al.*, Tislelizumab combined with chemotherapy as neoadjuvant therapy for surgically resectable esophageal cancer: A prospective, single-arm, phase II study (TD-NICE). *Int J Surg* **103**, 106680 (2022).
20. W. C. Dempke, K. Fenchel, P. Uciechowski, S. P. Dale, Second-and third-generation drugs for immuno-oncology treatment—the more the better? *European Journal of Cancer* **74**, 55-72 (2017).
21. Y. Jiang, M. Chen, H. Nie, Y. Yuan, PD-1 and PD-L1 in cancer immunotherapy: clinical implications and future considerations. *Human vaccines & immunotherapeutics* **15**, 1111-1122 (2019).
22. J. J. Havel, D. Chowell, T. A. Chan, The evolving landscape of biomarkers for checkpoint inhibitor immunotherapy. *Nat Rev Cancer* **19**, 133-150 (2019).
23. J. M. Chemnitz, R. V. Parry, K. E. Nichols, C. H. June, J. L. Riley, SHP-1 and SHP-2 associate with immunoreceptor tyrosine-based switch motif of programmed death 1 upon primary human T cell stimulation, but only receptor ligation prevents T cell activation. *J Immunol* **173**, 945-954 (2004).
24. K. A. Sheppard *et al.*, PD-1 inhibits T-cell receptor induced phosphorylation of the ZAP70/CD3zeta signalosome and downstream signaling to PKCtheta. *FEBS Lett* **574**, 37-41 (2004).
25. M. Yarchoan *et al.*, PD-L1 expression and tumor mutational burden are independent biomarkers in most cancers. *JCI Insight* **4**, (2019).
26. C. Denkert *et al.*, Clinical and molecular characteristics of HER2-low-positive breast cancer: pooled analysis of individual patient data from four prospective, neoadjuvant clinical trials. *Lancet Oncol* **22**, 1151-1161 (2021).
27. P. M. Forde *et al.*, Neoadjuvant PD-1 Blockade in Resectable Lung Cancer. *N Engl J Med* **378**, 1976-1986 (2018).
28. E. Ahern *et al.*, Neoadjuvant immunotherapy for non-small cell lung cancer: right drugs, right patient, right time? *J Immunother Cancer* **9**, (2021).
29. R. N. Amaria *et al.*, Neoadjuvant immune checkpoint blockade in high-risk resectable melanoma. *Nat Med* **24**, 1649-1654 (2018).
30. S. L. Topalian, J. M. Taube, D. M. Pardoll, Neoadjuvant checkpoint blockade for cancer immunotherapy. *Science* **367**, (2020).
31. J. Wang *et al.*, Adebrelimab or placebo plus carboplatin and etoposide as

first-line treatment for extensive-stage small-cell lung cancer (CAPSTONE-1): a multicentre, randomised, double-blind, placebo-controlled, phase 3 trial. *Lancet Oncol* **23**, 739-747 (2022).

32. W. Yan *et al.*, Adebrelimab (SHR-1316) in combination with chemotherapy as perioperative treatment in patients with resectable stage II-III NSCLC: an open-label, multicenter, phase 1b trial. *J Thorac Oncol*, (2022).
33. L. Mu *et al.*, SHR-1316, an anti-PD-L1 antibody, plus chemotherapy as the first-line treatment for advanced esophageal squamous cell carcinoma: A multicentre, phase 2 study. *Thorac Cancer* **12**, 1373-1381 (2021).
34. M. A. Patel, J. D. Kratz, S. J. Lubner, N. K. Loconte, N. V. Uboha, Esophagogastric Cancers: Integrating Immunotherapy Therapy Into Current Practice. *J Clin Oncol* **40**, 2751-2762 (2022).
35. T. Kojima *et al.*, Randomized Phase III KEYNOTE-181 Study of Pembrolizumab Versus Chemotherapy in Advanced Esophageal Cancer. *Journal of clinical oncology : official journal of the American Society of Clinical Oncology* **38**, 4138-4148 (2020).
36. J. Huang *et al.*, Camrelizumab versus investigator's choice of chemotherapy as second-line therapy for advanced or metastatic oesophageal squamous cell carcinoma (ESCORT): a multicentre, randomised, open-label, phase 3 study. *The Lancet. Oncology* **21**, 832-842 (2020).
37. G. Terrero, A. C. Lockhart, Role of Immunotherapy in Advanced Gastroesophageal Cancer. *Curr Oncol Rep* **22**, 112 (2020).
38. Y. Zheng *et al.*, A phase III study on neoadjuvant chemotherapy versus neoadjuvant toripalimab plus chemotherapy for locally advanced esophageal squamous cell carcinoma: Henan Cancer Hospital Thoracic Oncology Group 1909 (HCHTOG1909). *Ann Transl Med* **9**, 73 (2021).
39. X. Shang *et al.*, Pembrolizumab Combined With Neoadjuvant Chemotherapy Versus Neoadjuvant Chemoradiotherapy Followed by Surgery for Locally Advanced Oesophageal Squamous Cell Carcinoma: Protocol for a Multicentre, Prospective, Randomized-Controlled, Phase III Clinical Study (Keystone-002). *Front Oncol* **12**, 831345 (2022).
40. T. van den Ende *et al.*, Neoadjuvant Chemoradiotherapy Combined with Atezolizumab for Resectable Esophageal Adenocarcinoma: A Single-arm Phase II Feasibility Trial (PERFECT). *Clin Cancer Res* **27**, 3351-3359 (2021).
41. D. Salas-Benito *et al.*, Paradigms on Immunotherapy Combinations with Chemotherapy. *Cancer Discov* **11**, 1353-1367 (2021).
42. H. Wang, S. Li, T. Liu, J. Chen, J. Dang, Neoadjuvant immune checkpoint inhibitor in combination with chemotherapy or chemoradiotherapy in resectable esophageal cancer: A systematic review and meta-analysis. *Front Immunol* **13**, 998620 (2022).
43. L. A. Emens, G. Middleton, The interplay of immunotherapy and chemotherapy: harnessing potential synergies. *Cancer Immunol Res* **3**, 436-443 (2015).
44. M. Feng *et al.*, Thoracolaparoscopic esophagectomy: is the prone position

a safe alternative to the decubitus position? *J Am Coll Surg* **214**, 838-844 (2012).
45. Y. Shen *et al.*, A simple method minimizes chylothorax after minimally invasive esophagectomy. *J Am Coll Surg* **218**, 108-112 (2014).
46. Y. Shen *et al.*, Extensive mediastinal lymphadenectomy during minimally invasive esophagectomy: optimal results from a single center. *J Gastrointest Surg* **16**, 715-721 (2012).
47. T. Shimoda, Japanese classification of esophageal cancer, the 10th edition--Pathological part. *Nihon rinsho. Japanese Journal of Clinical Medicine* **69**, 109-120 (2011).
