# Supplementary material for: Neoadjuvant adebrelimab in locally advanced resectable esophageal squamous cell carcinoma: a phase 1b trial
Source: Nat Med. 2023 Jul 24;29(8):2068–78. doi: 10.1038/s41591-023-02469-3 (PMC10427424; doi:10.1038/s41591-023-02469-3)
Supplement: Supplementary file 2 — Reporting Summary [file 41591_2023_2469_MOESM2_ESM.pdf]

nature portfolio

Corresponding author(s): Qing Zhou

Last updated by author(s): Jun 16, 2023

Reporting Summary

Nature Portfolio wishes to improve the reproducibility of the work that we publish. This form provides structure for consistency and transparency in reporting. For further information on Nature Portfolio policies, see our Editorial Policies and the Editorial Policy Checklist.

Statistics

For all statistical analyses, confirm that the following items are present in the figure legend, table legend, main text, or Methods section.

n/a	Confirmed	
☐	☒	The exact sample size (*n*) for each experimental group/condition, given as a discrete number and unit of measurement
☐	☒	A statement on whether measurements were taken from distinct samples or whether the same sample was measured repeatedly
☐	☒	The statistical test(s) used AND whether they are one- or two-sided<br>*Only common tests should be described solely by name; describe more complex techniques in the Methods section.*
☐	☒	A description of all covariates tested
☐	☒	A description of any assumptions or corrections, such as tests of normality and adjustment for multiple comparisons
☐	☒	A full description of the statistical parameters including central tendency (e.g. means) or other basic estimates (e.g. regression coefficient) AND variation (e.g. standard deviation) or associated estimates of uncertainty (e.g. confidence intervals)
☐	☒	For null hypothesis testing, the test statistic (e.g. *F*, *t*, *r*) with confidence intervals, effect sizes, degrees of freedom and *P* value noted<br>*Give P values as exact values whenever suitable.*
☒	☐	For Bayesian analysis, information on the choice of priors and Markov chain Monte Carlo settings
☒	☐	For hierarchical and complex designs, identification of the appropriate level for tests and full reporting of outcomes
☐	☒	Estimates of effect sizes (e.g. Cohen's *d*, Pearson's *r*), indicating how they were calculated

Our web collection on statistics for biologists contains articles on many of the points above.

Software and code

Policy information about availability of computer code

Data collection	MGISEQ-2000/DNBSEQ T1 sequencer system(MGI)<br>Polaris imaging system(Akoya Biosciences/PerkinElmer)<br>nCounter Digital analyzer(Nanostring)
Data analysis	Phenochart (v1.1.0), STAR (v2.5.1b), BWA v0.7.12, Picard Tools v1.84, GATK v4.1, SomaticSniper v1.0.5.1, MutTect2 v2.7.0, MuSE v1.0, Strelka v2.9.9, Svaba v0.2.1, ANNOVAR v180504, deconstructSigs v1.8.0, FACETS v0.16.0, GISTIC v2.0.23, Polysolver v1.0, netMHC v4.0, netMHCpan v4.1, MHCflurry v2.0.4, MixMHCpred v2.1, HLAthena v1.0, pMTnet v1.0.0, MSIsensor (v0.6), DESeq2 (v1.30), ClusterProfiler (v4.4.4), MSigDB(v7.4.),GSVA (v1.46), EnhancedVolcano (v1.14.0), glmnet (v4.1.4), CIBERSORTx (v1.0.4), Ecotyper (v1.0), ESTIMATE (v1.0.13), heatmap (v1.0.12), VDJtools (v1.2.1),T cell ExTRECT (v1.0.1), ipw (v1.2), R (v4.1.1), python (v3.7.9), survminer (v0.4.9), gtsummary (v1.6.2), survival (v3.4), purrr (v0.3.5), plyr (v1.8.8), tidyr (v1.2.1),dplyr (v1.0.10), ggsignif (v0.6.4), ggplot2 (v3.4.0), cowplot (v1.1.1), ggalluvial (v0.12.3), RColorBrewer (v1.1.3), ggrepel (v0.9.2), ggthemes (v4.2.4), ggpubr (v0.5.0)<br><br>NanoString analysis: nCounter Digital Analyzer was used to tabulate the counts of the reporter probes and for further analysis raw data output was imported into nSolver analysis software (v4.0.70) (http://www.nanostring.com/products/nSolver). Normalization, cell type and differential gene expression analyses were performed using the nSolver Advanced data analysis package (v2.0.134).<br><br>Codes used for bulk RNA-seq, bulk TCR-seq and WES analysis are available from https://github.com/yuanjingnan/ESCC-code/tree/main

For manuscripts utilizing custom algorithms or software that are central to the research but not yet described in published literature, software must be made available to editors and reviewers. We strongly encourage code deposition in a community repository (e.g. GitHub). See the Nature Portfolio guidelines for submitting code & software for further information.

Data

Policy information about availability of data

All manuscripts must include a data availability statement. This statement should provide the following information, where applicable:

- Accession codes, unique identifiers, or web links for publicly available datasets
- A description of any restrictions on data availability
- For clinical datasets or third party data, please ensure that the statement adheres to our policy

De-identified raw sequencing data of participated patients were deposited in CNGB Nucleotide Sequence Archive (CNSA) with accession codes CNP0002585, CNP0003632, CNP0003659. Datasets of this clinical trial can be requested 12 months after this article published. Researchers who request access to raw and analyzed data should send email to corresponding author Q. Zhou (zhouqing2@genomics.cn) and K. Wu (wukui@genomics.cn) to clarify research purpose, and will be reviewed by BGI Institutional Review Board, considering the risk of patient re-identification. Data are available for approved eligible applications and investigators, after signing a data access agreement. Source data are provided with this paper.
GRCh38 reference genome was used for alignment of filter sequences.

Human research participants

Policy information about studies involving human research participants and Sex and Gender in Research.

Reporting on sex and gender	Self-reported gender is recorded and supplied in Table1 and ExtendedData Table 2. 88% patients identified was male, this imbalance is due to the nature of the disease studied. no gender-based analysis was performed.<br>There was no bias towards age, gender or race in this clinical trial outlined. This trial was open to the accrual of men and women who meet the inclusion and exclusion criteria outlined.
Population characteristics	Clinical stage II-IV esophageal squamous cell carcinoma with resectable disease, Patients aged 18 and over, ECOG PS 0-1, normal organ function with no contra-indications to surgery. Detailed patient characteristics is supplied in Table 1
Recruitment	Patients were enrolled at Zhongshan Hospital, Fudan University. Patients were offered either clinical trial enrollment or standard of care therapies. Patients were provided copies of the study informed consent document and were fully aware risks prior to trial enrollment. As patients needed to fulfill inclusion criteria of trial, this could have caused selection bias. We provided the drug (adebrelimab) and medical examination for free, but there was no additional participant compensation.
Ethics oversight	IRB of Research Ethics Committee of Zhongshan Hospital of Fudan University and BGI Research provided ethics oversight. An informed consent statement was included in the Methods/Study design and inventions

Note that full information on the approval of the study protocol must also be provided in the manuscript.

Field-specific reporting

Please select the one below that is the best fit for your research. If you are not sure, read the appropriate sections before making your selection.

☒ Life sciences ☐ Behavioural & social sciences ☐ Ecological, evolutionary & environmental sciences

For a reference copy of the document with all sections, see nature.com/documents/nr-reporting-summary-flat.pdf

Life sciences study design

All studies must disclose on these points even when the disclosure is negative.

Sample size	This was a single-arm study, a total of 30 patients were enrolled without randomization in this study. About sample size, a Simon optimal two-stage design was used. 70% of feasibility was considered unacceptable, and 90% of feasibility was considered promising. This design allowed early study termination for excessive surgery delay. The probabilities of type I and type II errors were set at 0.05 and 0.2, respectively.<br>Six patients will be accrued to the first stage, and if five or more patients proceed to surgery without extended treatment related delays, 21 patients would be enrolled on the second stage. If more than 23 of the 27 patients proceed to surgery without extended treatment related delays, this regimen would be considered worthy of further testing.
Data exclusions	There were no data exclusions
Replication	This was a non-randomized phase 1b clinical study to collect preliminary safety and overall survival data. Data was not able to be replicated as this was a study using human subjects and data generated was unique to the study project. Replication of these results will be sought in the follow-up phase II and III studies. Translational experiments on human samples were not replicated due to limited tumor specimen, because one tumor sample had to simultaneously performed for multi-omics analysis, including whole exome sequencing, bulk RNA sequencing, TCR sequencing, PD-L1 (22C3) staining, immune target RNA sequencing, and et al. after meeting the basic requirement of pathological examination. We further validated our findings in nine published pan-cancer datasets and TCGA-ESCC datasets.
Randomization	This was a single arm study with no randomization

Blinding | Blinding was not utilized as this is a single arm, non-randomized trial

Reporting for specific materials, systems and methods

We require information from authors about some types of materials, experimental systems and methods used in many studies. Here, indicate whether each material, system or method listed is relevant to your study. If you are not sure if a list item applies to your research, read the appropriate section before selecting a response.

Materials & experimental systems

n/a	Involved in the study
☐	☒ Antibodies
☒	☐ Eukaryotic cell lines
☒	☐ Palaeontology and archaeology
☒	☐ Animals and other organisms
☐	☒ Clinical data
☒	☐ Dual use research of concern

Methods

n/a	Involved in the study
☒	☐ ChIP-seq
☒	☐ Flow cytometry
☒	☐ MRI-based neuroimaging

Antibodies

Antibodies used

The anti-PD-L1 antibody adebrelimab was provided by the study sponsor Hengrui Company as part of their investigational supply of agents. Adebrelimab should be stored at 2 °C to 8 °C (36oF to 46oF) with protection from light. Do not freeze the drug product. Adebrelimab should be administered as 60 minutes intravenously infusion through a 0.2/1.2 pore size, low-protein binding polyethersulfone membrane in-line filter at the protocol-specified doses. Detailed instructions for drug product dilution and administration are provided in the pharmacy manual for the clinical study.

Multiplex immunofluorescence studies: antibodies against: panel 1: CD4 (clone EPR6855, dilution 1:100, Cat: Ab133616, Abcam), CD8 (polyclones, dilution 1:200, cat. NBP2-34039, Novus), FOXP3 (clone 236A/E7, dilution 1:200, Cat: Ab20034, Abcam), PD-L1 (clone EPR19759, dilution 1:200, Cat: Ab213524, Abcam), PD-1 (clone NAT105, dilution 1:200, Cat: Ab52587, Abcam); panel 2: CD20 (clone L26, dilution 1:200, Cat: Ab9475, Abcam), CD11c (clone EP347Y, dilution 1:500, Cat. Ab52632, Abcam), CD68 (clone 968, dilution 1:400, Cat: 76437S, Cell Signaling Technology), CD163 (clone EPR19518, dilution 1:100, Cat: Ab182422, Abcam), IFNγ (polyclones, dilution 1:200, Cat: Ab25101, Abcam); panel 3: CD3 (clone SP7, dilution 1:200, Cat: Ab16669, Abcam), Pan-CK (clone C-11, dilution 1:800, Cat. Ab7753, Abcam), Vimentin (clone EPR3776, dilution 1:600, Cat: Ab92547, Abcam), Ki-67 (clone SP6, dilution 1:100, Cat: Ab16667, Abcam).

Validation

Anti-PD-L1 antibodies were provided as part of Hengrui Company's investigational study supply.
all antibodies used in the mIF assay were commercially available.
1. Anti-CD4, Mouse mAb, Abcam, Cat: ab133616, Clone: EPR6855: https://www.abcam.cn/cd4-antibody-epr6855-ab133616.html
2. Anti-CD8, Rabbit mAb, Abcam, Cat: ab217344, Clone: EPR21769 https://www.abcam.cn/cd8-alpha-antibody-epr21769-ab217344.html
3. Anti-FOXP3, Mouse mAb, Abcam, Cat: ab20034, Clone: 236A/E7 https://www.abcam.cn/foxp3-antibody-236ae7-ab20034.html
4. Anti-PD-L1, Mouse mAb, Abcam, Cat: ab213524, Clone: EPR19759 https://www.abcam.cn/pd-l1-antibody-epr19759-ab213524.html
5. Anti-PD-1, Mouse mAb, Abcam, Cat: ab52587, Clone: NAT105 https://www.abcam.cn/pd1-antibody-nat105-ab52587.html
6. Anti-CD68, Mouse mAb, CST, Cat: 76437S, Clone:968 https://www.cellsignal.cn/products/primary-antibodies/cd68-d4b9c-xp-rabbit-mab/76437?site-search-type=Products&N=4294956287&Ntt=76437s&fromPage=plp&_requestid=841564
7. Anti-CD163, Mouse mAb, Abcam, Cat: ab182422, Clone: EPR19518 https://www.abcam.cn/cd163-antibody-epr19518-ab182422.html
8. Anti-CD20, Mouse mAb, Abcam, Cat: ab9475, Clone: L26 https://www.abcam.cn/cd20-antibody-l26-ab9475.html
9. Anti-CD11c Mouse mAb, Abcam, Cat: ab52632, Clone: EP1347Y https://www.abcam.cn/cd11c-antibody-ep1347y-c-terminal-ab52632.html
10. Anti-IFNγ, Rabbit mAb, Abcam, Cat: ab231036, Clone: EPR21704 https://www.abcam.cn/interferon-gamma-antibody-epr21704-ab231036.html
11. Anti-CD3, Mouse mAb, Abcam, Cat:ab16669, Clone: SP7 https://www.abcam.cn/cd3-antibody-sp7-ab16669.html
12. Anti-pan-CK, Mouse mAb, Abcam, Cat:ab7753, Clone: C-11 https://www.abcam.cn/pan-cytokeratin-antibody-c-11-ab7753.html
13. Anti-vimentin, Mouse mAb, Abcam, Cat:ab92547, Clone: EPR3776 https://www.abcam.cn/vimentin-antibody-epr3776-cytoskeleton-marker-ab92547.html
14. Anti-Ki67, Rabbit mAb, Abcam, Cat: ab16667, Clone:SP6 https://www.abcam.cn/ki67-antibody-sp6-ab16667.html

Clinical data

Policy information about clinical studies
All manuscripts should comply with the ICMJE guidelines for publication of clinical research and a completed CONSORT checklist must be included with all submissions.

Clinical trial registration | NCT04215471

Study protocol | Study protocol is supplied as an appendix in Supplementary Information files.

Data collection	12/26/2019-8/29/2020 for enrollment, patients followed for at least 2 year after data of last enrollment. Data was stored in a secure database in Cancer center of Zhongshan Hospital of Fudan University and was able to be assessed by staff at Cancer center of Zhongshan Hospital of Fudan University for direct data input.
Outcomes	The primary endpoints were the feasibility and safety. The second endpoint was pCR rate, OS, RFS and R0 rate. correlation of genomic profiling and immune profiling with response were exploratory.
